# Clinical Efficacy and Safety of Traditional Medicine Preparations Combined With Chemotherapy for Advanced Pancreatic Cancer: A Systematic Review and Meta-Analysis

**DOI:** 10.3389/fonc.2022.828450

**Published:** 2022-02-23

**Authors:** Jiaqi Hu, Juling Jiang, Rui Liu, Mengqi Cheng, Guanghui Zhu, Shulin He, Bolun Shi, Yuwei Zhao, Zhongning He, Huibo Yu, Xing Zhang, Honggang Zheng, Baojin Hua

**Affiliations:** ^1^ Department of Oncology, Guang’anmen Hospital, China Academy of Chinese Medical Sciences, Beijing, China; ^2^ Graduate School, Beijing University of Chinese Medicine, Beijing, China; ^3^ Xiyuan Hospital, China Academy of Chinese Medical Sciences, Beijing, China

**Keywords:** advanced pancreatic cancer, traditional medicine preparations, chemotherapeutic therapy, systematic review, meta-analysis

## Abstract

**Background:**

Traditional medicine preparations (TMPs) combined with chemotherapy is widely used for patients with advanced pancreatic cancer (APC); however, its efficacy and safety are still unclear. The purpose of this meta-analysis was to evaluate the clinical efficacy and safety of TMPs combined with chemotherapy for the treatment of APC.

**Methods:**

A systematic search of eight electronic databases for randomized controlled trials (RCTs) was conducted from inception to October 15, 2021. Tumor response was identified as primary outcome, whereas quality of life (QoL), cancer biomarkers, and adverse drug reactions (ADRs) were identified as secondary outcomes. Quality of the evidence for each outcome was evaluated by GRADE profiler.

**Results:**

In total, 31 RCTs involving 1,989 individuals were included. This meta-analysis showed that TMPs combined with chemotherapy significantly improved the objective response rate (ORR) (RR=1.64, 95% CI [1.43 to 1.88], p <0.00001), disease control rate (DCR) (RR=1.29, 95% CI [1.21 to 1.38], p <0.00001), and QoL (continuous data: SMD=0.81, 95% CI [0.44 to 1.18], p <0.0001, dichotomous data: RR=1.44, 95% CI [1.22 to 1.70], p<0.0001), compared to those with chemotherapy alone. In addition, the combined treatment group also had lower levels of CA19-9 (SMD=-0.46, 95% CI [-0.90 to -0.02], *p*=0.04) and CEA (SMD=-0.55, 95% CI [-0.93 to -0.17], *p*=0.004). Moreover, TMPs reduced the ADRs during chemotherapy.

**Conclusion:**

This systematic review suggests that TMPs combined with chemotherapy might be a potential option to enhance therapeutic effects and reduce ADRs during the treatment of APC. However, more high-quality randomized controlled trials with more participants are needed.

**Systematic Review Registration:**

https://www.crd.york.ac.uk/prospero/display_record.php?RecordID=209825, identifier PROSPERO Number: CRD42021264938.

## 1 Introduction

Pancreatic cancer is recognized as a highly deadly malignant tumor with approximately equivalent number of new cases (496,000) and deaths (466,000 cases) annually (1). Remarkably, the incidence of pancreatic cancer has increased significantly by 39.3% between 2007 and 2017, thus ranking among top five actively growing cancers worldwide (2). Despite its 5-year survival rates are only 10-25% (3, 4), and surgery remains the only possible cure for pancreatic cancer. Unfortunately, the sobering reality is that only less than 20% of patients have a chance of undergoing surgery due to the lack of prominent symptoms at early stages of this disease (5) and the most patients are often diagnosed with local vessel involvement or distant metastases. Systemic chemotherapy plays an important role for the management of advanced pancreatic cancer (APC) and can aid to prolong survival (6). However, the median total survival of APC is only approximately 6 to 11 months (7, 8). Meanwhile, several adverse drug reactions (ADRs) of chemotherapy (neutropenia, anemia, neurotoxicity etc.) have severely affected the treatment outcome of patients with APC (9, 10). Thus, APC contributes to substantial burden to individuals and society.

In order to prolong the long-term survival while preserving the quality of life (QoL) of patients, the search for novel complementary treatment combined with chemotherapy becomes crucial.

Traditional medicine preparations (TMPs) are defined as any formulation of medicinal herbs including extracts of herbs, herbal injection, Chinese proprietary medicine, or self-prepared herbal decoctions prescribed by practitioners, with the advantages of easy availability, low price, and generally exhibit few ADRs. A number of experimental studies have shown that several plant extracts such as curcumin (11), bitter melon juice (12), elemene (13) etc. can exhibit significant efficacy against different cancers. These natural compounds function as potent anti-neoplastic agents by interfering with multiple cellular processes, but limited chemical stability and oral bioavailability have hampered their rapid clinical translation which might be improved by nanoformulations (11). There are several reports in literature describing the beneficial effects of many TMPs in cancer treatment for improving clinical efficacy and safety (14–16).

Over the past 20 years, some clinical studies have been published describing the application of TMPs for the treatment of APC but their findings are relatively less convincing because of the potential use of small sample size. Therefore, we have performed a meta-analysis to systematically analyze the results of these prior studies, which aimed to evaluate the efficacy and safety of TMPs combined with chemotherapy for APC, thus hoping to provide an important reference for the clinicians.

## 2 Methods

### 2.1 Study Design

This systemic review and meta-analysis strictly followed the Preferred Reporting Items for Systematic Reviews and Meta-Analyses Guidelines (17). Its registered number in PROSPERO is CRD42020209825.

### 2.2 Inclusion and Exclusion Criteria

#### 2.2.1 Inclusion Criteria

##### 2.2.1.1 Patients

Patients diagnosed with unresectable (locally advanced and/or metastatic) or stage III–IV pancreatic cancer through the histological and cytological diagnostic criteria, and TNM staging systems were included. The baseline data of patients in the two groups were comparable. There were no restrictions on age or sex.

##### 2.2.1.2 Interventions

The experimental group received TMPs combined with chemotherapy. The TMPs included extracts of herbs, patented herbal products, or self-prepared herbal decoctions prescribed by practitioners. The administration or formulation of TMPs including decoction, granule, capsule, or injection were not limited. The control group received the same chemotherapy regimen alone.

##### 2.2.1.3 Primary Outcome

The primary outcome was tumor response assessed using the objective response rate (ORR) and disease control rate (DCR), measured separately before the start of each trial and at the end of the follow-up time, according to the WHO (18) and RECIST (19) criteria. Trials not stating evaluation criteria were also included and subgroup analysis was carried out thereafter.

##### 2.2.1.4 Secondary Outcomes

The secondary outcomes were defined as the QoL, cancer biomarkers, and ADRs. The interventions were considered to be effective for QoL when the Karnofsky Performance Status (KPS) score was no more than 10 points lower after treatment. Comparisons were also made for the mean ± standardized difference (SD) of KPS scores before and after treatment was also allowed. Cancer biomarker levels were assessed by measuring the CA19-9 and CEA levels separately before the start of each trial and at the end of the follow-up time. The mean ± SD changes in CA19-9 and CEA levels were synthesized to evaluate the differences between the two groups. ADRs were evaluated by calculating the number of people at stage 0-IV cancer experiencing gastrointestinal toxicity (nausea, vomiting, and diarrhea), myelosuppression (leukopenia, decreased hemoglobin, and thrombopenia), hair loss, liver dysfunction, and renal dysfunction, according to the WHO or NCI recommendations for grading acute and subacute toxicity. The interventions were considered to lead to ADRs when patients had levels III-IV.

##### 2.2.1.5 Types of Studies

All published randomized controlled trials (RCTs) published were included. Quasi-randomized trials were excluded. Only full journal publications with sufficient data for analysis were included. The language was not restricted.

#### 2.2.2 Exclusion Criteria

The exclusion criteria were the following: (1) simultaneous other types of primary tumors; (2) the TMPs were not fixed within 1 study; (3) unspecified or inconsistent observation nodes between two groups within 1 study; (4) insufficient data; (5) irregular outcome evaluation criteria; and (6) duplicated data.

### 2.3 Search Strategy for the Identification of Studies

RCTs were searched from inception to October 15, 2021, in the following electronic databases: PubMed, EMBASE, the Cochrane Library, clinicaltrials.gov, Trip Database, Allied and Complementary Medicine Database (AMED), Latin American and Caribbean Health Sciences Literature (LILACS), China National Knowledge Infrastructure (CNKI), Chinese Scientific Journal Database (VIP database), Wangfang Data Knowledge Service Platform, and Chinese Biomedical Literature Database (CBM). The following terms were used in the English databases: “neoplasms”, “ carcinoma”, “ adenocarcinoma”, “cancer*”, “carcin*”, “neoplas*”, “tumo*”, “adenocarcinoma*”, “pancreas”, “pancreatic”, “complementary therapies”, “drugs, Chinese herbal”, “medicine, traditional”, “herbal medicine”, “medicine, east asian traditional”, “plant extracts”, “phytotherapy”, “alternative medicine”, “complementary therap*”, “chinese medicine”, “herb*”, “herbalism”, “plant extract*”, “medicinal plant*”, “oriental medicine”. Equivalent search words were used in the Chinese databases (the detailed search strategy is available in Supplement 1). We searched for additional trials by reviewing the reference lists of studies related to TMPs combined with chemotherapy for APC. All studies were independently searched by two reviewers (J. Hu and J. Jiang). Any disagreement arising from this process was resolved by consensus or by a third reviewer (M. Cheng).

### 2.4 Data Extraction

Two reviewers (G. Zhu and S. He) independently imported the studies into Endnote X9 software. After the exclusion of duplicate studies, the remaining studies were determined independently by two reviewers (H. Yu and B. Shi) by reading the title, abstract, and full text. Any disagreement arising during this process was discussed or decided by a third reviewer (J. Hu). Two reviewers (Y. Li and Z. Yao) imported the relevant data from the included studies into EpiData 3.1. The extracted data included the basic information (title, first author, year of publication, sample size ratio, sex ratio, age range, etc.), methods (blind methods, random methods, interventions, etc.), and outcomes. When relevant data were incomplete, we contacted the author or included an explanation in our article.

### 2.5 Assessment of Risk of Bias

Three reviewers (J. Hu, J. Jiang and X. Zhang) independently evaluated the included studies using the Cochrane risk of bias tool for RCTs according to the guidance of the Cochrane Handbook for Systematic Review of Interventions (version 5.1.0), which includes the following seven bias domains: selection bias due to random sequence generation, selection bias due to allocation concealment, performance bias due to blinding of participants and personnel, detection bias due to blinding of outcome assessment, attrition bias due to incomplete outcome data, reporting bias due to selective reporting, and other biases. The overall judgment on the risk of bias for each domain had three response options (low/high/unclear) (20). Any disagreement arising from this process was resolved by consensus or by a third reviewer (R. Liu).

### 2.6 Statistical Analysis

#### 2.6.1 Strategy for Data Synthesis

Two reviewers (J. Hu and J. Jiang) conducted a meta-analysis on the included studies using Review Manager 5.3. The risk ratio (RR) was used to present the dichotomous data, whereas the standardized mean difference (SMD) was used to present continuous data. The 95% confidence intervals (CIs) were defined, and statistical significance was set at p<0.05. Cochran’s Q test and the I² statistic were used to assess heterogeneity. The heterogeneity among different trials was described by the I² index, indicating a high statistical heterogeneity at > 50%. If heterogeneity (p ≥ 0.10, I² ≤ 50%) was rejected, a fixed-effects model (FEM) was used to synthesize the RR, SMD, and their 95% CI. Otherwise, a random-effects model (REM) was utilized. Sensitivity analysis was performed by sequentially excluding each trial to examine the robustness of the results. Publication bias was evaluated according to the non-parametric trim-and-fill analysis of publication bias and Egger’s test when there were more than 10 included studies.

#### 2.6.2 Analysis of Subgroups or Subsets

According to the KPS score, drug delivery of TMPs, the number of chemotherapy drug, chemotherapy regimen, follow-up time, and different herbs or combination of herbs, subgroup analysis was performed to reveal the clinical heterogeneity and its influence on outcomes.

### 2.7 Assessment of Evidence Quality

Two reviewers (M. Cheng and J. Hu) independently evaluated the quality of evidence for each outcome by GRADE profiler, which included the following five domains: risk of bias, inconsistency, indirectness, imprecision, and publication bias. The overall judgment on the quality of the evidence for each outcome had four response options (high/moderate/low/very low) (21). Any disagreement arising from this process was resolved by consensus or by a third reviewer (R. Liu).

## 3 Results

### 3.1 Literature Screening Results

**Figure 1:**
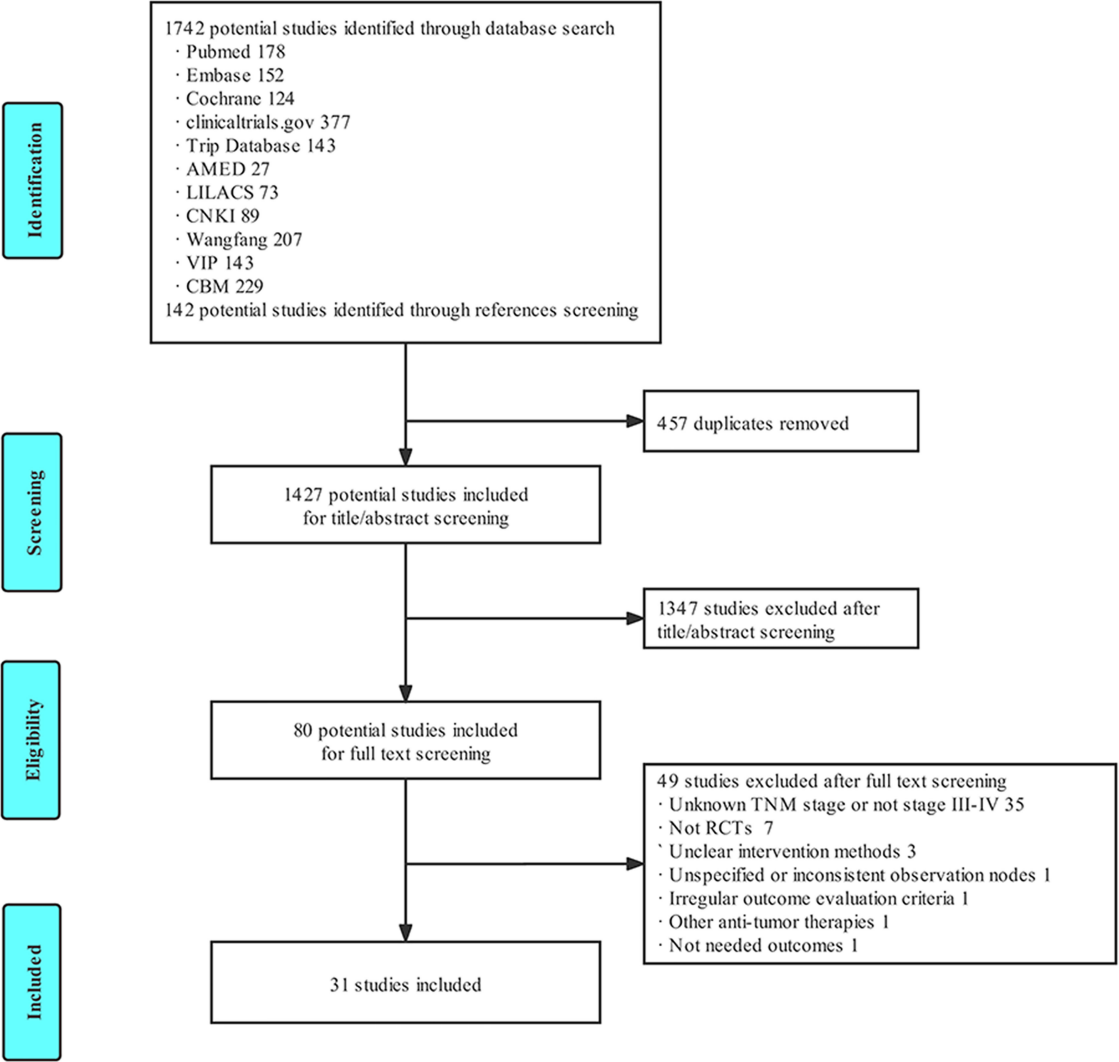
Flow diagram of literature search and study selection.

A total of 1,884 studies were obtained in the primary search and references screening, and 1,427 studies were included after the elimination of 457 duplicated studies. A total of 80 studies were selected after screening the titles, abstracts. Ultimately, 31 eligible studies were included in the final meta-analysis after reading the full text, according to the inclusion and exclusion criteria. The literature screening process is illustrated in **Figure 1**.

### 3.2 Study Characteristics

A total of 1,989 individuals (1,014 subjects in the experimental group and 975 subjects in the control group) with APC were included in 31 RCTs whose basic characteristics are listed in **Table 1**. Studies were conducted in China, America or Germany and published in Chinese or English between 2002 and 2021. 21 trials (22, 24–29, 31, 33–35, 37, 38, 40–45, 49, 51) included individuals with KPS score < 70, 4 trials (39, 46–48) included individuals with KPS score ≥70, and 6 trials (23, 30, 32, 36, 50, 52) included individuals with unclear KPS score. Of the different drug delivery, 18 trials (22–32, 35–38, 44, 45, 50) used oral TMPs, whereas 13 trials (33, 34, 39–43, 46–49, 51, 52) used intravenous TMPs. Regarding the chemotherapy regimens, 18 trials (22–25, 27, 31–35, 41, 43, 46, 47, 49–52) used single-drug chemotherapy, whereas 13 trials (26, 28–30, 36–40, 42, 44, 45, 48) used double-drugs chemotherapy. Furthermore, 21 trials (23, 24, 26, 28–30, 33, 34, 36–45, 48, 50, 52) used GEM-based chemotherapy, 12 trials (25, 27, 28, 30–32, 35, 36, 46–48, 51) used S-1-based chemotherapy, and 2 trials (22, 49) used others. The follow-up time for all trials was between 3 and 27 weeks. Moreover, 25 trials reported tumor response including ORR or DCR according to the WHO or RECIST guidelines, and 3 trials did not state evaluation criteria. 14 trials reported QoL according to the KPS, 5 trials reported the level of cancer biomarkers, and 14 trials reported ADRs according to the WHO or NCI chemotherapy toxicity response grading criteria.

**Table 1:** Main characteristics of studies included in the meta-analysis.

First Author and Publication Year	Advanced pancreatic cancer (APC)						TMPs/Quality Control	Interventions			Follow-up	Outcomes
	Country	KPS Score	E/C	M/F	TNM Stage	Age(E/C), mean or mean ± SD		Specific Components	Drug delivery	Chemotherapy Regimen		
Liu.H.2018 (22)	China	>60	30/29	37/22	III: 42, IV: 17	63.34 ± 9.47/62.20 ± 7.44	TMP (Decoction): 200ml, bid, d1-d21, 21d/C, 2Cycles/Unified production by the hospital	*BUPLEURI RADIX* 10g*, PAEONIAE RADIX ALBA* 20g*, CODONOPSISRADIX* 20g*, PORIA* 10g*, ATRACTYLODIS MACROCEPHALAE RHIZOMA* 10g*, CITRI RETICULATAE PERICARPIUM* 10g*, PINELLIAE RHIZOMA PRAEPARATUM* 10g*, GLYCYRRHIZAE RADIX ET RHIZOMA* 5g	Orally	CAP: 1250mg/m^2^, bid, d1-d14, 21d/C, 2Cysles	6w	O1,2,4
Huang.H.2021 (23)	China	Unclear	31/31	36/26	III: 33, IV: 29	51.39 ± 7.25/51.84 ± 7.02	TMP (Decoction): 100ml, tid, d1-d14, 14d/C, 4Cycles/Unclear	*ASTRAGALI RADIX* 40g*, SCUTELLARIAE BARBATAE HERBA* 30g*, HEDYOTIS DIFFUSA* 30g*, ANGELICAE SINENSIS RADIX* 20g*, COICIS SEMEN* 15g*, PORIA* 15g*, ATRACTYLODIS MACROCEPHALAE RHIZOMA* 12g*, SOLANUM NIGRUM* 12g*, SOLANUM LYRATUM THUNB* 12g*, PARIDIS RHIZOMA* 12g*, SPARGANII RHIZOMA* 9g*, CURCUMAE RHIZOMA* 9g	Orally	GEM: 1000mg/m^2^, qd, d1, d8, d15, 28d/C, 2Cysles	8w	O1,3
Dai.L.2014 (24)	China	>60	25/25	27/23	III, IV	55.2 ± 13.9/56.4 ± 14.8	TMP (Granules): 10g, bid, d1-d21,21d/C, 4Cycles/Unified production by the hospital	*HEDYOTIS DIFFUSA* 30g*, DRY TOAD SKIN* 6g, 2 *GECKOs, RHIZOMA AMORPHOPHALLI* 10g*, RHEI RADIX ET RHIZOMA* 6g*, GYNOSTEMMA PENTAPHYLLUM* 15g*, AMOMUM CARDAMOMUM* 10g	Orally	GEM: Unclear	12w	O1
Tong.X.2021 (25)	China	≥60	24/24	21/27	III: 13, IV: 35	62.5 ± 7.7/62.2 ± 8.0	TMP (Decoction): d1-d21, 21d/C, 3Cycles/Unclear	*CODONOPSISRADIX* 20g*, ASTRAGALI RADIX* 15g*, ATRACTYLODIS MACROCEPHALAE RHIZOMA* 15g*, PORIA* 15g*, GLYCYRRHIZAE RADIX ET RHIZOMA* 6g*, OPHIOPOGONIS RADIX* 12g*, COICIS SEMEN* 30g*, FRITILLARIAE THUNBERGII BULBUS* 15g*, LIGUSTRI LUCIDI FRUCTUS* 10g*, HEDYOTIS DIFFUSA* 30g*, CREMASTRAE PSEUDOBULBUS PLEIONES PSEUDOBULBUS* 10g*, RADIX ACTINIDIAE CHINENSIS* 20g*, CITRI RETICULATAE PERICARPIUM* 8g*, PINELLIAE RHIZOMA* 9g	Orally	S-1: BSA < 1.25m^2^: 40mg, bid; 1.25m^2^ < BSA < 1.5m^2^: 50mg, bid; 1.5m^2^ < BSA: 60mg, bid, d1-d14, 21d/C, 3Cycles	9w	O1,2,4
Bi.L.2017 (26)	China	≥60	20/20	22/18	III, IV	54.9 ± 6.9/54.3 ± 6.2	TMP (Decoction): 150ml, bid, d1-d28, 28d/C, 2Cycles/Unclear	*CODONOPSISRADIX* 25g*, ATRACTYLODIS MACROCEPHALAE RHIZOMA* 15g*, PORIA* 15g*, GLEHNIAE RADIX* 15g*, RADIX ACTINIDIAE CHINENSIS* 20g*, VESPAE NIDUS* 10g*, CRATAEGI FRUCTUS* 15g*, SHEN QU* 15g*, HORDEI FRUCTUS GERMINATUS* 15g*, EUONYMUS ALATUS* 10g	Orally	GEM+DDP: GEM: 1000mg/m^2^, qd, d1, d8, d15, 28d/C, 2Cysles; DDP: 30mg/m^2^, qd, d4-d6, 28d/C, 2Cysles	8w	O1
Yu.M.2020 (27)	China	>60	20/20	20/20	IV: 40	69.1 ± 6.9/69.5 ± 7.0	TMP (Decoction): d1-d21, 21d/C, 4Cycles/Unclear	*GINSENG RADIX ET RHIZOMA, CODONOPSISRADIX, ATRACTYLODIS MACROCEPHALAE RHIZOMA, ARTEMISIAE SCOPARIAE HERBA, GARDENIAE FRUCTUS, PHELLODENDRI CHINENSIS CORTEX, RHEI RADIX ET RHIZOMA, PERSICAE SEMEN, RADIX ACTINIDIAE CHINENSIS, VESPAE NIDUS, GLYCYRRHIZAE RADIX ET RHIZOMA PRAEPARATA CUM MELLE*	Orally	S-1: BSA < 1.25m^2^: 40mg, bid; 1.25m^2^ < BSA < 1.5m^2^: 50mg, bid; 1.5m^2^ < BSA: 60mg, bid, d1-d14, 21d/C, 4Cycles	12w	O1,2
Li.F.2021 (28)	China	>60	32/32	41/23	III: 17, IV: 47	59.5 ± 1.7/62.7 ± 1.9	TMP (Decoction): 300ml, bid, d1-d30, 28d/C, 3Cycles/Unclear	*PSEUDOSTELLARIAE RADIX* 15g*, ATRACTYLODIS MACROCEPHALAE RHIZOMA* 15g*, GANODERMA* 30g*, AKEBIA TRIFOLIATA KOIDZ* 15g*, TARAXACI HERBA* 30g*, PINELLIAE RHIZOMA PRAEPARATUM CUM ZINGIBERE ET ALUMINE* 9g*, COPTIDIS RHIZOMA* 3g	Orally	GEM+S-1: GEM: 1000mg/m^2^, qd, d1, d8, 21d/C, 2Cysles; S-1: 30mg/m^2^, bid, d1-d14, 21d/C, 2Cysles	12w	O1,2
Liu.E.2021 (29)	China	≥60	46/43	52/37	III: 66, IV: 23	46.49 ± 8.43/47.32 ± 9.12	TMP (Decoction): 150ml, bid, d1-d28, 28d/C, 2Cycles/Unclear	*ASTRAGALI RADIX* 30g*, ATRACTYLODIS MACROCEPHALAE RHIZOMA* 15g*, DIOSCOREAE RHIZOMA* 15g*, LYCII FRUCTUS* 15g*, LIGUSTRI LUCIDI FRUCTUS* 15g*, CITRI RETICULATAE PERICARPIUM VIRIDE* 9g*, SARGASSUM* 12g*, CREMASTRAE PSEUDOBULBUS PLEIONES PSEUDOBULBUS* 12g*, SOLANUM LYRATUM* 12g*, HEDYOTIS DIFFUSA* 12g*, SPARGANII RHIZOMA* 9g*, CURCUMAE RHIZOMA* 9g*, TRIONYCIS CARAPAX* 20g*, GLYCYRRHIZAE RADIX ET RHIZOMA* 6g	Orally	GEM+OXA: GEM: 1000mg/m^2^, qd, d1, d8, d15, 28d/C, 2Cysles; OXA: 100mg/m^2^, d1, 28d/C, 2Cysles	8w	O1,2,3
Chen.Z.2021 (30)	China	Unclear	60/60	67/53	III: 22, IV: 98	54.7 ± 2.4/54.5 ± 2.5	TMP (Decoction): 150ml, bid, d1-d21, 21d/C, 1Cycles/Unclear	*HEDYOTIS DIFFUSA* 50g*, ASTRAGALI RADIX PRAEPARATA CUM MELLE* 30g*, SARCANDRAE HERBA* 30g*, the leaves of Mangifera* 30g*, MASSA FERMENTATA* 25g*, ATRACTYLODIS MACROCEPHALAE RHIZOMA* 15g*, PORIA* 15g*, HORDEI FRUCTUS GERMINATUS* 15g*, SPARGANII RHIZOMA* 15g*, CURCUMAE RHIZOMA* 15g*, RHIZOMA AMORPHOPHALLI* 15g*, AUCKLANDIAE RADIX* 12g*, GINSENG RADIX ET RHIZOMA* 10g*, GLYCYRRHIZAE RADIX ET RHIZOMA PRAEPARATA CUM MELLE* 10g*, AMOMI FRUCTUS* 10g*, NOTOGINSENG RADIX ET RHIZOMA* 6g*, GEKKO SWINHONIS GUENTHER* 6g	Orally	GEM+S-1: GEM: 1000mg/m^2^, qd, d1, d8, 21d/C, 1Cysles; S-1: BSA < 1.25m^2^: 40mg, bid; 1.25m^2^ < BSA < 1.5m^2^: 50mg, bid; 1.5m^2^<BSA: 60mg, bid; d1-d14, 21d/C, 1Cycles	3w	O3
Chen.L.2020 (31)	China	>60	30/30	30/30	IV: 60	66.2 ± 7.0/66.1 ± 6.8	TMP (Decoction): 150ml, bid, d1-d21, 21d/C, 4Cycles/Unclear	*ARTEMISIAE SCOPARIAE HERBA* 25g*, GARDENIAE FRUCTUS* 10g*, RHEI RADIX ET RHIZOMA* 5g*, CODONOPSISRADIX* 20g*, ASTRAGALI RADIX* 20g*, ATRACTYLODIS MACROCEPHALAE RHIZOMA* 30g*, PORIA* 20g*, RADIX ACTINIDIAE CHINENSIS* 15g*, RHIZOMA AMORPHOPHALLI* 15g*, SMILACIS GLABRAE RHIZOMA* 15g*, GALLI GIGERII ENDOTHELIUM CORNEUM* 30g*, SEDI HERBA* 30g	Orally	S-1: BSA < 1.25m^2^: 40mg, bid; 1.25m^2^ < BSA < 1.5m^2^: 50mg, bid; 1.5m^2^ < BSA: 60mg, bid, d1-d14, 21d/C, 4Cycles	12w	O1,2
Bi.X.2020 (32)	China	Unclear	30/30	35/25	III: 33, IV: 27	64.2 ± 11.1/64.1 ± 10.7	TMP (Decoction): 150ml, bid, d1-d14, 14d/C, 4Cycles/Unclear	*BUPLEURI RADIX* 15g*, CYPERI RHIZOMA* 10g*, PINELLIAE RHIZOMA* 10g*, ACONITI LATERALIS RADIX PRAEPARATA* 10g*, GINSENG RADIX ET RHIZOMA* 30g*, SCUTELLARIAE RADIX* 10g*, ASTRAGALI RADIX* 25g*, ANGELICAE SINENSIS RADIX* 15g*, PERSICAE SEMEN* 15g*, CARTHAMI FLOS* 10g*, HIRUDO* 5g*, EUPOLYPHAGA STELEOPHAGA* 5g, 3 *SCOLOPENDRAs, GLYCYRRHIZAE RADIX ET RHIZOMA* 20g	Orally	S-1: BSA < 1.25m^2^: 40mg, bid; 1.25m^2^ < BSA < 1.5m^2^: 50mg, bid; 1.5m^2^ <BSA: 60mg, bid; d1-d28, 42d/C, 4Cycles	24w	O1
Z. Meng.2012 (33)	China	>60	39/37	46/30	Unresectable (locally advanced and/or metastatic)	60.2 ± 9.5/84.9 ± 6.5	TMP (Injection): 20ml/m^2^, 5 days a week for 3 weeks then 1 week off, 2Cycles/Detailed quality control and evaluation methods	*Huachansu*	Intravenously	GEM: 1000mg/m^2^, qd, d1, d8, d15, 28d/C, 2Cysles	8w	O1,4
Lee S Schwartzberg.2017 (34)	American	≥60	Cohort1:28/13	Cohort1:20/21	Cohort1: IIA: 1, III: 3, IV: 37	Cohort1: 45-84/41-81	Cohort1: TMP (Injection): 30g/day, d1-d5, d8-d12, d15-d19, 2Cycles/An approved drug and has a marketing authorisation in China	A neutral oil extracted and isolated from coix seed	Intravenously	GEM: 1000mg/m^2^, qd, d1, d8, d15, 28d/C, 2Cysles	8w	O1,4
			Cohort2:12/6	Cohort2:8/10	Cohort2: IIB: 1, III: 0, IV: 16, Unclear: 1	Cohort2: 48-82/52-77	Cohort2: TMP (Injection): 50g/day, d1-d5, d8-d12, d15-d19, 2Cycles/An approved drug and has a marketing authorisation in China					
			Cohort3:17/9	Cohort3:12/14	Cohort3: III: 2, IV: 24	Cohort3: 33-79/44-81	Cohort3: TMP (Injection): 30g/day, d1-d5, d8-d12, d15-d19, 2Cycles/An approved drug and has a marketing authorisation in China					
Liu.Q.2016 (35)	China	≥60	20/20	18/22	III: 19, IV: 21	55~75/49~74	TMP (Decoction): 200ml, bid, d1-d42, 42d/C, 2Cycles/Unclear	*BUPLEURI RADIX* 15g*, PAEONIAE RADIX ALBA* 15g*, CODONOPSISRADIX* 20g*, ATRACTYLODIS MACROCEPHALAE RHIZOMA* 15g*, PORIA* 15g*, PINELLIAE RHIZOMA PRAEPARATUM* 9g*, CITRI RETICULATAE PERICARPIUM* 6g*, GLYCYRRHIZAE RADIX ET RHIZOMA* 5g	Orally	S-1: BSA < 1.25m^2^: 40mg, bid; 1.25m^2^ < BSA < 1.5m^2^: 50mg, bid; 1.5m^2^ <BSA: 60mg, bid; d1-d28, 42d/C, 2Cycles	12w	O1,2,4
Luo.X.2015 (36)	China	Unclear	29/28	Unclear	III, IV	Unclear	TMP (Decoction): 150ml, bid, d1-d21, 21d/C, 2Cycles/Unclear	*PAEONIAE RADIX RUBRA, MOUTAN CORTEX, SCUTELLARIAE BARBATAE HERBA, POLYGONI CUSPIDATI RHIZOMA ET RADIX, TARAXACI HERBA, SCOLOPENDRA*	Orally	GEM+S-1: GEM: 1000mg/m^2^, qd, d1, d8, 21d/C, 2Cysles; S-1: 40mg/m^2^, bid, d1-d14, 21d/C, 2Cysles	6w	O1
You.J.2009 (37)	China	>55	20/20	23/17	III: 5, IV: 35	59. 70 ± 8. 21/60. 25 ± 8. 08	TMP (Oral liquor): 30ml, tid, d1-d28, 28d/C, 2Cycles/An approved drug and has a marketing authorisation in China	*CODONOPSISRADIX, POLYPORUS, ATRACTYLODIS MACROCEPHALAE RHIZOMA, SETARIAE FRUCTUS GERMINATUS, HORDEI FRUCTUS GERMINATUS, PORIA, PORIA WITH HOSTWOOD, COICIS SEMEN, PINELLIAE RHIZOMA, CITRI RETICULATAE PERICARPIUM, ERIOBOTRYAE FOLIUM, GLYCYRRHIZAE RADIX ET RHIZOMA PRAEPARATA CUM MELLE*	Orally	GEM+OXA: GEM: 1000mg/m^2^, qd, d1, d8, 28d/C, 2Cysles; OXA: 100mg/m^2^, qd, d1, 28d/C, 2Cysles	8w	O1,2,3
Wei.D.2006 (38)	China	≥50	21/21	29/13	III: 32, IV: 10	60.5 ± 6/59.5 ± 8	TMP (Decoction): 20g, bid, 28/C, 3Cycles/Unclear	*ASINI CORII COLLA* 20g	Orally	GEM+OXA: GEM: 1000mg/m^2^, qd, d1, d8, d15, 28d/C, 3Cysles; 5-Fu: 600mg/m^2^, qd, d1-d5, 28d/C, 3Cysles	12w	O1
Sun.Y.2016 (39)	China	>70	35/35	Unclear	III, IV	Unclear	TMP (Injection): 20ml, qd, d1-d14, 21/C, >3Cycles/An approved drug and has a marketing authorisation in China	*SOPHORAE FLAVESCENTIS RADIX, RHIZOMA HETEROSMILACIS JAPONICAE*	Intravenously	GEM+DDP: GEM: 1000mg/m^2^, qd, d1, d8, 21d/C, >3Cysles; DDP: 25mg/m^2^, qd, d1-d3, 21d/C, >3Cysles	>9w	O1,2,4
Dou.L.2010 (40)	China	≥60	26/26	31/21	III: 45, IV: 7	42-73/43-74	TMP (Injection): 50ml, qd, d1-d28, 28/C, 2Cycles/An approved drug and has a marketing authorisation in China	*ASTRAGALI RADIX, GINSENG RADIX ET RHIZOMA, SOPHORAE FLAVESCENTIS RADIX*	Intravenously	GEM+DDP: GEM: 1000mg/m^2^, qd, d1, d8, d15, 28d/C, 2Cysles; DDP: 30mg/m^2^, qd, d4-d6, 28d/C, 2Cysles	8w	O1,4
Guo.Z.2011 (41)	China	≥60	50/50	59/41	III: 81, IV: 19	40-73/41-72	TMP (Injection): 30ml, bid, d1-d28, 28/C, 2Cycles/An approved drug and has a marketing authorisation in China	*ASTRAGALI RADIX, GINSENG RADIX ET RHIZOMA, SOPHORAE FLAVESCENTIS RADIX*	Intravenously	GEM: 1000mg/m^2^, qd, d1, d8, d15, 28d/C, 2Cysles	8w	O1,4
Dou.L.2012 (42)	China	≥60	28/28	33/23	III: 49, IV: 7	60 ± 9/61 ± 8.5	TMP (Injection): 50ml, qd, d1-d14, 21/C, 2Cycles/An approved drug and has a marketing authorisation in China	*ASTRAGALI RADIX, GINSENG RADIX ET RHIZOMA, SOPHORAE FLAVESCENTIS RADIX*	Intravenously	GEM+CAP: GEM: 1000mg/m^2^, qd, d1, d8, 21d/C, 2Cysles; CAP: 1250mg/m^2^, bid, d1-d14, 21d/C, 2Cysles	6w	O1,4
Niu.S.2014 (43)	China	>60	40/40	43/37	IV: 80	Unclear	TMP (Injection): 20ml, qd, d1-d20, 28/C, 2Cycles/An approved drug and has a marketing authorisation in China	*Huachansu*	Intravenously	GEM: 1000mg/m^2^, qd, d1, d8, d15, 28d/C, 2Cysles	8w	O1,2
Hu.B.2010 (44)	China	>60	30/30	33/27	III: 14, IV: 46	58.62 ± 7.32/59.28 ± 7.46	TMP (Decoction):167ml, tid, d1-d28, 28d/C, 2Cycles/Unclear	*GINSENG RADIX ET RHIZOMA* 5g*, ASTRAGALI RADIX* 30g*, AURANTII FRUCTUS* 10g*, CHUANXIONG RHIZOMA*15g*, PHERETIMA* 10g*, BUPLEURI RADIX* 8g*, SCOLOPENDRA* 3g*, CURCUMAE RHIZOMA* 15g*, SOLANUM NIGRUM* 15g*, GLYCYRRHIZAE RADIX ET RHIZOMA PRAEPARATA CUM MELLE* 6g	Orally	GEM+OXA: GEM: 1000mg/m^2^, qd, d1, d8, 28d/C, 2Cysles; OXA: 100mg/m^2^, qd, d1, 28d/C, 2Cysles	8w	O3
Chen.X.2005 (45)	China	≥60	41/40	55/36	III: 47, IV: 34	Median age:55/54	TMP (Pills): 10 pills, tid, d1-d28, 28/C, 2Cycles/An approved drug and has a marketing authorisation in China	*SALVIAE MILTIORRHIZAE RADIX ET RHIZOMA, NOTOGINSENG RADIX ET RHIZOMA, BORNEOLUM SYNTHETICUM*	Orally	GEM+DDP: GEM: 1000mg/m^2^, qd, d1, d8, 28d/C, 2Cysles; DDP: 30mg/m^2^, qd, d4-d6, 28d/C, 2Cysles	8w	O1,2
Li.L.2016 (46)	China	≥70	27/26	31/22	IV: 53	56-76/59-82	TMP (Injection): 200ml, qd, d1-d28, 42/C, 2Cycles/An approved drug and has a marketing authorisation in China	A neutral oil extracted and isolated from coix seed	Intravenously	S-1: BSA < 1.25m^2^: 40mg, bid; 1.25m^2^ < BSA < 1.5m^2^: 50mg, bid; 1.5m^2^ < BSA: 60mg, bid; d1-d28, 42d/C, 2Cycles	12w	O1,4
Yao.X.2015 (47)	China	≥70	22/21	22/21	IV: 43	70.8-89.6/70.8-89.8	TMP (Injection): 200ml, qd, d1-d28, 42/C, 2Cycles/An approved drug and has a marketing authorisation in China	A neutral oil extracted and isolated from coix seed	Intravenously	S-1: 40-60mg/d, bid, d1-d28, 42d/C, 2Cycles	12w	O1,2,4
Zhang.X.2018 (48)	China	≥70	22/23	24/21	IV: 45	58.43 ± 12. 43/56.95 ± 10. 75	TMP (Injection): 200ml, qd, d1-d14, 21/C, 4-9Cycles/An approved drug and has a marketing authorisation in China	A neutral oil extracted and isolated from coix seed	Intravenously	GEM+S-1: GEM: 1000mg/m^2^, qd, d1, d8, 21d/C, 4-8Cysles; S-1: 1.25m^2^ ≤ BSA < 1.5m^2^: 40mg, bid; 1.5m^2^ ≤ BSA: 50mg, bid; d1-d14, 21d/C, 4-8Cycles	12-27w	O1
He.R.2015 (49)	China	≥60	80/80	94/66	III: 122, IV: 38	Unclear	TMP (Injection): 60ml, qd, d1-d21, 28/C, 3Cycles/An approved drug and has a marketing authorisation in China	*MYLABRIS, GINSENG RADIX ET RHIZOMA, ASTRAGALI RADIX, ACANTHOPANACIS SENTICOSI RADIX ET RHIZOMA SEU CAULIS*	Intravenously	DTX: 75mg/m^2^, qd, d1, 21d/C, 4Cysles	12w	O1,2,4
Gansauge F.2002 (50)	Germany	Unclear	28/28	41/19	III: 2, IV: 58	≥18	TMP (Pill): 20mg, weekly; first Cycle: 7 weeks of therapy, 1 week of rest, TMP was administered during the first 5 days in the first week; 2nd Cycle: 3 weeks of therapy, 1 week of rest/Being provided by Nowicky Pharma (Vienna, Austria)	A semisynthetic compound of thiotepa and the alkaloid chelidonine from the plant *Chelidonium majus*	Orally	GEM: 1000mg/m^2^, weekly; first Cycle: 7 weeks of therapy, 1 week of rest; 2nd Cycle: 1 weeks of therapy, 1 week of rest	12w	O1
Guan.L.2015 (51)	China	>60	27/27	27/27	IV:54	34-75	TCM (Injection): 50ml, qd, d1-d14, 42/C, 2Cycle/An approved drug and has a marketing authorisation in China	Sodium Cantharidinate	Intravenously	S-1: body surface area < 1.25m^2^: 40mg, bid; 1.25m^2^ < body surface area < 1.5m^2^: 50mg, bid; 1.5m^2^ < body surface area: 60mg, bid; d1-d28, 42d/C, 2Cycles	12w	O1,2,6
Deng.L.2013 (52)	China	Unclear	24/23	30/17	IV:54	65-80	TMP (Injection): 200ml, qd, d1-d14, 21/C, 6Cycles/An approved drug and has a marketing authorisation in China	A neutral oil extracted and isolated from coix seed	Intravenously	GEM: 800mg/m^2,^ qd, d1, d8, 21d/C, 6Cysles	18w	O1,6

SD, standard deviation; KPS, Karnofsky Performance Status; TMPs, traditional medicine preparations; E/C, experimental group (TMPs combined with chemotherapy)/control group (chemotherapy alone); M/F, male/female; BSA, body surface area; O, outcome; O1, tumor response; O2, quality of life (QOL); O3, the cancer biomarkers; O4, adverse drug reactions (ADRs).

### 3.3 Assessment of Methodological Bias Risk

**Figure 2:**
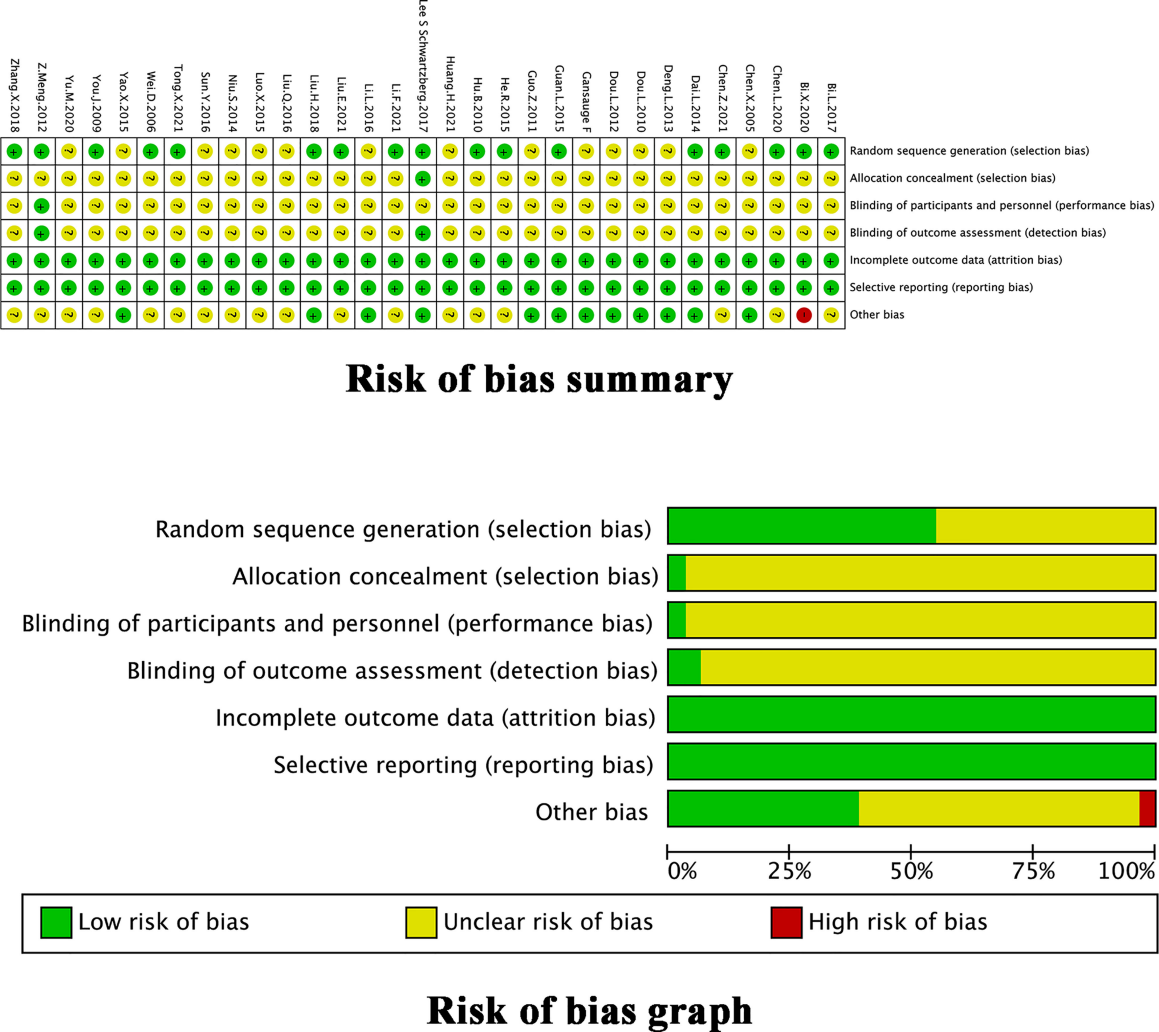
Assessment of methodological bias risk.

The assessment of the methodological bias risk of each trial included is shown in **Figure 2**. Only 17 trials (22, 24–26, 28–34, 37, 38, 44, 48, 49, 51) reportedly used a random sequence generation including random number table, envelope, bayesian algorithm and centralized interactive voice response system. Unclear selection bias existed because 14 trials did not describe random sequence generation. Just 1 trial (34) reported allocation concealment. Except for 2 trials (33, 34), studies failed to report the blinding method, which led to unclear performance and detection biases. None of the trials reported any loss to follow-up. All trials had low risk on attrition and reporting bias. The ORR and DCR evaluation criteria in 1 trial (32) did not coincide with our study which might influence results. Some unclear information, including KPS score, gender, age at the time of inclusion, and evaluation criteria of outcomes, in 10 trials (23, 30, 32, 33, 36, 37, 39, 43, 44, 48, 49) might lead to other potential bias. The quality of the TMPs was shown in **Table 1**, 14 trials (34, 37, 39–43, 45–52) described an approved TMPs having clear manufacturer, production batch number and marketing authorisation in China. 1 trial (33) described in detail the product quality control was assured and monitored by acquiring raw material from designated source provinces, establishing fingerprinting, measuring the concentrations of certain compounds in the extract, and comparing the high performance liquid chromatography fingerprinting. 1 trial (50) only described the provider of TMPs. The rest 15 trials (22–32, 35, 36, 38, 44) used self-prepared herbal decoctions prescribed by practitioners but did not describe the origin, processing method or dosage of herbs and none of them described a quality control method.

### 3.4 Tumor Response

**Figure 3:**
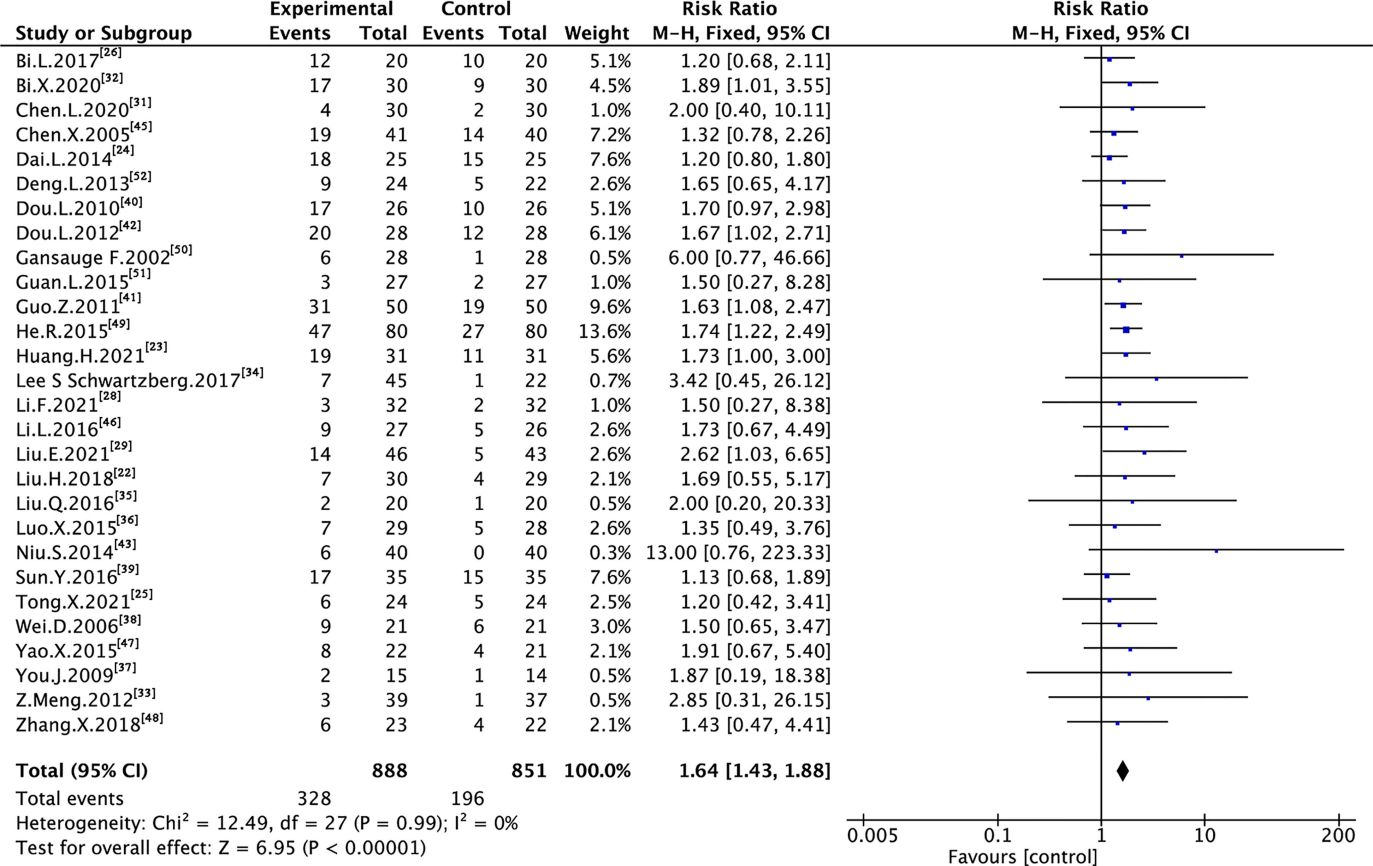
Meta-analysis results of ORR between the two groups.

**Figure 4:**
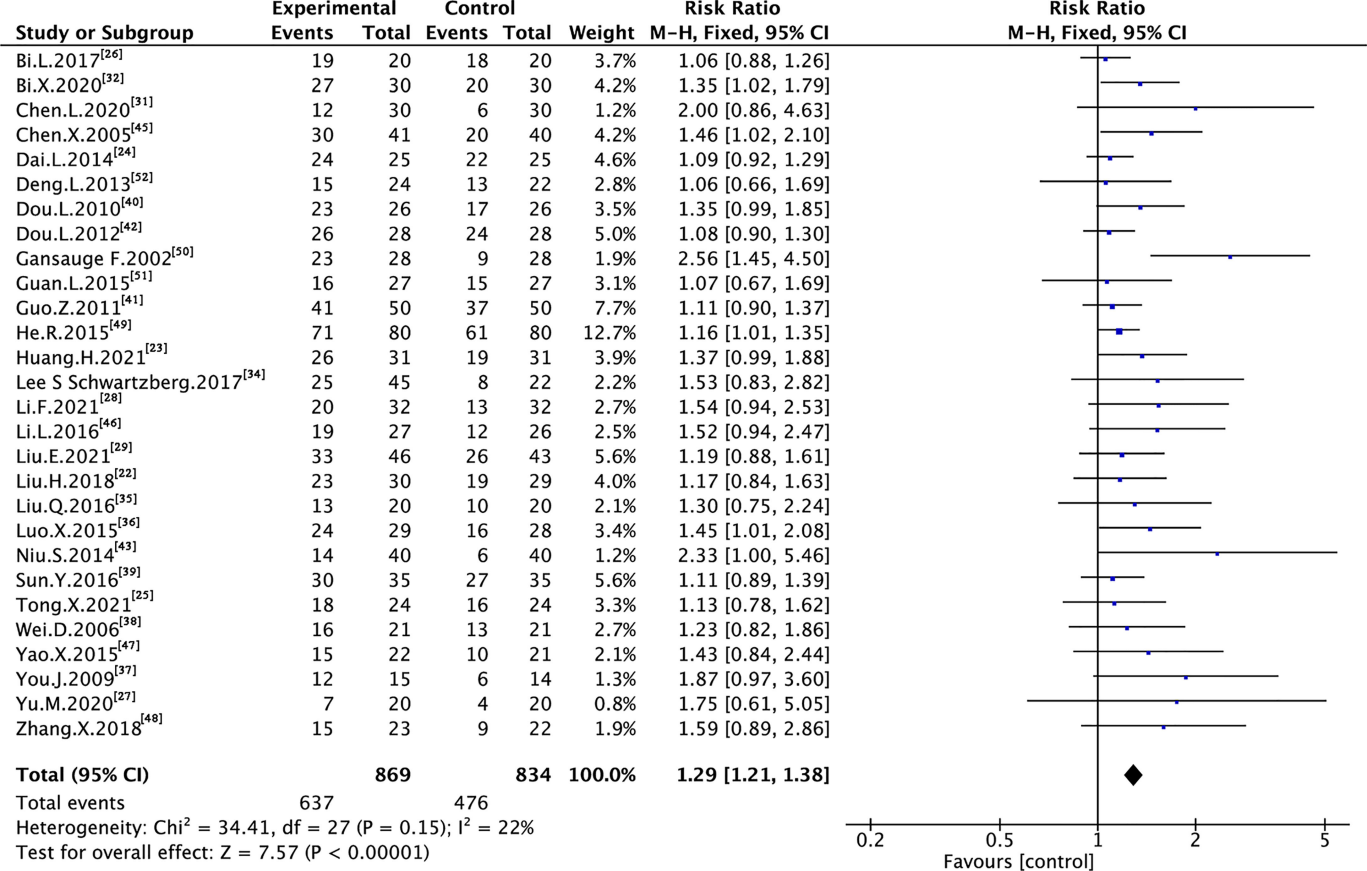
Meta-analysis results of DCR between the two groups.

A total of 29 trials assessing 1,739 and 1,703 cases reported ORR and DCR, respectively (**Figure 3** and **Figure 4**). As shown in the figures, there was low heterogeneity between trials as per Cochran’s Q test and Higgins’s I² (I² = 0%, I² = 22%); therefore, the FEM was used to synthesize data from different trials. The results of the meta-analysis showed that TMPs combined with chemotherapy increased ORR (RR=1.64, 95% CI [1.43 to 1.88], p <0.00001) and DCR (RR=1.29, 95% CI [1.21 to 1.38], p <0.00001), compared to chemotherapy alone.

### 3.5 Quality of Life

**Figure 5:**
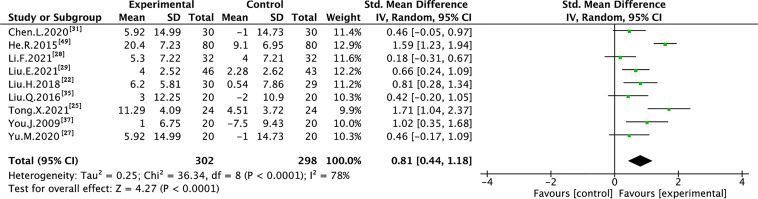
Meta-analysis results of QoL (continuous data) between the two groups.

**Figure 6:**
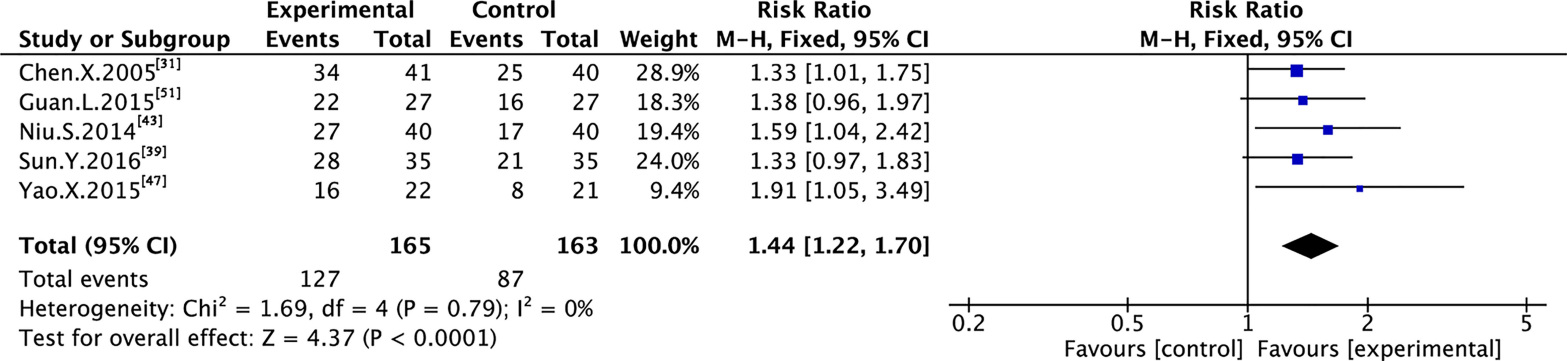
Meta-analysis results of QoL (dichotomous data) between the two groups.

Nine trials with 600 individuals reported the QoL by using continuous data (**Figure 5**), whereas four trials with 274 individuals reported it using dichotomous data (**Figure 6**) according to the KPS scale.

In the continuous data, high heterogeneity was observed in QoL (I² = 78%); therefore, REM was used to synthesize data from different trials. The results of the meta-analysis showed that TMPs combined with chemotherapy increased QoL (SMD=0.81, 95% CI [0.44 to 1.18], p <0.0001), compared to chemotherapy alone. To demonstrate the reason for the statistical heterogeneity of the results, subgroup analysis was performed (**Table S1** and **Figures S1–S5**). The drug delivery of TMPs might be the reason for the heterogeneity in QoL (**Figure S2**).

In the dichotomous data, no heterogeneity was observed in QoL (I² = 0%); therefore, FEM was used to synthesize data from different trials. The results of the meta-analysis showed that TMPs combined with chemotherapy increased QoL (RR=1.44, 95% CI [1.22 to 1.70], p<0.0001), compared to chemotherapy alone.

### 3.6 Cancer Biomarkers

**Figure 7:**
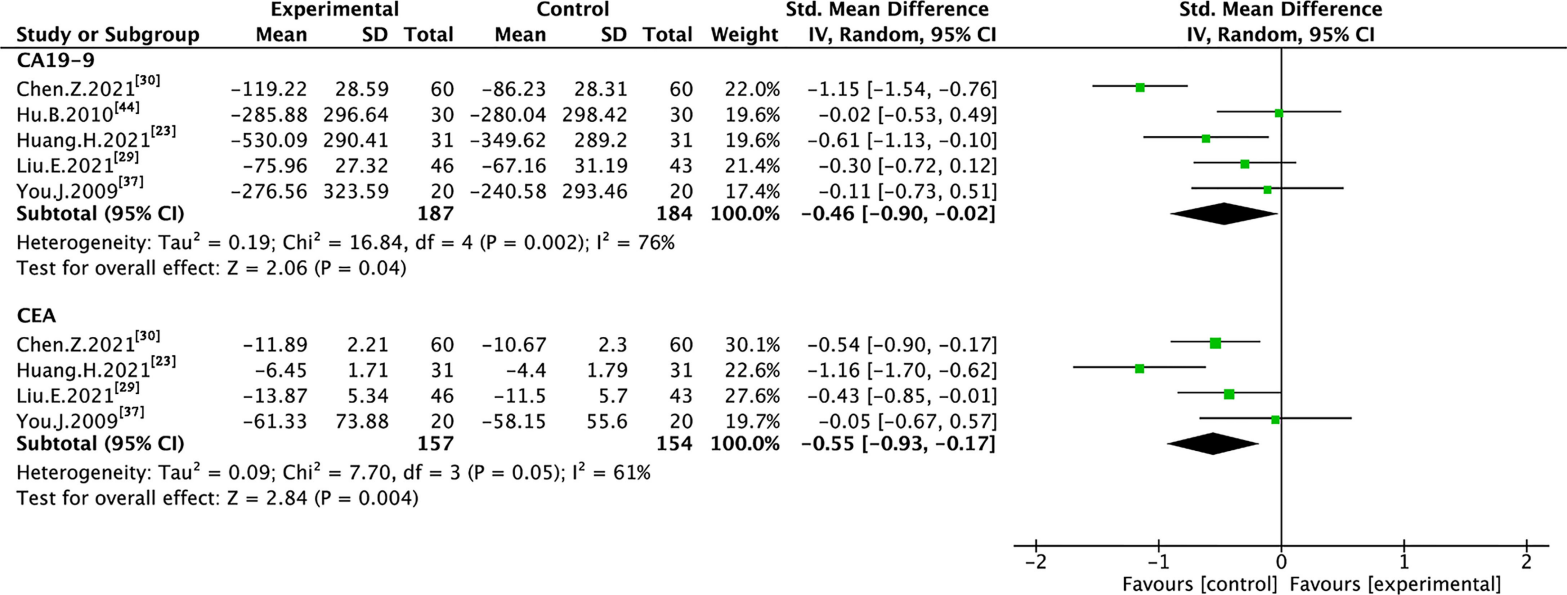
Meta-analysis results of cancer biomarkers between the two groups.

Five trials with 371 individuals reported cancer biomarkers (**Figure 7**). Statistical heterogeneity was demonstrated in CA19-9 (I² = 76%), and CEA (I² = 61%); therefore, REM was used to synthesize SMD. The results of the meta-analysis showed that TMPs combined with chemotherapy reduced the level of CA19-9 (SMD=-0.46, 95% CI [-0.90 to -0.02], *p*=0.04), and CEA (SMD=-0.55, 95% CI [-0.93 to -0.17], *p*=0.004), compared to chemotherapy alone. To demonstrate the reason for the statistical heterogeneity of the results, a subgroup analysis was performed (**Tables S2, S3** and **Figures S6–S9**). The follow-up time might be the reason for the heterogeneity in CA19-9 (**Figure S7**) and the number of chemotherapy drug might be the reason for the heterogeneity in CEA (**Figure S8**).

### 3.7 Adverse Drug Reactions

**Figure 8:**
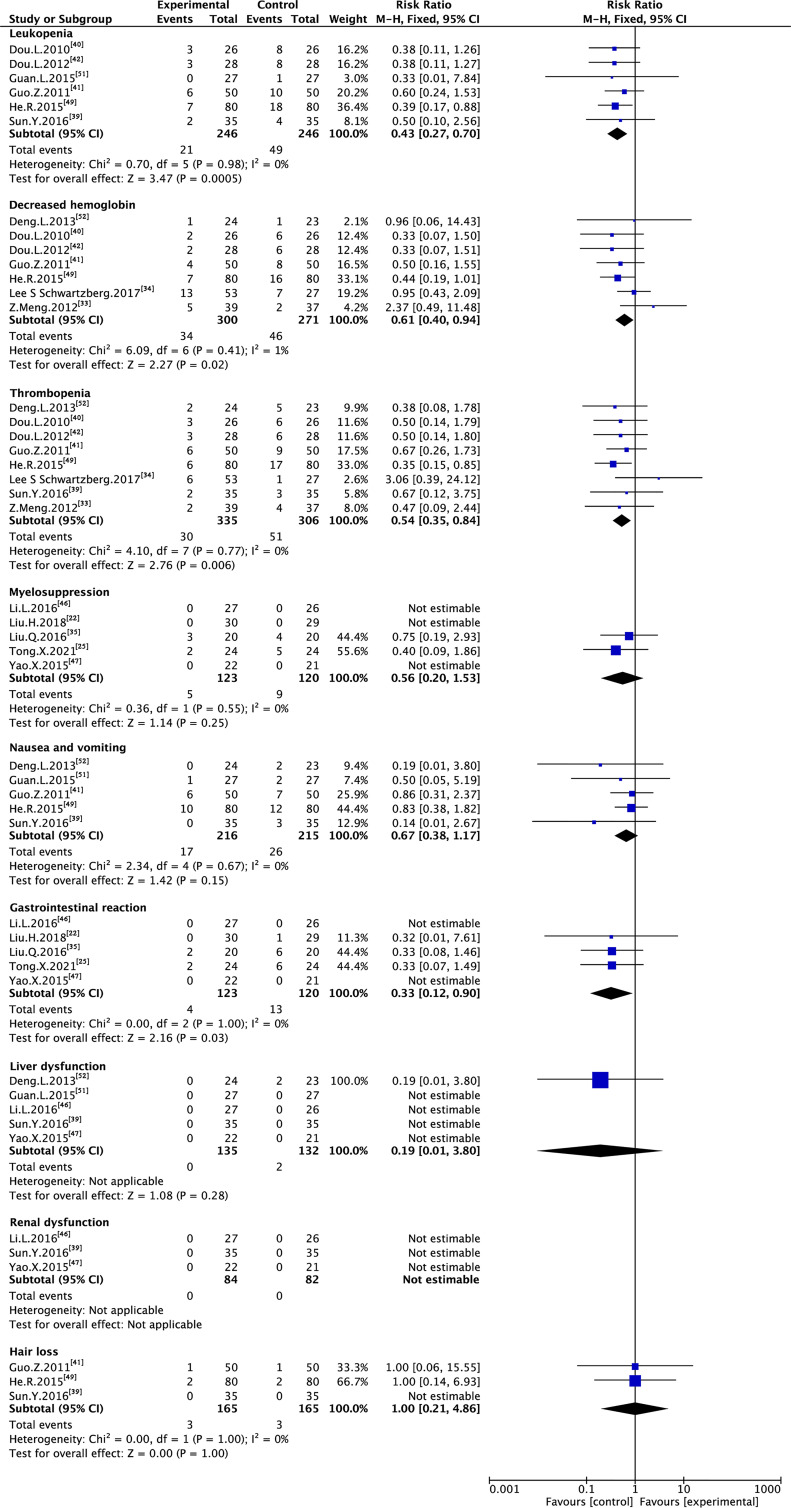
Meta-analysis results of ADRs between the two groups.

Six trials with 492 individuals reported leukopenia, seven trials with 571 individuals reported decreased hemoglobin, eight trials with 641 individuals reported thrombopenia, five trials with 243 individuals reported myelosuppression, five trials with 431 individuals reported nausea and vomiting, five trials with 243 individuals reported gastrointestinal reaction, five trials with 220 individuals reported liver dysfunction, three trials with 166 individuals reported renal dysfunction, and three trials with 330 individuals reported hair loss (**Table 2** and **Figure 8**).

**Table 2:** Meta-analysis results of adverse drug reactions.

Outcomes	Number of trials	Experimental group (Events/Total)	Control group (Events/Total)	SM	RR, 95% CI	Z	*p*	Heterogeneity	
								I²	*P_h_*
Leukopenia	6	21/246	49/246	FEM	0.43 [0.27, 0.70]	3.47	0.0005	0%	0.98
Decreased hemoglobin	7	34/300	46/271	FEM	0.61 [0.40, 0.94]	2.27	0.02	1%	0.41
Thrombopenia	8	30/335	51/306	FEM	0.54 [0.35, 0.84]	2.76	0.006	0%	0.77
Myelosuppression	5	5/123	9/120	FEM	0.56 [0.20, 1.53]	1.14	0.025	0%	0.55
Nausea and vomiting	5	17/216	26/215	FEM	0.67 [0.38, 1.17]	1.42	0.15	0%	0.67
Gastrointestinal reaction	5	4/123	13/120	FEM	0.33 [0.12, 0.90]	2.16	0.03	0%	1.00
Liver dysfunction	5	0/135	2/132	FEM	0.19 [0.01, 3.80]	1.08	0.28	Not applicable	Not applicable
Renal dysfunction	3	0/84	0/82	FEM	Not estimable	Not applicable	Not applicable	Not applicable	Not applicable
Hair loss	3	3/165	3/165	FEM	1.00 [0.21, 4.86]	0	1.00	0%	1.00

RR, risk ratio; CI, confidence interval; SM, statistical method; FEM, fixed-effects model.

Minimal heterogeneity was observed in decreased hemoglobin (I² = 1%), whereas no heterogeneity (I² = 0%) was observed in others. FEM was used to synthesize data from different trials. The results of the meta-analysis showed that TMPs combined with chemotherapy reduced the risk of leukopenia (RR=0.43, 95% CI [0.27-0.70], p =0.0005), decreased hemoglobin (RR=0.61, 95% CI [0.40-0.94], p =0.02), thrombopenia (RR=0.54, 95% CI [0.35-0.84], p =0.006), and gastrointestinal reaction (RR=0.33, 95% CI [0.12-0.90], p =0.03), compared to chemotherapy alone. However, there was no difference between two groups in myelosuppression (RR=0.56, 95% CI [0.20-1.53], p =0.25), nausea and vomiting (RR=0.67, 95% CI [0.38-1.17], p =0.15), liver dysfunction (RR=0.19, 95% CI [0.01-3.80], p =0.28) and hair loss (RR=1.00, 95% CI [0.21-4.86], p =1.00). Besides leukopenia, decreased hemoglobin and thrombopenia were common ADRs during treatment while kidney dysfunction did not occur in either group (**Table 3**).

**Table 3:** The incidence of different ADRs.

Outcomes	Overall Incidence	Experimental group		Control group	
		Events/Total	Incidence	Events/Total	Incidence
Leukopenia	14.23%	21/246	8.54%	49/246	19.92%
Decreased hemoglobin	14.01%	34/300	11.33%	46/271	16.97%
Thrombopenia	12.64%	30/335	8.96%	51/306	16.67%
Myelosuppression	5.76%	5/123	4.07%	9/120	7.50%
Nausea and vomiting	9.98%	17/216	7.870%	26/215	12.93%
Gastrointestinal reaction	7.00%	4/123	3.25%	13/120	10.83%
Liver dysfunction	0.75%	0/135	0.00%	2/132	1.52%
Renal dysfunction	0.00%	0/84	0.00%	0/82	0.00%
Hair loss	1.82%	3/165	1.82%	3/165	1.82%

### 3.8 Subgroup Analysis of ORR and DCR

Subgroup analysis was performed on ORR and DCR according to the KPS score, drug delivery of TMPs, the number of chemotherapy drug, chemotherapy regimen, and follow-up time (**Tables 4**, **5**, and **Figures S10–S19**). The KPS score was divided into three parts: <70, ≥70, and unclear. Subgroup analysis showed that TMPs increased ORR when KPS score <70 and unclear and DCR in every part (**Figures S10**, **S15**). The drug delivery of TMPs was either intravenously or orally. Subgroup analysis showed that TMPs increased ORR and DCR regardless of whether it was administered intravenously or orally (**Figures S11**, **S16**). Based on the number of chemotherapy drug, individuals were divided into those who used single-drug and those who used double-drugs. Subgroup analysis showed that TMPs increased ORR and DCR regardless of whether the number of chemotherapy drug used (**Figures S12**, **S17**). The chemotherapy regimen was divided into three categories: GEM-based, S-1-based, and other chemotherapy regimens. Subgroup analysis showed that TMPs increased ORR and DCR regardless of the above chemotherapy regimen used (**Figures S13**, **S18**). The follow-up time was divided into two parts: 6w≤ and <9w, and ≥9w. Subgroup analysis showed that TMPs increased ORR and DCR in every part of the follow-up time (**Figures S14**, **S19**).

**Table 4:** Subgroup analysis of the ORR.

Subgroups	Number of trials	RR (95% CI)	Z	*p*	Heterogeneity	
					I²	*P_h_*
**Table 4a. Subgroups analysis according to KPS score (** **Figure S10** **)**						
KPS score (<70)	19	1.64 [1.39, 1.93]	5.93	<0.00001	0%	0.97
KPS score (≥70)	4	1.40 [0.94, 2.07]	1.67	0.10	0%	0.76
Unclear	5	1.84 [1.29, 2.61]	3.37	0.0007	0%	0.79
**Table 4b. Subgroups analysis according to drug delivery of TMPs (** **Figure S11** **)**						
Intravenously	13	1.69 [1.40, 2.04]	5.52	<0.00001	0%	0.95
Orally	15	1.57 [1.28, 1.94]	4.27	<0.0001	0%	0.94
**Table 4c. Subgroups analysis according to the number of chemotherapy drug (** **Figure S12** **)**						
Single - drug	17	1.76 [1.46, 2.11]	6.04	<0.00001	0%	0.94
Double - drugs	11	1.48 [1.19, 1.83]	3.57	0.0004	0%	0.96
**Table 4d. Subgroups analysis according to chemotherapy regimen (** **Figure S13** **)**						
GEM-based chemotherapy regimen	19	1.60 [1.36, 1.88]	5.61	<0.00001	0%	0.89
S-1-based chemotherapy regimen	10	1.64 [1.16, 2.31]	2.79	0.005	0%	1.00
Others	2	1.73 [1.23, 2.44]	3.14	0.002	0%	0.96
**Table 4e. Subgroups analysis according to follow-up time (** **Figure S14** **)**						
6≤ and <9w	13	1.71 [1.40, 2.08]	5.27	<0.00001	0%	0.91
≥9w	15	1.58 [1.30, 1.92]	4.58	<0.00001	0%	0.96

RR, risk ratio; CI, confidence interval; ORR, objective response rate.

**Table 5:** Subgroup analysis of the DCR.

Subgroups	Number of trials	RR (95% CI)	Z	*p*	Heterogeneity	
					I²	*P_h_*
**Table 5a. Subgroups analysis according to KPS score (** **Figure S15** **)**						
KPS score (<70)	18	1.25 [1.15, 1.35]	5.52	<0.00001	6%	0.39
KPS score (≥70)	4	1.33 [1.09, 1.62]	2.79	0.005	6%	0.36
Unclear	6	1.48 [1.25, 1.75]	4.53	<0.00001	21%	0.28
**Table 5b. Subgroups analysis according to drug delivery of TMPs (** **Figure S16** **)**						
Intravenously	12	1.23 [1.12, 1.35]	4.46	<0.00001	0%	0.54
Orally	16	1.36 [1.23, 1.50]	6.18	<0.00001	39%	0.06
**Table 5c. Subgroups analysis according to the number of chemotherapy drug (** **Figure S17** **)**						
Single - drug	17	1.30 [1.19, 1.42]	5.86	<0.00001	24%	0.18
Double - drugs	11	1.29 [1.16, 1.42]	4.80	<0.00001	25%	0.21
**Table 5d. Subgroups analysis according to chemotherapy regimen (** **Figure S18** **)**						
GEM-based chemotherapy regimen	18	1.31 [1.21, 1.43]	6.39	<0.00001	45%	0.02
S-1-based chemotherapy regimen	11	1.40 [1.21, 1.62]	4.55	<0.00001	0%	0.94
Others	2	1.17 [1.02, 1.33]	2.21	0.03	0%	0.98
**Table 5e. Subgroups analysis according to follow-up time (** **Figure S19** **)**						
6≤ and <9w	12	1.29 [1.17, 1.42]	5.12	<0.00001	29%	0.16
≥9w	16	1.30 [1.18, 1.42]	5.58	<0.00001	20%	0.22

RR, risk ratio; CI, confidence interval; DCR, disease control rate.

TMPs are different combinations of multiple herbs. To determine which herbs or combination of herbs combined with chemotherapy contributed the most to APC, subgroup analysis was conducted based on the specific ingredients of TMPs from each study listed in **Table 1** according to the method described in Chen MH, et al. (53) and Chen Y et al. (54). All significant RR results were shown in **Tables 6A** and **6B**, and only the RRs with low heterogeneity (I^2^ < 30%) that were not greater than the total pooled RR were shown in the text. A total of 82 herbs were involved in the included trials, and the more frequently used herbs in treating APC were: *ATRACTYLODIS MACROCEPHALAE RHIZOMA* (bai zhu), *ASTRAGALI RADIX* (huang qi), *GLYCYRRHIZAE RADIX ET RHIZOMA* (gan cao), *PORIA* (fu ling), *GINSENG RADIX ET RHIZOMA* (ren shen), *CODONOPSIS RADIX* (dang shen), and *PINELLIAE R*HIZOMA (ban xia). As shown in **Table 6A**, six herbs had significant RRs with low heterogeneity in benefit for ORR. These single herbs were paired with each other and 15 pairs were generated. Only one herb pair named *ATRACTYLODIS MACROCEPHALAE RHIZOMA* + *PORIA* (n=7) (RR 1.66 [1.20, 2.28], I^2^ = 0%) had lower RR when compared with the total pool RR. As shown in **Table 6B**, nineteen herbs had significant RRs with low heterogeneity in benefit for ORR. These single herbs were paired with each other and forty-five pairs had lower RRs when compared with the total pool RR. The most frequent combinations were: *ATRACTYLODIS MACROCEPHALAE RHIZOMA* + *PORIAATRACTYLODIS* (n=7) (RR 1.29 [1.11, 1.51], I^2^ = 28%), and *MACROCEPHALAE RHIZOMA* + *GLYCYRRHIZAE RADIX ET RHIZOMA* (n=6) (RR 1.26 [1.06, 1.57], I^2^ = 0%). The combination of *GINSENG RADIX ET RHIZOMA* + *SOPHORAE FLAVESCENTIS RADIX* (n = 3) had the lowest RR (1.15 [1.01, 1.32], I^2^ = 0%). Compared with the total pool RR, 43 combinations of three plants presented lower RRs. The most frequent combinations were: *ATRACTYLODIS MACROCEPHALAE RHIZOMA* + *GLYCYRRHIZAE RADIX ET RHIZOMA* + *PORIA* (n=4) (RR 1.27 [1.02, 1.57], I^2^ = 0%), *ATRACTYLODIS MACROCEPHALAE RHIZOMA* + *GLYCYRRHIZAE RADIX ET RHIZOMA* + *PINELLIAE RHIZOMA* (n=4) (RR 1.27 [1.02, 1.57], I^2^ = 0%), *ATRACTYLODIS MACROCEPHALAE RHIZOMA + PORIA + PINELLIAE RHIZOMA* (n=4) (RR 1.27 [1.02, 1.57], I2 = 0%), and *GLYCYRRHIZAE RADIX ET RHIZOMA* + *PORIA* + *CODONOPSIS RADIX* (n=4) (RR 1.27 [1.02, 1.57], I^2^ = 0%). The combination of *ASTRAGALI RADIX + GINSENG RADIX ET RHIZOMA + SOPHORAE FLAVESCENTIS RADIX* (n = 3) had the lowest RR (1.15 [1.01, 1.32], I^2^ = 0%). Compared with the total pool RR, 23 combinations of four plants presented lower RRs. The most frequent combinations were: *ATRACTYLODIS MACROCEPHALAE RHIZOMA* + *GLYCYRRHIZAE RADIX ET RHIZOMA* + *PORIA*+ *PINELLIAE RHIZOMA* (n=4) (RR 1.27 [1.02, 1.57], I^2^ = 0%), and *GLYCYRRHIZAE RADIX ET RHIZOMA* + *PORIA* + *CODONOPSIS RADIX*+ *PINELLIAE RHIZOMA* (n=4) (RR 1.27 [1.02, 1.57], I^2^ = 0%). Compared with the total pool RR, 6 combinations of five plants presented lower RRs. The most frequent combinations were: *GLYCYRRHIZAE RADIX ET RHIZOMA* + *PORIA* + *CODONOPSIS RADIX*+ *PINELLIAE RHIZOMA* + *CITRI RETICULATAE PERICARPIUM* (n=3) (RR 1.18 [0.94, 1.48], I^2^ = 0%). Compared with the total pool RR, 1 combination of six plants presented lower RRs. The combination was: *ATRACTYLODIS MACROCEPHALAE RHIZOMA + ASTRAGALI RADIX+GLYCYRRHIZAE RADIX ET RHIZOMA+HEDYOTIS DIFFUSA+LIGUSTRI LUCIDI FRUCTUS +CREMASTRAE PSEUDOBULBUS PLEIONES PSEUDOBULBUS* (n=2) (RR 1.16 [0.92, 1.47], I^2^ = 0%). Liu.H.2018 (22) and Liu.Q.2016 (35) has the same ingredients of TMPs and their combination of herbs was therefore directly generalized to level 8. The combination was: *ATRACTYLODIS MACROCEPHALAE RHIZOMA* + *GLYCYRRHIZAE RADIX ET RHIZOMA* + P*ORIA* + C*ODONOPSIS RADIX*+ P*INELLIAE RHIZOMA* + *CITRI RETICULATAE PERICARPIUM*+ B*UPLEURI RADIX* + *PAEONIAE RADIX ALBA* (n=2) (RR 1.21 [0.91, 1.62], I^2^ = 0%).

**Table 6a:** Effects of specific TMPs on ORR for APC: single herb and combinations.

Level	TMPs	RR (95% CI)	N. stud. (Ref)	N. part.	I^2^
1	*ATRACTYLODIS MACROCEPHALAE RHIZOMA* (bai zhu)	1.66 [1.20, 2,28]	9 (22, 23, 25, 26, 28, 29, 31, 35, 37)	491	9
1	*PORIA* (fu ling)	1.52 [1.07, 2.15]	7 (22, 23, 25, 26, 31, 35, 37)	338	0
1	*PINELLIAE RHIZOMA* (ban xia)	1.67 [1.06, 2.62]	6 (22, 25, 28, 32, 35, 37)	300	0
1	*HEDYOTIS DIFFUSA* (baihua sheshe cao)	1.42 [0.91, 2.21]	4 (23–25, 29)	249	6
1	*SOPHORAE FLAVESCENTIS RADIX* (ku shen)	1.52 [1.19, 1.93]	4 (39–42)	278	0
1	*SCUTELLARIAE BARBATAE HERBA* (ban zhilian)	1.61 [0.99, 2.63]	2 (23, 36)	119	0
2	*ATRACTYLODIS MACROCEPHALAE RHIZOMA + PORIA*	1.66 [1.20, 2.28]	7 (22, 23, 25, 26, 31, 35, 37)	338	0

TMPs, traditional medicine preparations; RR, risk ratio; CI, confidence interval; N. stud., number of studies; N. part, number of participants; Ref., reference.

**Table 6b:** Effects of specific TMPs on DCR for APC: single herb and combinations.

Level	TMPs	RR (95% CI)	N. stud. (Ref)	N. part.	I^2^
1	*ATRACTYLODIS MACROCEPHALAE RHIZOMA* (bai zhu)	1.31 [1.18, 1,42]	10 (22, 23, 25–29, 31, 35, 37)	531	10
1	*ASTRAGALI RADIX* (huang qi)	1.22 [1.11, 1.33]	9 (23, 25, 29, 31, 32, 40–42, 49)	687	0
1	*GLYCYRRHIZAE RADIX ET RHIZOMA* (gan cao)	1.28 [1.10, 1.49]	7 (22, 25, 27, 29, 32, 35, 37)	365	0
1	*PORIA* (fu ling)	1.29 [1.11, 1.51]	7 (22, 23, 25, 26, 31, 35, 37)	338	28
1	*GINSENG RADIX ET RHIZOMA* (ren shen)	1.20 [1.09, 1.32]	6 (27, 32, 40–42, 49)	468	0
1	*CODONOPSIS RADIX* (dang shen)	1.30 [1.17, 1.64]	7 (22, 25–27, 31, 35, 37)	393	0
1	*PINELLIAE RHIZOMA* (ban xia)	1.33 [1.12, 1.57]	6 (22, 25, 28, 32, 35, 37)	300	0
1	*HEDYOTIS DIFFUSA* (baihua sheshe cao)	1.19 [1.03, 1.38]	4 (23–25, 29)	249	0
1	*BUPLEURI RADIX* (chai hu)	1.27 [1.04, 1.56]	3 (22, 32, 35)	159	0
1	*CITRI RETICULATAE PERICARPIUM* (chen pi)	1.18 [0.94, 1.48]	3 (22, 25, 35)	147	0
1	*CURCUMAE RHIZOMA* (e zhu)	1.26 [1.01, 1.57]	2 (23, 29)	151	0
1	*SOPHORAE FLAVESCENTIS RADIX* (ku shen)	1.14 [1.02, 1.28]	4 (39–42)	278	0
1	*SPARGANII RHIZOMA* (san leng)	1.26 [1.01, 1.57]	2 (23, 29)	151	0
1	*PAEONIAE RADIX ALBA* (bai shao)	1.21 [0.91, 1.62]	2 (22, 35)	99	0
1	*SOLANUM LYRATUM THUNB* (bai ying)	1.26 [1.01, 1.57]	2 (23, 29)	151	0
1	*ANGELICAE SINENSIS RADIX* (dang gui)	1.36 [1.10, 1.68]	2 (23, 32)	122	0
1	*LIGUSTRI LUCIDI FRUCTUS* (nv zhenzi)	1.16 [0.92, 1.47]	2 (25, 29)	137	0
1	*CREMASTRAE PSEUDOBULBUS PLEIONES PSEUDOBULBUS* (shan cigu)	1.16 [0.92, 1.47]	2 (25, 29)	137	0
1	*MYLABRIS* (ban mao)	1.21 [1.03, 1.41]	2 (49, 51)	274	0
2	*ATRACTYLODIS MACROCEPHALAE RHIZOMA + ASTRAGALI RADIX*	1.29 [1.07, 1.57]	4 (23, 25, 29, 31)	259	0
2	*ATRACTYLODIS MACROCEPHALAE RHIZOMA + GLYCYRRHIZAE RADIX ET RHIZOMA*	1.26 [1.06, 1.57]	6 (22, 25, 27, 29, 35, 37)	305	0
2	*ATRACTYLODIS MACROCEPHALAE RHIZOMA + PORIA*	1.29 [1.11, 1.51]	7 (22, 23, 25, 26, 31, 35, 37)	338	28
2	*ATRACTYLODIS MACROCEPHALAE RHIZOMA + PINELLIAE RHIZOMA*	1.32 [1.08, 1.61]	5 (22, 25, 28, 35, 37)	340	0
2	*ATRACTYLODIS MACROCEPHALAE RHIZOMA + HEDYOTIS DIFFUSA*	1.23 [1.02, 1.48]	3 (23, 25, 29)	199	0
2	*ATRACTYLODIS MACROCEPHALAE RHIZOMA + CURCUMAE RHIZOMA*	1.18 [0.94, 1.48]	3 (22, 25, 35)	147	0
2	*ATRACTYLODIS MACROCEPHALAE RHIZOMA + SPARGANII RHIZOMA*	1.26 [1.01, 1.57]	2 (23, 29)	151	0
2	*ATRACTYLODIS MACROCEPHALAE RHIZOMA + SOLANUM LYRATUM THUNB*	1.26 [1.01, 1.57]	2 (23, 29)	151	0
2	*ATRACTYLODIS MACROCEPHALAE RHIZOMA + LIGUSTRI LUCIDI FRUCTUS*	1.16 [0.92, 1.47]	2 (25, 29)	137	0
2	*ATRACTYLODIS MACROCEPHALAE RHIZOMA + CREMASTRAE PSEUDOBULBUS PLEIONES PSEUDOBULBUS*	1.16 [0.92, 1.47]	2 (25, 29)	137	0
2	*ASTRAGALI RADIX + GLYCYRRHIZAE RADIX ET RHIZOMA*	1.22 [1.02, 1.47]	3 (25, 29, 32)	197	0
2	*ASTRAGALI RADIX + GINSENG RADIX ET RHIZOMA*	1.18 [1.08, 1.30]	5 (32, 40–42, 49)	428	0
2	*ASTRAGALI RADIX + PINELLIAE RHIZOMA*	1.33 [1.09, 1.63]	4 (25, 32, 35, 37)	177	0
2	*ASTRAGALI RADIX + CURCUMAE RHIZOMA*	1.26 [1.01, 1.57]	2 (23, 29)	151	0
2	*ASTRAGALI RADIX + SOPHORAE FLAVESCENTIS RADIX*	1.19 [1.06, 1.35]	3 (40–42)	268	0
2	*ASTRAGALI RADIX + SPARGANII RHIZOMA*	1.26 [1.01, 1.57]	2 (23, 29)	151	0
2	*ASTRAGALI RADIX + SOLANUM LYRATUM THUNB*	1.26 [1.01, 1.57]	2 (23, 29)	151	0
2	*ASTRAGALI RADIX + ANGELICAE SINENSIS RADIX*	1,36 [1.10, 1.68]	2 (23, 32)	122	0
2	*ASTRAGALI RADIX + LIGUSTRI LUCIDI FRUCTUS*	1.16 [0.92, 1.47]	2 (25, 29)	137	0
2	*ASTRAGALI RADIX + CREMASTRAE PSEUDOBULBUS PLEIONES PSEUDOBULBUS*	1.16 [0.92, 1.47]	2 (25, 29)	137	0
2	*GLYCYRRHIZAE RADIX ET RHIZOMA + PORIA*	1.27 [1.02, 1.57]	4 (22, 25, 35, 37)	176	0
2	*GLYCYRRHIZAE RADIX ET RHIZOMA + CODONOPSIS RADIX*	1.30 [1.05, 1.61]	5 (22, 25, 27, 35, 37)	216	0
2	*GLYCYRRHIZAE RADIX ET RHIZOMA + PINELLIAE RHIZOMA*	1.29 [1.08, 1.53]	5 (22, 25, 32, 35, 37)	236	0
2	*GLYCYRRHIZAE RADIX ET RHIZOMA + HEDYOTIS DIFFUSA*	1.19 [1.03, 1.38]	4 (23–25, 29)	249	0
2	*GLYCYRRHIZAE RADIX ET RHIZOMA + BUPLEURI RADIX*	1.27 [1.04, 1.56]	3 (22, 32, 35)	169	0
2	*GLYCYRRHIZAE RADIX ET RHIZOMA + CITRI RETICULATAE PERICARPIUM*	1.18 [0.94, 1.48]	3 (22, 25, 35)	147	0
2	*GLYCYRRHIZAE RADIX ET RHIZOMA + LIGUSTRI LUCIDI FRUCTUS*	1.16 [0.92, 1.47]	2 (25, 29)	137	0
2	*GLYCYRRHIZAE RADIX ET RHIZOMA + CREMASTRAE PSEUDOBULBUS PLEIONES PSEUDOBULBUS*	1.16 [0.92, 1.47]	2 (25, 29)	137	0
2	*PORIA + PINELLIAE RHIZOMA*	1.27 [1.02, 1.57]	4 (22, 25, 35, 37)	176	0
2	*PORIA + HEDYOTIS DIFFUSA*	1.26 [0.99, 1.60]	2 (23, 25)	110	0
2	*PORIA + CITRI RETICULATAE PERICARPIUM*	1.18 [0.94, 1.48]	3 (22, 25, 35)	147	0
2	*GINSENG RADIX ET RHIZOMA + SOPHORAE FLAVESCENTIS RADIX*	1.15 [1.01, 1.32]	3 (40–42)	208	0
2	*CODONOPSIS RADIX + PINELLIAE RHIZOMA*	1.27 [1.02, 1.57]	4 (22, 25, 35, 37)	176	0
2	*CODONOPSIS RADIX + CITRI RETICULATAE PERICARPIUM*	1.18 [0.94, 1.48]	3 (22, 25, 35)	147	0
2	*PINELLIAE RHIZOMA + BUPLEURI RADIX*	1.27 [1.04, 1.56]	3 (22, 32, 35)	169	0
2	*PINELLIAE RHIZOMA + CITRI RETICULATAE PERICARPIUM*	1.18 [0.94, 1.48]	3 (22, 25, 35)	147	0
2	*HEDYOTIS DIFFUSA + CURCUMAE RHIZOMA*	1.26 [1.01, 1.57]	2 (23, 29)	151	0
2	*HEDYOTIS DIFFUSA + SPARGANII RHIZOMA*	1.26 [1.01, 1.57]	2 (23, 29)	151	0
2	*HEDYOTIS DIFFUSA + SOLANUM LYRATUM THUNB*	1.26 [1.01, 1.57]	2 (23, 29)	151	0
2	*HEDYOTIS DIFFUSA + LIGUSTRI LUCIDI FRUCTUS*	1.16 [0.92, 1.47]	2 (25, 29)	137	0
2	*HEDYOTIS DIFFUSA + CREMASTRAE PSEUDOBULBUS PLEIONES PSEUDOBULBUS*	1.16 [0.92, 1.47]	2 (25, 29)	137	0
2	*CURCUMAE RHIZOMA + SPARGANII RHIZOMA*	1.26 [1.01, 1.57]	2 (23, 29)	151	0
2	*CURCUMAE RHIZOMA + SOLANUM LYRATUM THUNB*	1.26 [1.01, 1.57]	2 (23, 29)	151	0
2	*SPARGANII RHIZOMA+ SOLANUM LYRATUM THUNB*	1.26 [1.01, 1.57]	2 (23, 29)	151	0
2	*LIGUSTRI LUCIDI FRUCTUS + CREMASTRAE PSEUDOBULBUS PLEIONES PSEUDOBULBUS*	1.16 [0.92, 1.47]	2 (25, 29)	137	0
3	*ATRACTYLODIS MACROCEPHALAE RHIZOMA + ASTRAGALI RADIX+GLYCYRRHIZAE RADIX ET RHIZOMA*	1.16 [0.92, 1.47]	2 (25, 29)	137	0
3	*ATRACTYLODIS MACROCEPHALAE RHIZOMA + ASTRAGALI RADIX+HEDYOTIS DIFFUSA*	1.23 [1.02, 1.48]	3 (23, 25, 29)	199	0
3	*ATRACTYLODIS MACROCEPHALAE RHIZOMA + ASTRAGALI RADIX+SPARGANII RHIZOMA*	1.26 [1.01, 1.57]	2 (23, 29)	151	0
3	*ATRACTYLODIS MACROCEPHALAE RHIZOMA + ASTRAGALI RADIX+SOLANUM LYRATUM THUNB*	1.26 [1.01, 1.57]	2 (23, 29)	151	0
3	*ATRACTYLODIS MACROCEPHALAE RHIZOMA + ASTRAGALI RADIX+LIGUSTRI LUCIDI FRUCTUS*	1.16 [0.92, 1.47]	2 (25, 29)	137	0
3	*ATRACTYLODIS MACROCEPHALAE RHIZOMA + ASTRAGALI RADIX+CREMASTRAE PSEUDOBULBUS PLEIONES PSEUDOBULBUS*	1.16 [0.92, 1.47]	2 (25, 29)	137	0
3	*ATRACTYLODIS MACROCEPHALAE RHIZOMA + GLYCYRRHIZAE RADIX ET RHIZOMA + PORIA*	1.27 [1.02, 1.57]	4 (22, 25, 35, 37)	176	0
3	*ATRACTYLODIS MACROCEPHALAE RHIZOMA + GLYCYRRHIZAE RADIX ET RHIZOMA + PINELLIAE RHIZOMA*	1.27 [1.02, 1.57]	4 (22, 25, 35, 37)	176	0
3	*ATRACTYLODIS MACROCEPHALAE RHIZOMA + GLYCYRRHIZAE RADIX ET RHIZOMA + CURCUMAE RHIZOMA*	1.18 [0.94, 1.48]	3 (22, 25, 35)	147	0
3	*ATRACTYLODIS MACROCEPHALAE RHIZOMA + GLYCYRRHIZAE RADIX ET RHIZOMA + LIGUSTRI LUCIDI FRUCTUS*	1.16 [0.92, 1.47]	2 (25, 29)	137	0
3	*ATRACTYLODIS MACROCEPHALAE RHIZOMA + GLYCYRRHIZAE RADIX ET RHIZOMA + CREMASTRAE PSEUDOBULBUS PLEIONES PSEUDOBULBUS*	1.16 [0.92, 1.47]	2 (25, 29)	137	0
3	*ATRACTYLODIS MACROCEPHALAE RHIZOMA + PORIA + PINELLIAE RHIZOMA*	1.27 [1.02, 1.57]	4 (22, 25, 35, 37)	176	0
3	*ATRACTYLODIS MACROCEPHALAE RHIZOMA + PORIA + CURCUMAE RHIZOMA*	1.18 [0.94, 1.48]	3 (22, 25, 35)	147	0
3	*ATRACTYLODIS MACROCEPHALAE RHIZOMA + PINELLIAE RHIZOMA + CURCUMAE RHIZOMA*	1.18 [0.94, 1.48]	3 (22, 25, 35)	147	0
3	*ATRACTYLODIS MACROCEPHALAE RHIZOMA + HEDYOTIS DIFFUSA + SPARGANII RHIZOMA*	1.26 [1.01, 1.57]	2 (23, 29)	151	0
3	*ATRACTYLODIS MACROCEPHALAE RHIZOMA + HEDYOTIS DIFFUSA + SOLANUM LYRATUM THUNB*	1.26 [1.01, 1.57]	2 (23, 29)	151	0
3	*ATRACTYLODIS MACROCEPHALAE RHIZOMA + HEDYOTIS DIFFUSA + LIGUSTRI LUCIDI FRUCTUS*	1.16 [0.92, 1.47]	2 (25, 29)	137	0
3	*ATRACTYLODIS MACROCEPHALAE RHIZOMA + HEDYOTIS DIFFUSA + CREMASTRAE PSEUDOBULBUS PLEIONES PSEUDOBULBUS*	1.16 [0.92, 1.47]	2 (25, 29)	137	0
3	*ATRACTYLODIS MACROCEPHALAE RHIZOMA + SPARGANII RHIZOMA + SOLANUM LYRATUM THUNB*	1.26 [1.01, 1.57]	2 (23, 29)	151	0
3	*ATRACTYLODIS MACROCEPHALAE RHIZOMA + LIGUSTRI LUCIDI FRUCTUS*	1.16 [0.92, 1.47]	2 (25, 29)	137	0
3	*ASTRAGALI RADIX + GLYCYRRHIZAE RADIX ET RHIZOMA + PINELLIAE RHIZOMA*	1.25 [1.00, 1.56]	2 (25, 32)	108	0
3	*ASTRAGALI RADIX + GLYCYRRHIZAE RADIX ET RHIZOMA + LIGUSTRI LUCIDI FRUCTUS*	1.16 [0.92, 1.47]	2 (25, 29)	137	0
3	*ASTRAGALI RADIX + GLYCYRRHIZAE RADIX ET RHIZOMA + CREMASTRAE PSEUDOBULBUS PLEIONES PSEUDOBULBUS*	1.16 [0.92, 1.47]	2 (25, 29)	137	0
3	*ASTRAGALI RADIX + GINSENG RADIX ET RHIZOMA + SOPHORAE FLAVESCENTIS RADIX*	1.15 [1.01, 1.32]	3 (40–42)	208	0
3	*ASTRAGALI RADIX + CURCUMAE RHIZOMA + SPARGANII RHIZOMA*	1.26 [1.01, 1.57]	2 (23, 29)	151	0
3	*ASTRAGALI RADIX + CURCUMAE RHIZOMA + SOLANUM LYRATUM THUNB*	1.26 [1.01, 1.57]	2 (23, 29)	151	0
3	*ASTRAGALI RADIX + SPARGANII RHIZOMA + SOLANUM LYRATUM THUNB*	1.26 [1.01, 1.57]	2 (23, 29)	151	0
3	*ASTRAGALI RADIX + LIGUSTRI LUCIDI FRUCTUS + CREMASTRAE PSEUDOBULBUS PLEIONES PSEUDOBULBUS*	1.16 [0.92, 1.47]	2 (25, 29)	137	0
3	*GLYCYRRHIZAE RADIX ET RHIZOMA + PORIA + CODONOPSIS RADIX*	1.27 [1.02, 1.57]	4 (22, 25, 35, 37)	176	0
3	*GLYCYRRHIZAE RADIX ET RHIZOMA + PORIA + PINELLIAE RHIZOMA*	1.27 [1.02, 1.57]	4 (22, 25, 35, 37)	176	0
3	*GLYCYRRHIZAE RADIX ET RHIZOMA + PORIA + CITRI RETICULATAE PERICARPIUM*	1.18 [0.94, 1.48]	3 (22, 25, 35)	147	0
3	*GLYCYRRHIZAE RADIX ET RHIZOMA + CODONOPSIS RADIX + PINELLIAE RHIZOMA*	1.27 [1.02, 1.57]	4 (22, 25, 35, 37)	176	0
3	*GLYCYRRHIZAE RADIX ET RHIZOMA + CODONOPSIS RADIX + CITRI RETICULATAE PERICARPIUM*	1.18 [0.94, 1.48]	3 (22, 25, 35)	147	0
3	*GLYCYRRHIZAE RADIX ET RHIZOMA + PINELLIAE RHIZOMA + BUPLEURI RADIX*	1.27 [1.04, 1.56]	3 (22, 32, 35)	169	0
3	*GLYCYRRHIZAE RADIX ET RHIZOMA + PINELLIAE RHIZOMA + CITRI RETICULATAE PERICARPIUM*	1.18 [0.94, 1.48]	3 (22, 25, 35)	147	0
3	*GLYCYRRHIZAE RADIX ET RHIZOMA+ LIGUSTRI LUCIDI FRUCTUS + CREMASTRAE PSEUDOBULBUS PLEIONES PSEUDOBULBUS*	1.16 [0.92, 1.47]	2 (25, 29)	137	0
3	*PORIA + PINELLIAE RHIZOMA + CITRI RETICULATAE PERICARPIUM*	1.18 [0.94, 1.48]	3 (22, 25, 35)	147	0
3	*CODONOPSIS RADIX + PINELLIAE RHIZOMA + CITRI RETICULATAE PERICARPIUM*	1.18 [0.94, 1.48]	3 (22, 25, 35)	147	0
3	*HEDYOTIS DIFFUSA + CURCUMAE RHIZOMA + SPARGANII RHIZOMA*	1.26 [1.01, 1.57]	2 (23, 29)	151	0
3	*HEDYOTIS DIFFUSA + CURCUMAE RHIZOMA + SOLANUM LYRATUM THUNB*	1.26 [1.01, 1.57]	2 (23, 29)	151	0
3	*HEDYOTIS DIFFUSA + SPARGANII RHIZOMA + SOLANUM LYRATUM THUNB*	1.26 [1.01, 1.57]	2 (23, 29)	151	0
3	*HEDYOTIS DIFFUSA + LIGUSTRI LUCIDI FRUCTUS + CREMASTRAE PSEUDOBULBUS PLEIONES PSEUDOBULBUS*	1.16 [0.92, 1.47]	2 (25, 29)	137	0
3	*CURCUMAE RHIZOMA + CURCUMAE RHIZOMA + SPARGANII RHIZOMA*	1.26 [1.01, 1.57]	2 (23, 29)	151	0
4	*ATRACTYLODIS MACROCEPHALAE RHIZOMA + ASTRAGALI RADIX+GLYCYRRHIZAE RADIX ET RHIZOMA+HEDYOTIS DIFFUSA*	1.16 [0.92, 1.47]	2 (25, 29)	137	0
4	*ATRACTYLODIS MACROCEPHALAE RHIZOMA + ASTRAGALI RADIX+GLYCYRRHIZAE RADIX ET RHIZOMA+LIGUSTRI LUCIDI FRUCTUS*	1.16 [0.92, 1.47]	2 (25, 29)	137	0
4	*ATRACTYLODIS MACROCEPHALAE RHIZOMA + ASTRAGALI RADIX+GLYCYRRHIZAE RADIX ET RHIZOMA+CREMASTRAE PSEUDOBULBUS PLEIONES PSEUDOBULBUS*	1.16 [0.92, 1.47]	2 (25, 29)	137	0
4	*ATRACTYLODIS MACROCEPHALAE RHIZOMA + ASTRAGALI RADIX+HEDYOTIS DIFFUSA +SPARGANII RHIZOMA*	1.26 [1.01, 1.57]	2 (23, 29)	151	0
4	*ATRACTYLODIS MACROCEPHALAE RHIZOMA + ASTRAGALI RADIX+HEDYOTIS DIFFUSA +SOLANUM LYRATUM THUNB*	1.26 [1.01, 1.57]	2 (23, 29)	151	0
4	*ATRACTYLODIS MACROCEPHALAE RHIZOMA + ASTRAGALI RADIX+HEDYOTIS DIFFUSA +LIGUSTRI LUCIDI FRUCTUS*	1.16 [0.92, 1.47]	2 (25, 29)	137	0
4	*ATRACTYLODIS MACROCEPHALAE RHIZOMA + ASTRAGALI RADIX+HEDYOTIS DIFFUSA +CREMASTRAE PSEUDOBULBUS PLEIONES*	1.16 [0.92, 1.47]	2 (25, 29)	137	0
4	*ATRACTYLODIS MACROCEPHALAE RHIZOMA + ASTRAGALI RADIX+SPARGANII RHIZOMA+SOLANUM LYRATUM THUNB*	1.26 [1.01, 1.57]	2 (23, 29)	151	0
4	*ATRACTYLODIS MACROCEPHALAE RHIZOMA + ASTRAGALI RADIX+LIGUSTRI LUCIDI FRUCTUS+CREMASTRAE PSEUDOBULBUS PLEIONES PSEUDOBULBUS*	1.16 [0.92, 1.47]	2 (25, 29)	137	0
4	*ATRACTYLODIS MACROCEPHALAE RHIZOMA + GLYCYRRHIZAE RADIX ET RHIZOMA + PORIA+ PINELLIAE RHIZOMA*	1.27 [1.02, 1.57]	4 (22, 25, 35, 37)	176	0
4	*ATRACTYLODIS MACROCEPHALAE RHIZOMA + GLYCYRRHIZAE RADIX ET RHIZOMA + PORIA+ CURCUMAE RHIZOMA*	1.18 [0.94, 1.48]	3 (22, 25, 35)	147	0
4	*ATRACTYLODIS MACROCEPHALAE RHIZOMA + GLYCYRRHIZAE RADIX ET RHIZOMA + PINELLIAE RHIZOMA + CURCUMAE RHIZOMA*	1.18 [0.94, 1.48]	3 (22, 25, 35)	147	0
4	*ATRACTYLODIS MACROCEPHALAE RHIZOMA + GLYCYRRHIZAE RADIX ET RHIZOMA + LIGUSTRI LUCIDI FRUCTUS + CREMASTRAE PSEUDOBULBUS PLEIONES*	1.16 [0.92, 1.47]	2 (25, 29)	137	0
4	*ATRACTYLODIS MACROCEPHALAE RHIZOMA + PORIA + PINELLIAE RHIZOMA+ CURCUMAE RHIZOMA*	1.18 [0.94, 1.48]	3 (22, 25, 35)	147	0
4	*ATRACTYLODIS MACROCEPHALAE RHIZOMA + HEDYOTIS DIFFUSA + SPARGANII RHIZOMA+ SOLANUM LYRATUM THUNB*	1.26 [1.01, 1.57]	2 (23, 29)	151	0
4	*ATRACTYLODIS MACROCEPHALAE RHIZOMA + HEDYOTIS DIFFUSA + LIGUSTRI LUCIDI FRUCTUS+ CREMASTRAE PSEUDOBULBUS PLEIONES PSEUDOBULBUS*	1.16 [0.92, 1.47]	2 (25, 29)	137	0
4	*ASTRAGALI RADIX + GLYCYRRHIZAE RADIX ET RHIZOMA + LIGUSTRI LUCIDI FRUCTUS + CREMASTRAE PSEUDOBULBUS PLEIONES PSEUDOBULBUS*	1.16 [0.92, 1.47]	2 (25, 29)	137	0
4	*ASTRAGALI RADIX + CURCUMAE RHIZOMA + SPARGANII RHIZOMA+ SOLANUM LYRATUM THUNB*	1.26 [1.01, 1.57]	2 (23, 29)	151	0
4	*GLYCYRRHIZAE RADIX ET RHIZOMA + PORIA + CODONOPSIS RADIX+ PINELLIAE RHIZOMA*	1.27 [1.02, 1.57]	4 (22, 25, 35, 37)	176	0
4	*GLYCYRRHIZAE RADIX ET RHIZOMA + PORIA + CODONOPSIS RADIX+ CITRI RETICULATAE PERICARPIUM*	1.18 [0.94, 1.48]	3 (22, 25, 35)	147	0
4	*GLYCYRRHIZAE RADIX ET RHIZOMA + PORIA + PINELLIAE RHIZOMA + CITRI RETICULATAE PERICARPIUM*	1.18 [0.94, 1.48]	3 (22, 25, 35)	147	0
4	*GLYCYRRHIZAE RADIX ET RHIZOMA + CODONOPSIS RADIX + PINELLIAE RHIZOMA+ CITRI RETICULATAE PERICARPIUM*	1.18 [0.94, 1.48]	3 (22, 25, 35)	147	0
4	*HEDYOTIS DIFFUSA + CURCUMAE RHIZOMA + SPARGANII RHIZOMA+ SOLANUM LYRATUM THUNB*	1.26 [1.01, 1.57]	2 (23, 29)	151	0
5	*ATRACTYLODIS MACROCEPHALAE RHIZOMA + ASTRAGALI RADIX+GLYCYRRHIZAE RADIX ET RHIZOMA+HEDYOTIS DIFFUSA+LIGUSTRI LUCIDI FRUCTUS*	1.16 [0.92, 1.47]	2 (25, 29)	137	0
5	*ATRACTYLODIS MACROCEPHALAE RHIZOMA + ASTRAGALI RADIX+GLYCYRRHIZAE RADIX ET RHIZOMA+HEDYOTIS DIFFUSA+CREMASTRAE PSEUDOBULBUS PLEIONES PSEUDOBULBUS*	1.16 [0.92, 1.47]	2 (25, 29)	137	0
5	*ATRACTYLODIS MACROCEPHALAE RHIZOMA + ASTRAGALI RADIX+GLYCYRRHIZAE RADIX ET RHIZOMA+LIGUSTRI LUCIDI FRUCTUS+CREMASTRAE PSEUDOBULBUS PLEIONES PSEUDOBULBUS*	1.16 [0.92, 1.47]	2 (25, 29)	137	0
5	*ATRACTYLODIS MACROCEPHALAE RHIZOMA + ASTRAGALI RADIX+HEDYOTIS DIFFUSA +SPARGANII RHIZOMA+SOLANUM LYRATUM THUNB*	1.26 [1.01, 1.57]	2 (23, 29)	151	0
5	*ATRACTYLODIS MACROCEPHALAE RHIZOMA + ASTRAGALI RADIX+HEDYOTIS DIFFUSA +LIGUSTRI LUCIDI FRUCTUS+CREMASTRAE PSEUDOBULBUS PLEIONES*	1.16 [0.92, 1.47]	2 (25, 29)	137	0
5	*GLYCYRRHIZAE RADIX ET RHIZOMA + PORIA + CODONOPSIS RADIX+ PINELLIAE RHIZOMA + CITRI RETICULATAE PERICARPIUM*	1.18 [0.94, 1.48]	3 (22, 25, 35)	147	0
6	*ATRACTYLODIS MACROCEPHALAE RHIZOMA + ASTRAGALI RADIX+GLYCYRRHIZAE RADIX ET RHIZOMA+HEDYOTIS DIFFUSA+LIGUSTRI LUCIDI FRUCTUS +CREMASTRAE PSEUDOBULBUS PLEIONES PSEUDOBULBUS*	1.16 [0.92, 1.47]	2 (25, 29)	137	0
8	*ATRACTYLODIS MACROCEPHALAE RHIZOMA + GLYCYRRHIZAE RADIX ET RHIZOMA + PORIA + CODONOPSIS RADIX+ PINELLIAE RHIZOMA + CITRI RETICULATAE PERICARPIUM+ BUPLEURI RADIX + PAEONIAE RADIX ALBA*	1.21 [0.91, 1.62]	2 (22, 35)	99	0

TMPs, traditional medicine preparations; RR, risk ratio; CI, confidence interval; N. stud., number of studies; N. part., number of participants; Ref., reference.

### 3.9 Sensitivity Analysis

We analyzed the sensitivity of the main outcome indicators, including ORR and DCR, by excluding each trial to check the robustness of the results. The results showed that the pooled RR values of the ORR and DCR were stable.

### 3.10 Publication Bias

According to the contour-enhanced plot of ORR (**Figure S20**) and DCR (**Figure S21**), some trim-and-fill data fell in the area of no statistical significance, indicating that some negative results were not published, possibly leading to publication bias. Further Egger’s test (**Table 7**) showed no significant publication bias in the meta-analysis of ORR (p = 0.1200), whereas significant publication bias existed in DCR (p = 0.0001).

**Table 7:** Egger’s test of ORR and DCR.

Indicators	P value
ORR	0.1200
DCR	0.0001

ORR, objective response rate; DCR, disease control rate.

### 3.11 Quality of Evidence

As shown in **Tables 8A** and **8B**, the quality of evidence was moderate for ORR, leukopenia, nausea and vomiting, hair loss, and QoL (continuous data); low for DCR, QoL (dichotomous data), decreased hemoglobin, thrombopenia, myelosuppression, liver dysfunction, and renal dysfunction and very low for gastrointestinal reaction, CA19-9, and CEA.

**Table 8a:** GRADE evidence profile of clinical efficacy and safety.

Outcomes (Trials)	Quality assessment					No. of patients		Effect		Quality of evdence
	Risk of bias	Inconsistency	Indirectness	Imprecision	Publication bias	TMPs plus Chemotherapy	Chemotherapy Alone	Risk ratios(95% CI)	Anticipated absolute effects	
ORR (28)	Serious^a^	NO	NO	NO	NO	328/888 (36.9%)	196/851 (23.0%)	RR 1.64 (1.43 to 1.88)	147 more per 1000 (from 99 more to 203 more)	⊕⊕⊕Ο MODERATE
DCR (28)	Serious^a^	NO	NO	NO	Serious^e^	637/869 (73.3%)	476/834 (57.1%)	RR 1.29 (1.21 to 1.38)	166 more per 1000 (from 120 more to 217 more)	⊕⊕ΟΟ LOW
QOL (dichotomous data) (5)	Serious^b^	NO	NO	Serious^d^	NO	127/165 (77.0%)	87/163 (53.4%)	RR 1.44 (1.22 to 1.70)	235 more per 1000 (from 117 more to 374 more)	⊕⊕ΟΟ LOW
Leukopenia (6)	Serious^b^	NO	NO	NO	NO	21/246 (8.5%)	49/246 (19.9%)	RR 0.43 (0.27 to 0.7)	114 fewer per 1000 (from 60 fewer to 145 fewer)	⊕⊕⊕Ο MODERATE
Decreased hemoglobin (7)	Very serious^c^	NO	NO	NO	NO	34/300 (11.3%)	46/271 (17.0%)	RR 0.61 (0.40 to 0.94)	66 fewer per 1000 (from 10 fewer to 102 fewer)	⊕⊕ΟΟ LOW
Thrombopenia (8)	Very serious^c^	NO	NO	NO	NO	30/335 (9%)	51/306 (16.7%)	RR 0.54 (0.35 to 0.84)	77 fewer per 1000 (from 27 fewer to 108 fewer)	⊕⊕ΟΟ LOW
Myelosuppression (5)	Serious^b^	NO	NO	Serious^d^	NO	5/123 (4.1%)	9/120 (7.5%)	RR 0.56 (0.2 to 1.53)	33 fewer per 1000 (from 60 fewer to 40 more)	⊕⊕ΟΟ LOW
Nausea and vomiting (5)	Serious^b^	NO	NO	NO	NO	17/216 (7.9%)	26/215 (12.1%)	RR 0.67 (0.38 to 1.17)	40 fewer per 1000 (from 75 fewer to 21 more)	⊕⊕⊕Ο MODERATE
Gastrointestinal reaction (5)	Very serious^c^	NO	NO	Serious^d^	NO	4/123 (3.3%)	13/120 (10.8%)	RR 0.33 (0.12 to 0.9)	73 fewer per 1000 (from 11 fewer to 95 fewer)	⊕ΟΟΟ VERY LOW
Liver dysfunction (5)	Serious^b^	NO	NO	Serious^d^	NO	0/135 (0%)	2/132 (1.5%)	RR 0.19 (0.01 to 3.8)	12 fewer per 1000 (from 15 fewer to 42 fewer)	⊕⊕ΟΟ LOW
Renal dysfunction (3)	Serious^b^	NO	NO	Serious^d^	NO	0/84 (0%)	0/82 (0%)	not pooled	not pooled	⊕⊕ΟΟ LOW
Hair loss (3)	Serious^b^	NO	NO	NO	NO	3/165 (1.8%)	3/165 (1.8%)	RR 1 (0.21 to 4.86)	0 fewer per 1000 (from 14 fewer to 70 more)	⊕⊕⊕Ο MODERATE

a Most trials had unclear risk, and with high risk, but the result had good robustness. The evidence was rated down by only one level.

b Most trials had unclear risk and the trials were no high risk, but the result had good robustness. The evidence was rated down by only one level.

c Most trials had unclear risk and the trials were no high risk, but the result had poor robustness. The evidence was rated down by two levels.

d The sample size for each outcome was fewer than 300 cases. Therefore, the evidence was rated down by one level.

e There was publication bias. The QOL was over-estimated. The evidence was rated down by one level.

**Table 8b:** GRADE evidence profile of QOL (continuous data), and cancer biomarkers.

Outcomes (Trials)	Quality assessment					No. of patients		SMD (95% CI)	Quality of evdence
	Risk of bias	Inconsistency	Indirectness	Imprecision	Publication bias	TMPs plus Chemotherapy	Chemotherapy Alone		
QOL (continuous data) (9)	Serious^a^	NO^c^	NO	NO	NO	302	298	SMD 0.81 higher (0.44 to 1.18 higher)	⊕⊕⊕Ο MODERATE
CA19-9 (5)	Very serious^b^	Serious^d^	NO	NO	NO	187	184	SMD 0.46 lower (0.9 to 0.02 lower)	⊕ΟΟΟ VERY LOW
CEA (4)	Very serious^b^	Serious^d^	NO	NO	NO	157	154	SMD 0.55 lower (0.93 to 0.17 lower)	⊕ΟΟΟ VERY LOW

a Most trials had unclear risk and the trials were no high risk, but the result had good robustness. The evidence was rated down by only one level.

b Most trials had unclear risk and the trials were no high risk, but the result had poor robustness. The evidence was rated down by two levels.

c Heterogeneity presented in them, and the result had good robustness. Not rated down.

d Heterogeneity presented in them, and the result had poor robustness. The evidence was rated down by only one level.

## 4 Discussion

Natural products can serve as an important source of drug discovery. Many prescription medicines approved by the Food and Drug Administration for cancer treatment have been obtained from the natural products (55), and more than 50% of newly approved drugs between 1946 and 2019 were natural small molecules or their derivatives (56). TMPs are the products derived from the combination of natural products and traditional medicine theories. They have a complex chemical diversity that enables them to act on a variety of biological targets (enzymes, receptors, pathways, etc.) to achieve maximal efficacy in cancer therapy with minimal adverse reactions (57). Numerous studies have described the clinical efficacy and safety of TMPs for colorectal cancer (58), non-small cell lung cancer (59), and liver cancer (60), as well as for some cancer-related symptoms such as insomnia (61), pain (62), and anemia (63). Thus, mining TMPs with scientific and systematic methods can serve as an important strategy for cancer treatment. Pancreatic cancer is a fatal malignant tumor of the digestive system, and patients are usually in their advanced stages when diagnosed. TMPs combined with chemotherapy have been widely used in patients with APC to achieve greater survival benefit and QoL, but there is no reported systematic evaluation of whether these therapeutic regimens are significantly effective. Therefore, we have conducted this meta-analysis. As far as we know, this is the first systematic review and meta-analysis of RCTs describing the potential efficacy and safety of TMPs combined with chemotherapy in treating APC. The various outcomes of this meta-analysis include tumor response, QoL, cancer biomarkers and ADRs. A total of 28 different RCTs involving 1,832 APC individuals were included in this review.

At present, various TMPs containing diverse bioactive molecules have been shown to exhibit multiple anti-pancreatic cancer effects. These compounds, including quercetin (64), baicalein (65), honokiol (66), luteolin (67), and silibinin (68), have been found to be present in numerous TMPs. They can suppress pancreatic cancer cell proliferation or induce apoptotic and autophagic by modulating various oncogenic pathways including Wnt, phosphoinositide 3-kinase/protein kinase B/mammalian target of rapamycin, mitogen-activated protein kinases and Nuclear factor-kappa B pathways (69). For instance, fraxetin isolated from the bark of *Fraxinus bungeana A.DC.*, piperlongumine isolated from the fruit of the pepper *Piper longum*, and curcumin extracted from *Curcuma longa* were found to significantly enhance the anti- pancreatic cancer activity of gemcitabine (70–72). Therefore, the combination of TMPs and chemotherapy as therapeutic regimens has potential clinical value in APC treatment. This view has also been confirmed by various clinical trials in recent years (29, 34, 49). Our results showed that TMPs combined with chemotherapy can significantly enhance the tumor response, which was consistent with the previous experimental and clinical studies.

In addition, previous studies published have shown that ginkgo biloba extract (GBE 761 ONC) (73), Phytosome complex of curcumin (74), a Chinese botanical formula (PHY906) (75) were not only safe but also efficiently translate in a good response rate in the treatment of APC when combined with chemotherapy. In addition, the use of a modified supercritical carbon dioxide extract of Nerium oleander leaves (PBI-05204) (76) and Viscum album [L.] extract (77) alone has been reported to be beneficial for prolonging overall survival of patients with APC. However, none of them were RCTs about TMPs combined with chemotherapy in treating APC. These TMPs have the potential to treat APC, but it is unclear whether they effective and safet when combined with chemotherapy. The purpose of this study is to make more researchers pay attention to the good clinical value of TMPs combined with chemotherapy in treating APC.

TMPs consist of single or multiple herbs and are widely used in the clinical treatment for APC. The results of subgroup analysis showed that the following 6 herbs had significant combined RRs and no heterogeneity at multiple combined levels: *ATRACTYLODIS MACROCEPHALAE RHIZOMA, ASTRAGALI RADIX, GLYCYRRHIZAE RADIX ET RHIZOMA, PORIA, CODONOPSIS RADIX*, and *PINELLIAE RHIZOMA.* Therefore, these herbs were considered to have a consistent effect on enhancing the tumor response in multiple combinations which might be especially effective for treating APC when combined with chemotherapy and were more instructive to researchers. A study reported by Zhang et al. showed that calycosin, a bioactive isoflavonoid of *ASTRAGALI RADIX*, inhibited the growth of pancreatic cancer cells by inducing p21^Waf1/Cip1^-induced cell cycle arrest and caspase-dependent apoptosis (78). Cheng et al. concluded that a triterpene mixture extracted from *PORIA* inhibited the migration of pancreatic cancer cells associated with CDC20 (79). Moreover, Zhao C. found that licocoumarone, the extracts from *GLYCYRRHIZAE RADIX ET RHIZOMA*, suppressed human pancreatic adenocarcinoma BxPC-3 cell proliferation and induces cell apoptotic (80). However, there are less studies about the other three herbs and combinations of the six herbs in treating APC.

CA19-9 is a characteristic tumor biomarker of pancreatic cancer. The level of CA19-9 has been associated with tumor size (81), stage, and survival (82), as it is often used for diagnosis, prognosis and monitoring of patients with pancreatic cancer (83, 84). It has been observed that particularly, if the duration of the decline in CA19-9 levels was greater than 3 months during the 6 months period after initiation of the treatment, it could be significantly related to the good prognosis of APC (85). The previous studies have also shown that CA19-9 can effectively accelerate the process of pancreatic cancer by causing protein modification (86), binding to E-selectin, as well as by promoting angiogenesis, and is therefore considered as a potential target and an important research area for the treatment of APC (87). Our analysis results showed that TMPs combined with chemotherapy significantly reduced the levels of CA19-9, compared with chemotherapy alone, thereby indicating a positive effect of TMPs on the treatment of APC.

It has been established that ADRs during the treatment duration can influence the progress of treatment and the QoL of patients. Therefore, reducing the occurrence of ADRs is also an important task of clinicians. Our results suggested that patients treated with TMPs had a relatively lower incidence of leukopenia, decreased hemoglobin, thrombopenia, and gastrointestinal reaction, compared to the chemotherapy alone. Instead of increasing ADRs, addition of various TMPs as adjuvant and alternative drugs was found to markedly reduce ADRs which reflected the better safety profile of TMPs.

The included trials did not report TMPs-related adverse reactions. As the main compositions of TMPs in the treatment of APC, a number of previous studies have confirmed that *ATRACTYLODIS MACROCEPHALAE RHIZOMA* (88), *ASTRAGALI RADIX* (89), *PORIA* (90) and *CODONOPSIS RADIX* (91) do not exhibit significant toxicity and are safe for the clinical application. However, adverse reactions of some compositions of TMPs in included trials have been reported. For example, the most important side effects of *GLYCYRRHIZAE RADIX ET RHIZOMA* have been found to be hypertension and hypokalemic-induced secondary disorders and which need to be used with caution during pregnancy (92). Large-scale consumption of *GINSENG RADIX ET RHIZOMA* may cause anaphylaxis, palpitations, hypertension, skin hypersensitivity reactions and headache (93, 94). The toxicity of *PINELLIAE RHIZOMA* includes mucosal irritation, hepatorenal and gestational toxicity (95). While this does not mean that TMPs necessarily cause these adverse reactions, it must be used with caution before analyzing their safety through strict evaluation. It is very common to use TMPs for treatment of cancer patients. For instance, one study showed that herbal and supplementary medicine was used by 78% of patients undergoing chemotherapy, but 27% of them were assessed as at risk of adverse herb-chemotherapy interaction (96) which has become an important consideration in pharmacotherapy. Therefore, TMPs pose potential risks for interactions with chemotherapy drugs (97, 98), which are often caused by TMPs-related induction or inhibition of the drug metabolizing enzyme system cytochrome P-450 (CYP) and/or the P-glycoprotein drug efflux transport system (99). Herbal products that have shown clinical interactions with chemotherapeutic drugs include *ECHINACEA, ALLIUM SATIVUM, GINSENG, CITRUS PARADISI, SILYBUM MARIANUM* and *HYPERICUM PERFORATUM. GINSENG* is a commonly used herbal products used in the treatment of patients with APC, but the findings on its inductive effect on drug-metabolizing enzymes are relatively mixed (100, 101). In one case report, *ginseng* was observed to cause hepatotoxicity when used in combination with imatinib (102). Moreover, other herbal products commonly applied in APC, including *DIOSCOREA VILLOSA, RHODIOLA ROSEA* and *GANODERMA*, might also display strong potential for herb-chemotherapy interactions although there are no clinically relevant data (103). Considering the significant application of various TMPs for treating APC, clinicians and researchers should document in detail TMPs use during chemotherapy in patients with APC and be vigilant in monitoring for any potential interactions as well as adverse effects while administering TMPs- chemotherapy combination to the patients.

Our study has some potential limitations. First, we have only searched the English and Chinese databases which might miss some key trials published in other language. Second, the assessment of the methodological bias risk showed some bias in included trials thereby leading to low or very low quality of some potential outcomes. Regardless, the primary outcome was robust and reliable. We will include high-quality RCTs to update this study regularly. Third, there is currently some debate about whether TMPs combined with chemotherapy can significantly prolong the survival time in APC patients (34, 103–105). However, this study did not define it as an observational outcome due to presence of only few reports in the published trials. Therefore, further research is needed in the future. Fourth, the time elapsed from the chemotherapy termination until the measurements of outcomes were different because of the diverse chemotherapy regimens employed in each study. Although we observed the same trend of outcomes in the included trials, this factor might also substantially influence the interpretation of the results. Fifth, the specific mechanisms of action of TMPs are not clear although great progress has been made in the study of the effects of TMPs in treating APC. Sixth, according to the *CONSORT Extension for Chinese Herbal Medicine Formulas* (106), the name, provenance, dosage form, preparation method, dosage and route of administration of herbal medicine formulas should be reported in detail in RCT. Besides, the name, origin, processing method and dosage of all herbs in the formula should be reported. The certification method, quality control method and safety monitoring data for herbs and formulas should also be described. However, included trials using self-prepared herbal decoctions did not report the information above which might lead to potential bias. This indicated irregular reports in RCTs about self-prepared herbal decoctions in treating APC which need to be improved. Overall, we are hopeful that this study can provide relevant clinical evidences and experimental research direction for researchers. Finally, we expect that more attention will be paid to the potential therapeutic applications of TMPs in the treatment of APC and better deigned clinical trials will be conducted in future.

## 5 Conclusion

Our study confirmed the clinical efficacy and safety of TMPs combined with chemotherapy for APC. This combination regimen might benefit for the prognosis of patients with APC.

## Data Availability Statement

The original contributions presented in the study are included in the article/**Supplementary Material**. Further inquiries can be directed to the corresponding authors.

## Author Contributions

BH and HZ designed the research. JH, JJ, and MC performed literature search. GZ, SH, HY, BS, and JH performed article selection. JH, XZ, and RL assessed methodological bias risk. JH, JJ, and MC conducted a meta-analysis and assessed study quality. JH finished the manuscript draft. All authors contributed to the article and approved the submitted version.

## Funding

This study was supported by the Special training of scientific and technological talents, China Academy of Chinese Medical Sciences (Grant No. ZZ13-YQ-023), the National Natural Science Foundation of China (Grant No. 82174465), the Beijing Municipal Science and Technology Commission (Grant No. Z181100001618006), the Fundamental Research Funds for the Central Public Welfare Research Institutes (Grant No. ZZ13-YQ-028), the Youth project of National Natural Science Foundation of China (Grant No. 82104961) and the CACMS Innovation Fund (Grant No. CI2021A01814).

## Conflict of Interest

The authors declare that the research was conducted in the absence of any commercial or financial relationships that could be construed as a potential conflict of interest.

## Publisher’s Note

All claims expressed in this article are solely those of the authors and do not necessarily represent those of their affiliated organizations, or those of the publisher, the editors and the reviewers. Any product that may be evaluated in this article, or claim that may be made by its manufacturer, is not guaranteed or endorsed by the publisher.

## Supplementary Material

The detailed search strategy is available in Supplementary 1; the subgroup analysis of the QoL is available in Supplementary 2; the subgroup analysis of the cancer biomarkers is available in Supplementary 3; the figures of subgroup analysis of the ORR and DCR is available in Supplementary 4; the contour-enhanced plot is available in Supplementary 5.

## Abbreviations

APC, advanced pancreatic cancer; ADRs, adverse drug reactions; QoL, quality of life; TMPs, traditional medicine preparations; ORR, objective response rate; DCR, disease control rate; KPS, Karnofsky Performance Status; SD, standardized difference; RCTs, randomized controlled trials; RR, risk ratio; SMD, standardized mean difference; CIs, confidence intervals; FEM, fixed-effects model; REM, random-effects model.
