# Supplementary material for: Clinical Efficacy and Safety of Traditional Medicine Preparations Combined With Chemotherapy for Advanced Pancreatic Cancer: A Systematic Review and Meta-Analysis
Source: Front Oncol. 2022 Feb 23;12:828450. doi: 10.3389/fonc.2022.828450 (PMC8904728; doi:10.3389/fonc.2022.828450)
Supplement: Supplementary file 1 [file DataSheet_1.docx]

**Supplementary material 1. Detailed search strategy**

**Supplementary Table A: Search Strategy Used in PubMed 2021/10/15**

| No. | Search items | Items found |
| --- | --- | --- |
| #1 | ("neoplasms"[MeSH Terms] OR "carcinoma"[MeSH Terms] OR "adenocarcinoma"[MeSH Terms] OR "cancer*"[Title/Abstract] OR "carcin*"[Title/Abstract] OR "neoplas*"[Title/Abstract] OR "tumo*"[Title/Abstract] OR "adenocarcinoma*"[Title/Abstract]) AND ("pancreas"[MeSH Terms] OR "pancreas"[Title/Abstract] OR "pancreatic"[Title/Abstract]) | 125069 |
| #2 | "complementary therapies"[MeSH Terms] OR "drugs, Chinese herbal"[MeSH Terms] OR "herbal medicine"[MeSH Terms] OR "medicine, traditional"[MeSH Terms] OR "medicine, east asian traditional"[MeSH Terms] OR "plant extracts"[MeSH Terms] OR "plants, medicinal"[MeSH Terms] OR "phytotherapy"[MeSH Terms] OR "alternative medicine"[Title/Abstract] OR "complementary therap*"[Title/Abstract] OR "Chinese herba*"[Title/Abstract] OR "Chinese medicine"[Title/Abstract] OR "herb*"[Title/Abstract] OR "herbalism"[Title/Abstract] OR "herbal medicine"[Title/Abstract] OR "herbal drugs"[Title/Abstract] OR "plant extract*"[Title/Abstract] OR "medicinal plant*"[Title/Abstract] OR "phytotherapy*"[Title/Abstract] OR "phytopharmaceutic*"[Title/Abstract] OR "traditional medicine"[Title/Abstract] OR "oriental medicine"[Title/Abstract] OR "zhong yi xue"[Title/Abstract] | 535160 |
| #3 | ("randomized controlled trial"[Publication Type] OR "controlled clinical trial"[Publication Type] OR "randomized"[Title/Abstract] OR "placebo"[Title/Abstract] OR "drug therapy"[MeSH Terms] OR "randomly"[Title/Abstract] OR "trial"[Title/Abstract] OR "groups"[Title/Abstract]) NOT ("animals"[MeSH Terms] NOT "humans"[MeSH Terms]) | 3743300 |
| #4 | #1 and #2 and #3 | 178 |

**Supplementary Table B. Search Strategy Used in EMBASE 2021/10/16**

| **No.** | **Search items** | **Items found** |
| --- | --- | --- |
| **Search Terms to Pancreatic Cancer:** | | |
| #1 | 'neoplasm'/exp | 5528354 |
| #2 | 'carcinoma'/exp | 1422705 |
| #3 | 'adenocarcinoma'/exp | 268173 |
| #4 | cancer*:ti,ab,kw | 2901509 |
| #5 | carcin*:ti,ab,kw | 1214850 |
| #6 | neoplas*:ti,ab,kw | 525815 |
| #7 | tumo*:ti,ab,kw | 2629899 |
| #8 | adenocarcinoma*:ti,ab,kw | 249758 |
| #9 | #1 OR #2 OR #3 OR #4 OR #5 OR #6 OR #7 OR #8 | 6151454 |
| #10 | 'pancreas'/exp | 131898 |
| #11 | pancreas:ti,ab,kw | 162973 |
| #12 | pancreatic:ti,ab,kw | 305486 |
| #13 | #10 OR #11 OR #12 | 417187 |
| #14 | #9 AND #13 | 185755 |
| **Search Terms to Interventions:** | | |
| #15 | 'alternative medicine'/exp | 69580 |
| #16 | 'herbaceous agent'/exp | 55625 |
| #17 | 'herbal medicine'/exp | 24782 |
| #18 | 'traditional medicine'/exp | 115752 |
| #19 | 'oriental medicine'/exp | 2984 |
| #20 | 'chinese medicine'/exp | 59339 |
| #21 | 'plant extract'/exp | 247854 |
| #22 | 'medicinal plant'/exp | 279866 |
| #23 | 'phytotherapy'/exp | 18198 |
| #24 | 'alternative medicine':ti,ab,kw | 14672 |
| #25 | 'complementary therap*':ti,ab,kw | 6675 |
| #26 | 'chinese herba*':ti,ab,kw | 10554 |
| #27 | 'chinese medicine*':ti,ab,kw | 43722 |
| #28 | herb*:ti,ab,kw | 156249 |
| #29 | herbalism:ti,ab,kw | 251 |
| #30 | 'herbal medicine':ti,ab,kw | 19059 |
| #31 | 'herbal drugs':ti,ab,kw | 3110 |
| #32 | 'plant extract*':ti,ab,kw | 20359 |
| #33 | 'medicinal Plant*':ti,ab,kw | 39268 |
| #34 | 'Phytotherapy*':ti,ab,kw | 4113 |
| #35 | 'phytopharmaceutic*':ti,ab,kw | 1156 |
| #36 | ‘traditional medicine’:ti,ab,kw | 20557 |
| #37 | ‘oriental medicine’:ti,ab,kw | 1602 |
| #38 | ‘zhong yi xue’:ti,ab,kw | 1 |
| #39 | #15 OR #16 OR #17 OR #18 OR #19 OR #20 OR #21 OR #22 OR #23 OR #24 OR #25 OR #26 OR #27 OR #28 OR #29 OR #30 OR #31 OR #32 OR #33 OR #34 OR #35 OR #36 OR #37 OR #38 | 683296 |
| **Types of Study:** | | |
| #40 | random* | 1963270 |
| #41 | #14 AND #39 AND #40 | 152 |

**Supplementary Table C. Search Strategy Used in** **Cochrane 2021/10/16**

| **No.** | **Search items** | **Items found** |
| --- | --- | --- |
| **Search Terms to Pancreatic Cancer:** | | |
| #1 | MeSH descriptor: [Neoplasms] explode all trees | 84044 |
| #2 | MeSH descriptor: [Carcinoma] explode all trees | 14186 |
| #3 | MeSH descriptor: [Adenocarcinoma] explode all trees | 7862 |
| #4 | (cancer*):ti,ab,kw | 174063 |
| #5 | (carcin*):ti,ab,kw | 45551 |
| #6 | (neoplas*):ti,ab,kw | 86832 |
| #7 | (tumo*):ti,ab,kw | 77875 |
| #8 | (adenocarcinoma*):ti,ab,kw | 11360 |
| #9 | #1 OR #2 OR #3 OR #4 OR #5 OR #6 OR #7 OR #8 | 236594 |
| #10 | MeSH descriptor: [Pancreas] explode all trees | 1356 |
| #11 | (pancreas):ti,ab,kw | 7785 |
| #12 | (pancreatic):ti,ab,kw | 14188 |
| #13 | #10 OR #11 OR #12 | 17528 |
| #14 | #9 AND #13 | 7610 |
| **Search Terms to Interventions:** | | |
| #15 | MeSH descriptor: [Complementary Therapies] explode all trees | 21070 |
| #16 | MeSH descriptor: [Drugs, Chinese Herbal] explode all trees | 3705 |
| #17 | MeSH descriptor: [Herbal Medicine] explode all trees | 63 |
| #18 | MeSH descriptor: [Medicine, Traditional] explode all trees | 1582 |
| #19 | MeSH descriptor: [Medicine, East Asian Traditional] explode all trees | 1357 |
| #20 | MeSH descriptor: [Plant Extracts] explode all trees | 8586 |
| #21 | MeSH descriptor: [Plants, Medicinal] explode all trees | 947 |
| #22 | MeSH descriptor: [Phytotherapy] explode all trees | 4268 |
| #23 | (alternative medicine):ti,ab,kw | 5163 |
| #24 | (complementary therap*):ti,ab,kw | 4665 |
| #25 | (chinese herba*):ti,ab,kw | 5247 |
| #26 | (chinese medicine):ti,ab,kw | 12585 |
| #27 | (herb*):ti,ab,kw | 12083 |
| #28 | (herbalism):ti,ab,kw | 9762 |
| #29 | (herbal medicine):ti,ab,kw | 5171 |
| #30 | (herbal drugs):ti,ab,kw | 7303 |
| #31 | (plant extract*):ti,ab,kw | 6643 |
| #32 | (Medicinal Plant*):ti,ab,kw | 3537 |
| #33 | (Phytotherapy*):ti,ab,kw | 4387 |
| #34 | (phytopharmaceutic*):ti,ab,kw | 34 |
| #35 | (traditional medicine):ti,ab,kw | 11012 |
| #36 | (oriental medicine):ti,ab,kw | 681 |
| #37 | (zhong yi xue):ti,ab,kw | 3 |
| #38 | #15 OR #16 OR #17 OR #18 OR #19 OR #20 OR #21 OR #22 OR #23 OR #24 OR #25 OR #26 OR #27 OR #28 OR #29 OR #30 OR #31 OR #32 OR #33 OR #34 OR #35 OR #36 OR #37 | 53521 |
| #39 | #14 AND #37 | 124 |
