# Supplementary material for: Clinical Efficacy and Safety of Traditional Medicine Preparations Combined With Chemotherapy for Advanced Pancreatic Cancer: A Systematic Review and Meta-Analysis
Source: Front Oncol. 2022 Feb 23;12:828450. doi: 10.3389/fonc.2022.828450 (PMC8904728; doi:10.3389/fonc.2022.828450)
Supplement: Supplementary file 2 [file DataSheet_2.zip › Supplementary material 2/Supplementary material 2.docx]

**Supplementary material 2. Subgroup analysis results of QOL (continuous data) (Table S1)**

**Table S1. Subgroup analysis of the QOL**

| **Subgroups** | **Number of trials** | **SMD (95% CI)** | **Z** | ***p*** | **Heterogeneity** | | **TSD** |
| --- | --- | --- | --- | --- | --- | --- | --- |
|  |  |  |  |  | **I²** | ***P_h_*** |  |
| **Table S1a. Subgroups analysis according to KPS score (Fig S1)** | | | | | | |  |
| KPS score (50≤ and <60) | 1 | 1.02 [0.35, 1.68] | 3.00 | 0.003 | Not applicable | Not applicable | 0% |
| KPS score (≥60) | 8 | 0.79 [0.38, 1.20] | 3.76 | 0.0002 | 81% | <0.00001 |  |
| **Table S1b. Subgroups analysis according to drug delivery of TMPs (Fig S2)** | | | | | | |  |
| Intravenously | 1 | 1.59 [1.23, 1.94] | 8.72 | <0.00001 | Not applicable | Not applicable | 93.1% |
| Orally | 8 | 0.68 [0.39, 0.98] | 4.49 | <0.00001 | 56% | 0.02 |  |
| **Table S1c. Subgroups analysis according to the number of chemotherapy drug (Fig S3)** | | | | | | |  |
| Single - drug | 6 | 0.92 [0.42, 1.41] | 3.64 | 0.0003 | 80% | 0.0001 | 0% |
| Double - drugs | 3 | 0.58 [0.14, 1.03] | 2.59 | 0.009 | 54% | 0.11 |  |
| **Table S1d. Subgroups analysis according to chemotherapy regimen (Fig S4)** | | | | | | |  |
| GEM-based chemotherapy regimen | 3 | 0.58 [0.14, 1.03] | 2.59 | 0.009 | 54% | 0.11 | 9.2% |
| S-1-based chemotherapy regimen | 5 | 0.62 [0.13, 1.10] | 2.50 | 0.01 | 72% | 0.007 |  |
| Others | 2 | 1.22 [0.47, 1.98] | 3.16 | 0.002 | 82% | 0.02 |  |
| **Table S1e. Subgroups analysis according to follow-up time (Fig S5)** | | | | | | |  |
| 6≤ and <9w | 3 | 0.78 [0.48, 1.08] | 5.14 | <0.00001 | 0% | 0.68 | 0% |
| ≥9w | 6 | 0.80 [0.24, 1.37] | 2.77 | 0.006 | 86% | <0.00001 |  |

**Note:** RR: risk ratio, CI: confidence interval, QoL: quality of life, PT: primary treatment, TSD: Test for subgroup differences.
