# Supplementary figures and images for: Clinical Efficacy and Safety of Traditional Medicine Preparations Combined With Chemotherapy for Advanced Pancreatic Cancer: A Systematic Review and Meta-Analysis
Source: Front Oncol. 2022 Feb 23;12:828450. doi: 10.3389/fonc.2022.828450 (PMC8904728; doi:10.3389/fonc.2022.828450)

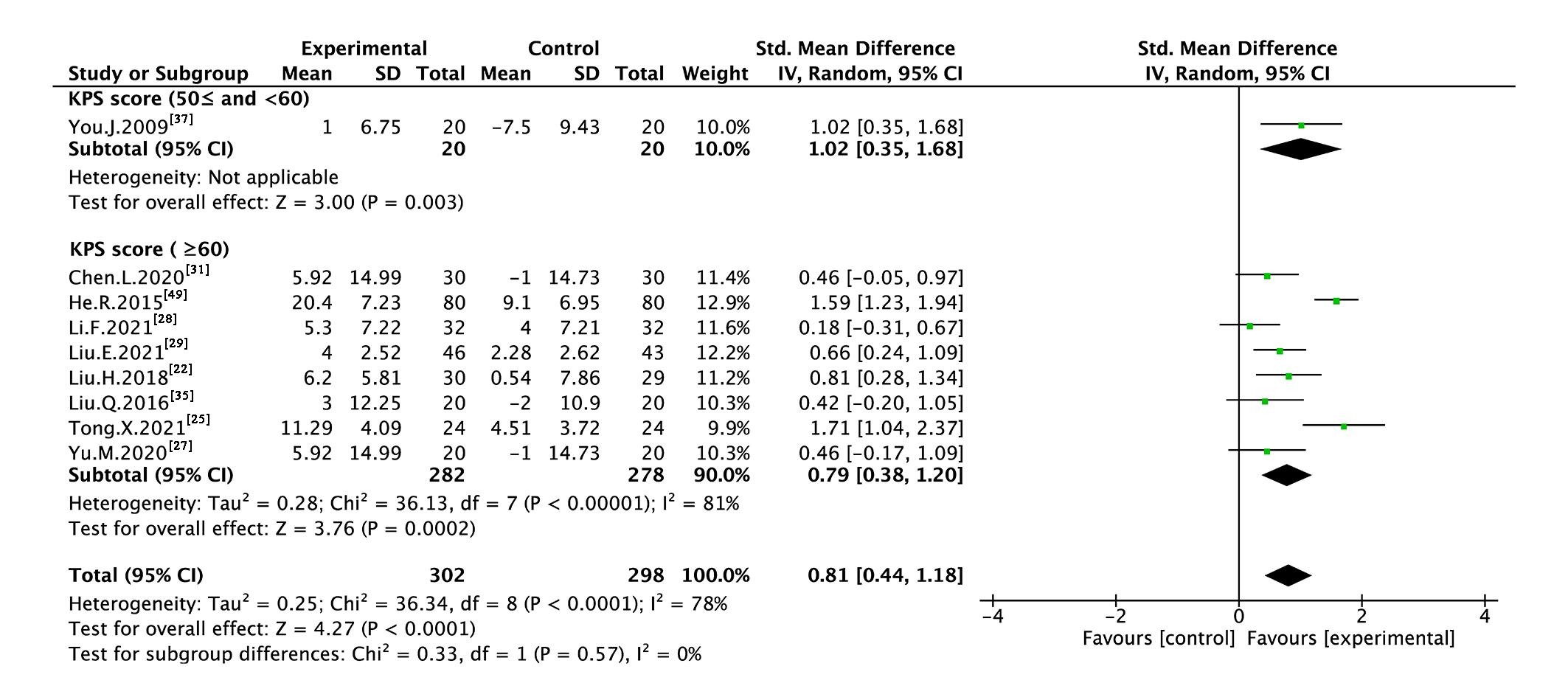

Supplement: Supplementary file 2 [file DataSheet_2.zip › Supplementary material 2/Figure S1.tiff]

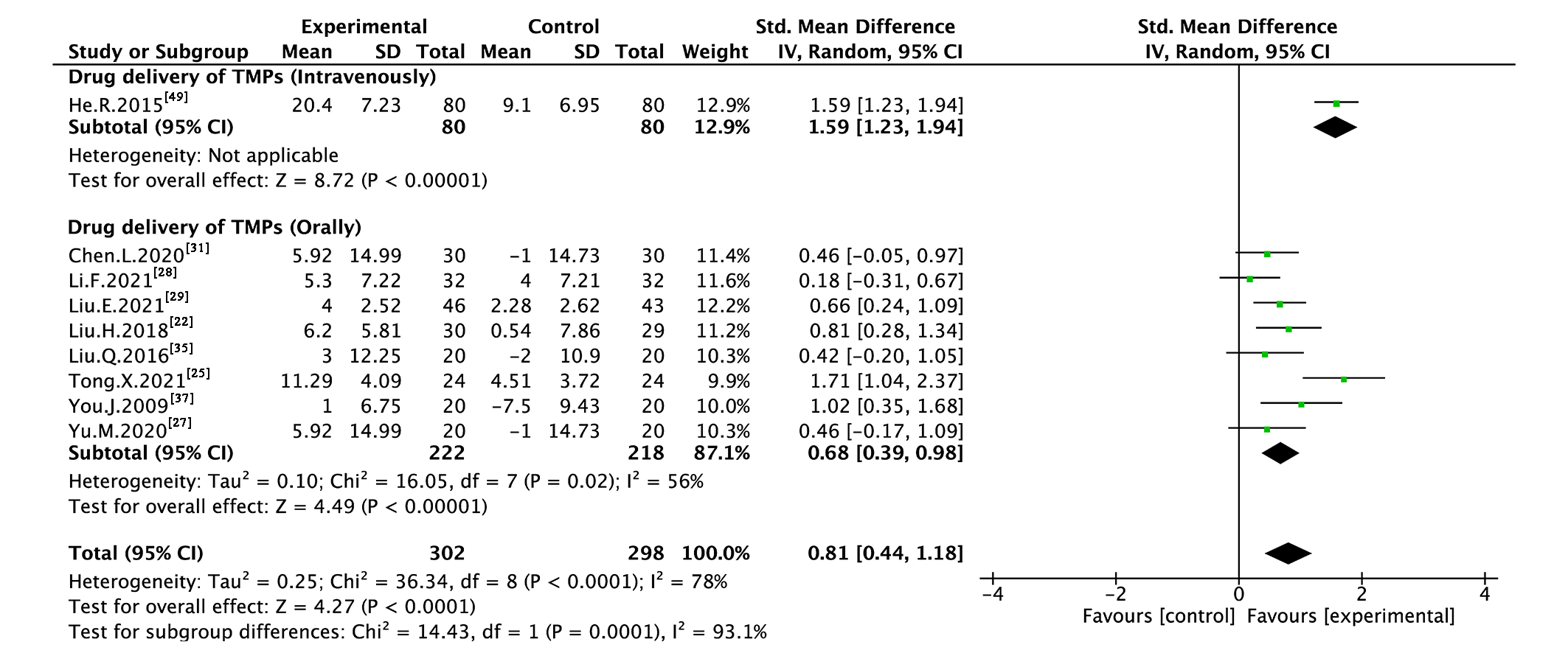

Supplement: Supplementary file 2 [file DataSheet_2.zip › Supplementary material 2/Figure S2.tiff]

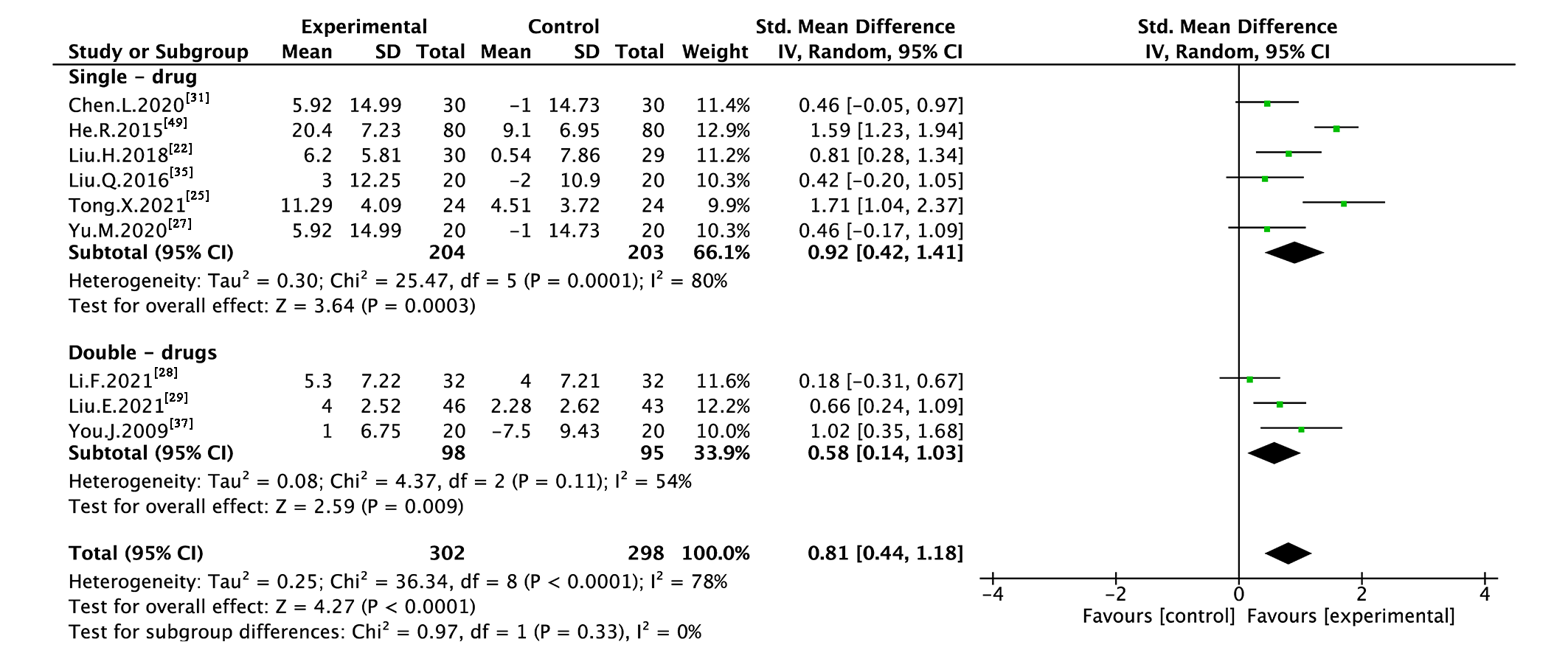

Supplement: Supplementary file 2 [file DataSheet_2.zip › Supplementary material 2/Figure S3.tiff]

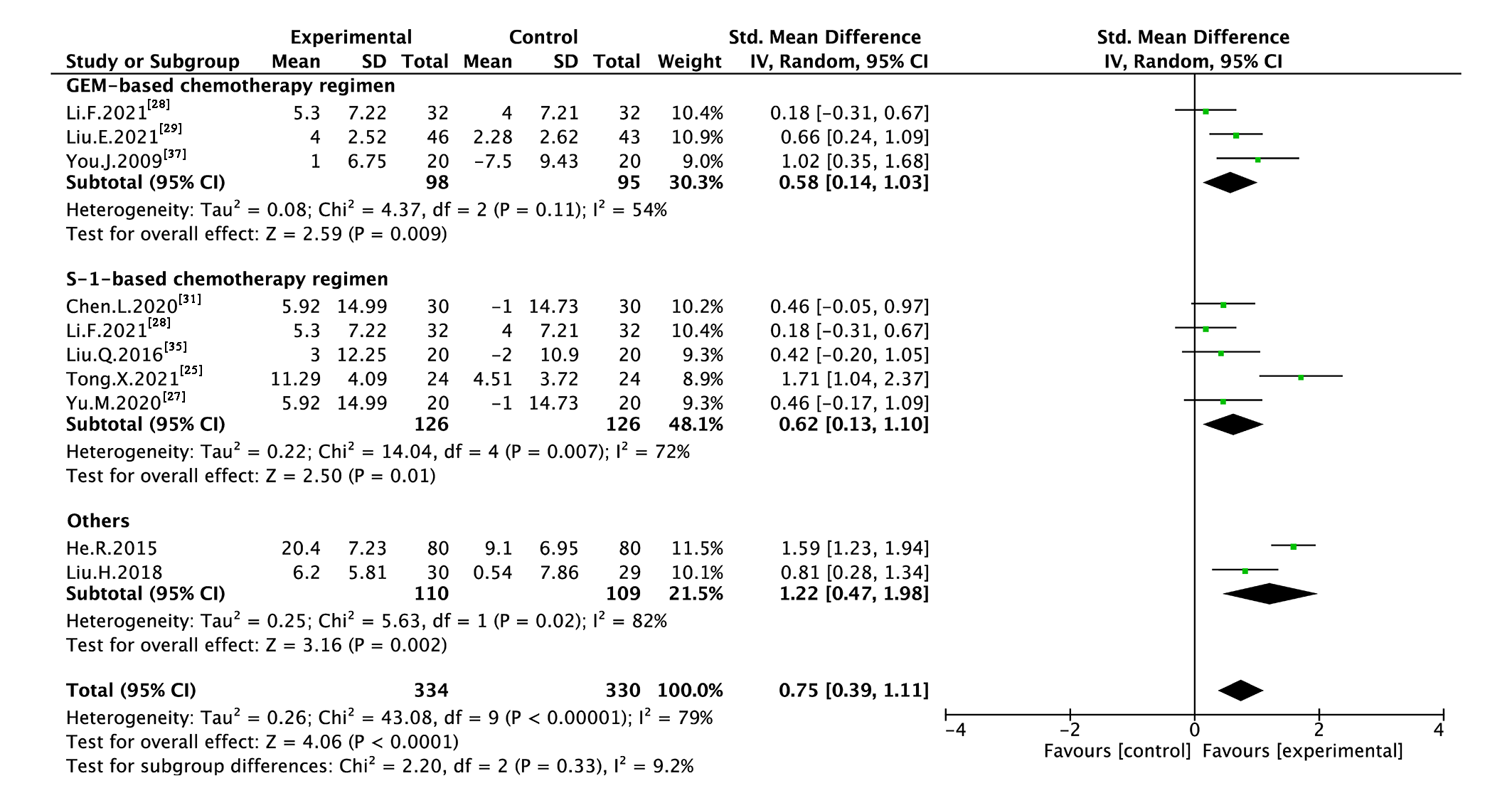

Supplement: Supplementary file 2 [file DataSheet_2.zip › Supplementary material 2/Figure S4.tiff]

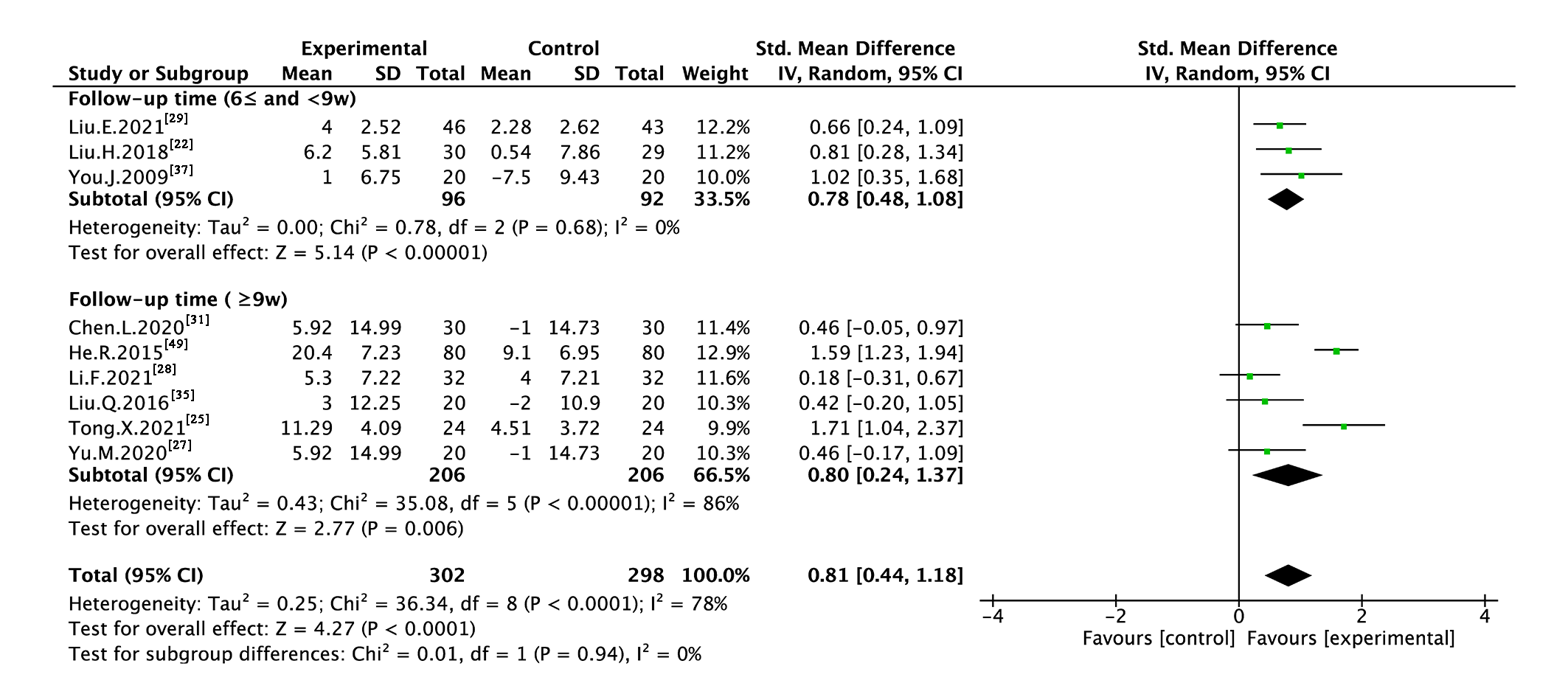

Supplement: Supplementary file 2 [file DataSheet_2.zip › Supplementary material 2/Figure S5.tiff]

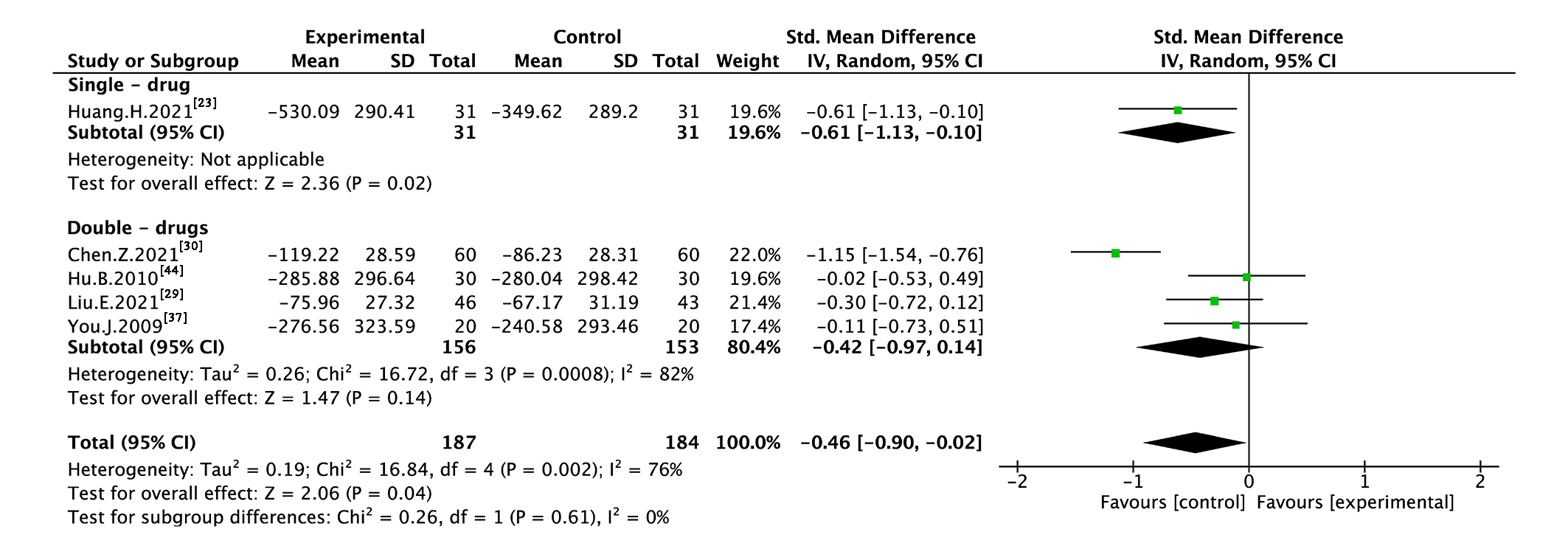

Supplement: Supplementary file 3 [file DataSheet_3.zip › Supplementary material 3/Figure S6.tiff]

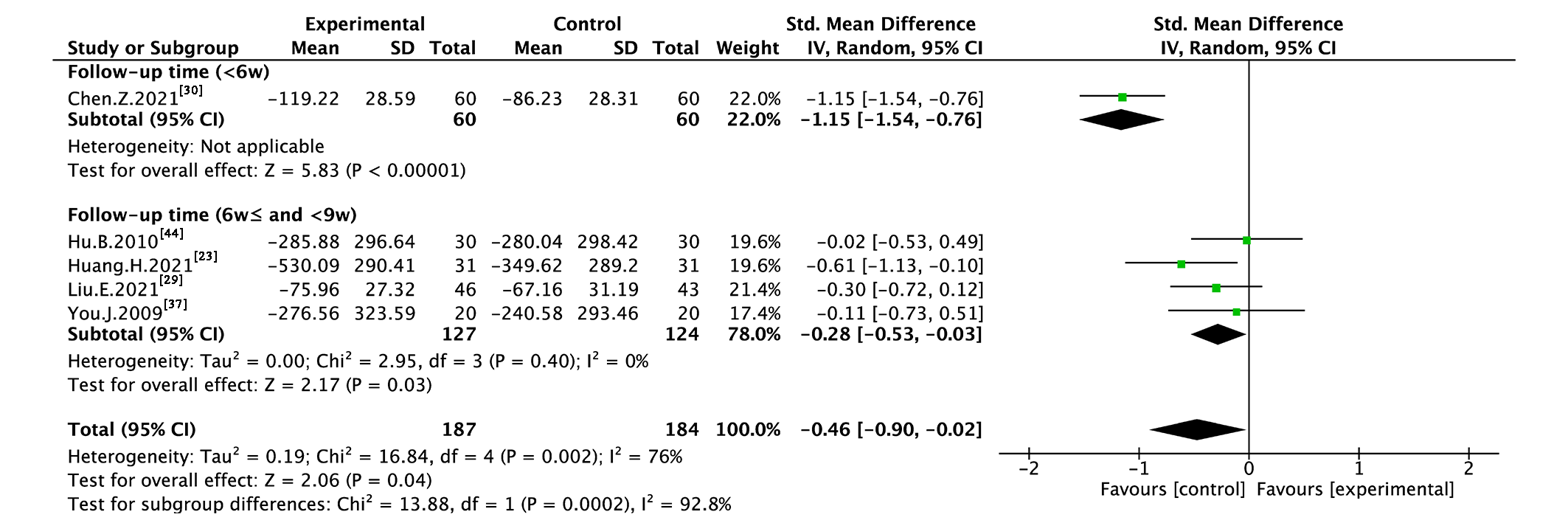

Supplement: Supplementary file 3 [file DataSheet_3.zip › Supplementary material 3/Figure S7.tiff]

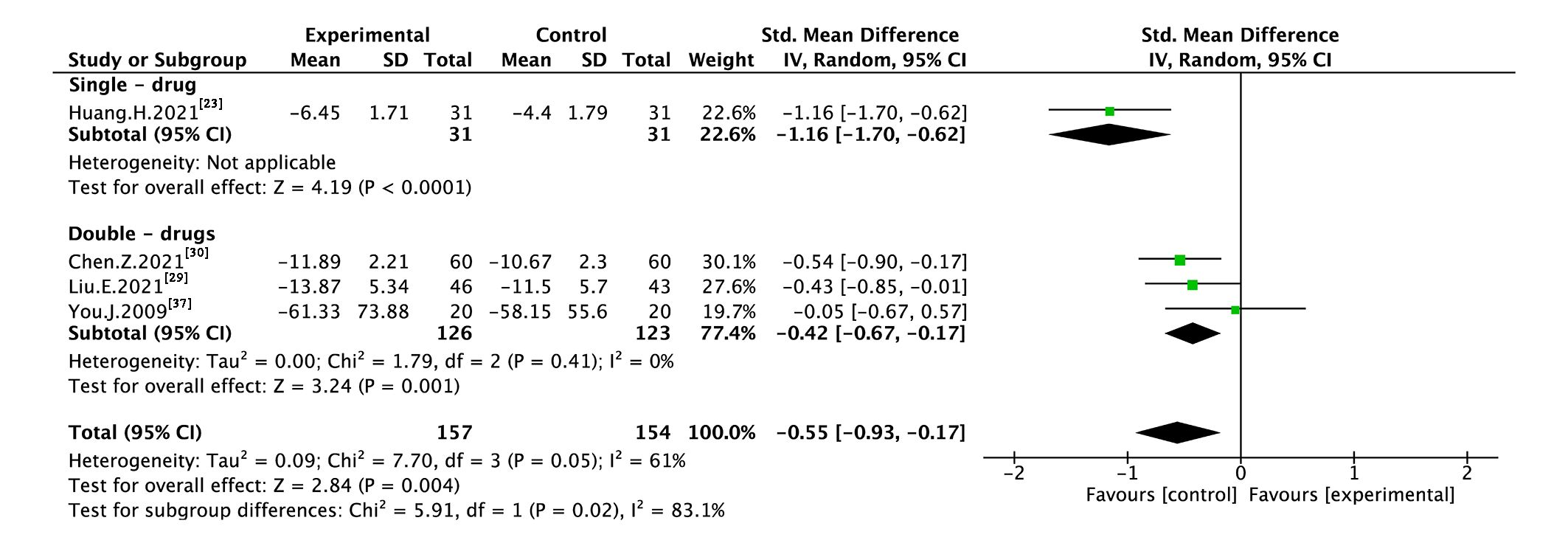

Supplement: Supplementary file 3 [file DataSheet_3.zip › Supplementary material 3/Figure S8.tiff]

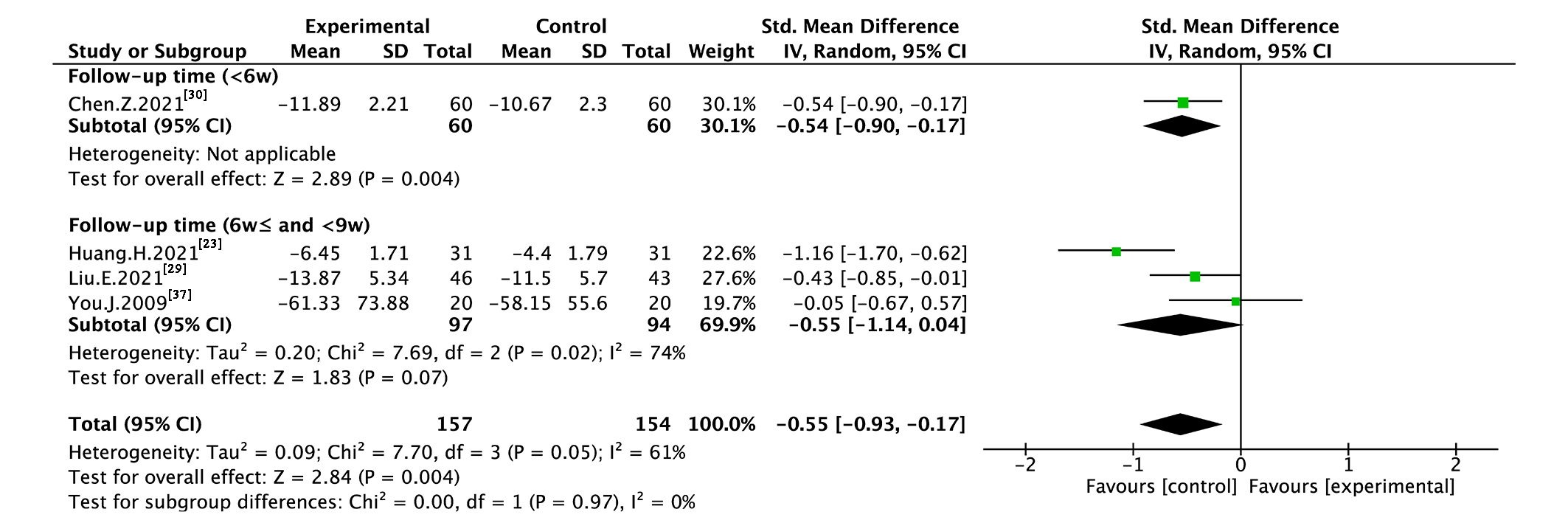

Supplement: Supplementary file 3 [file DataSheet_3.zip › Supplementary material 3/Figure S9.tiff]

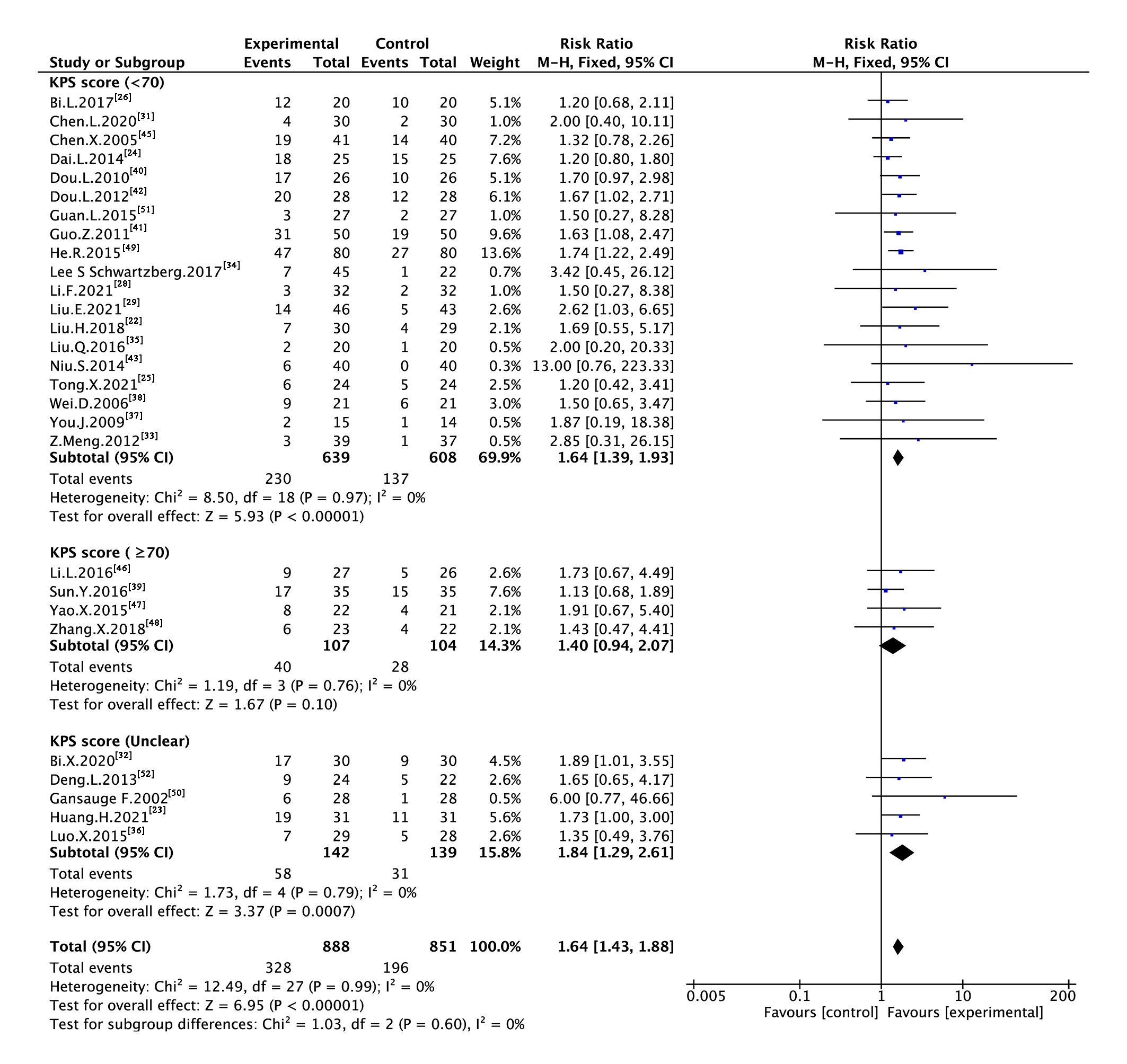

Supplement: Supplementary file 4 [file DataSheet_4.zip › Supplementary material 4/Figure S10.tiff]

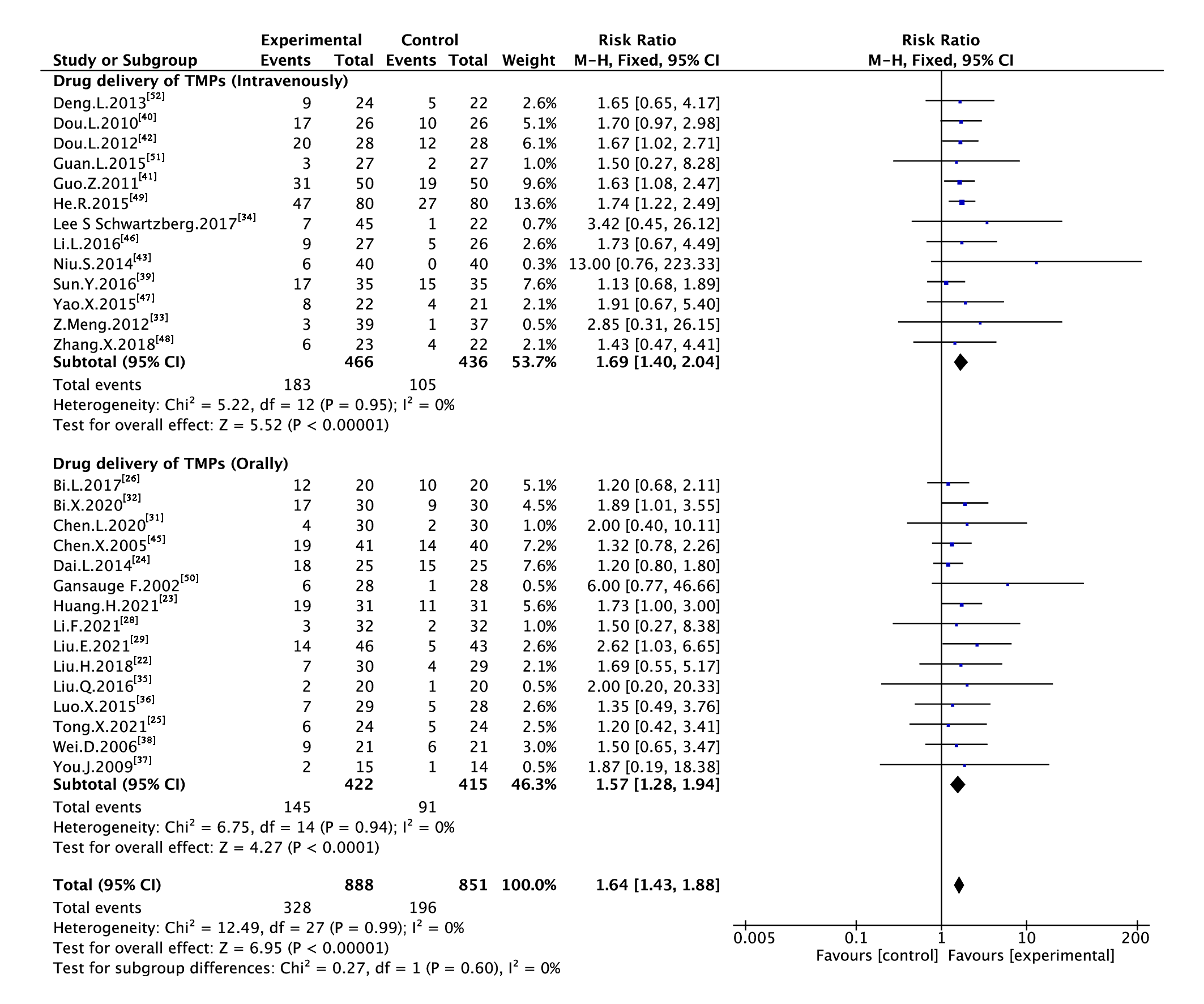

Supplement: Supplementary file 4 [file DataSheet_4.zip › Supplementary material 4/Figure S11.tiff]

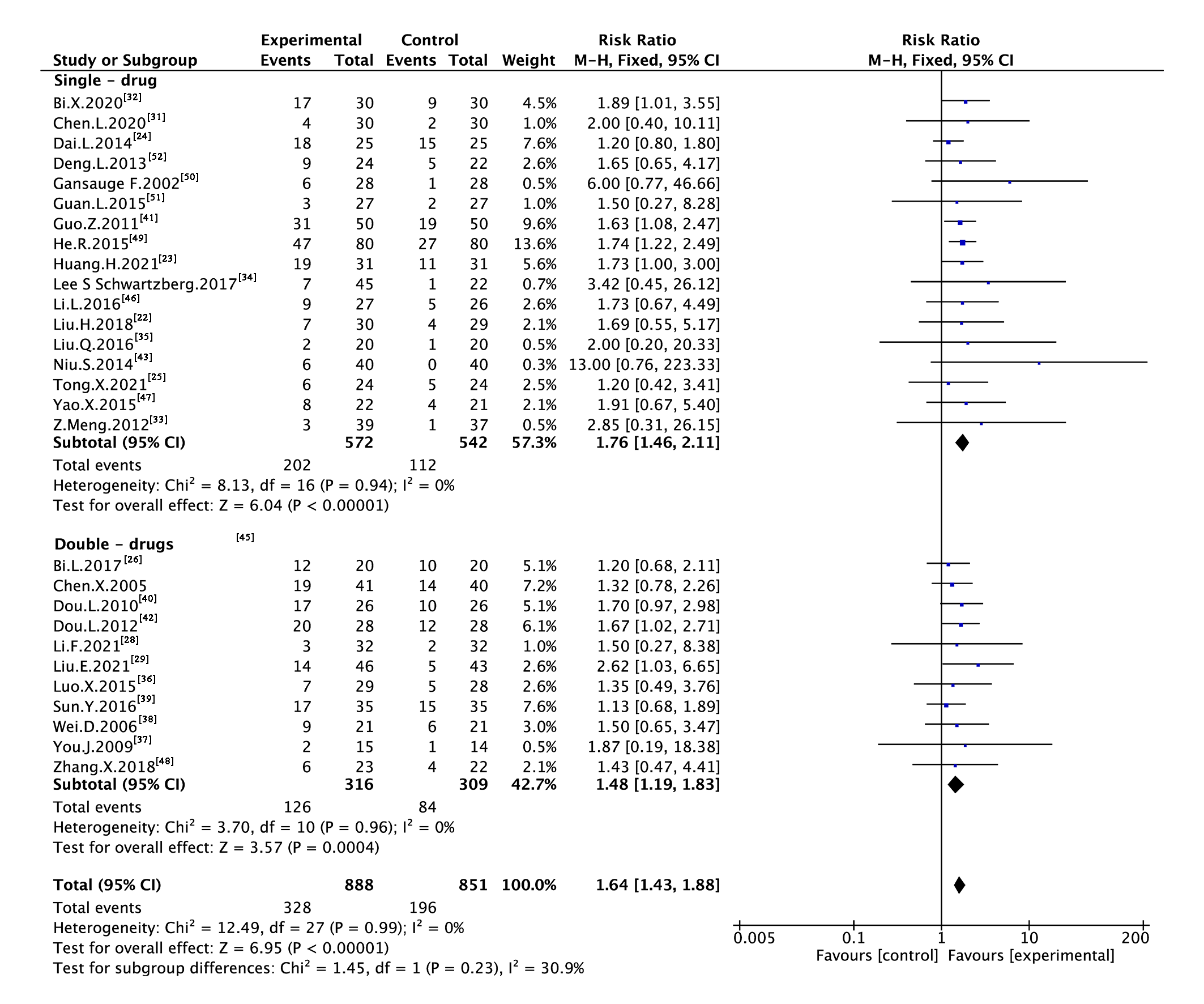

Supplement: Supplementary file 4 [file DataSheet_4.zip › Supplementary material 4/Figure S12.tiff]

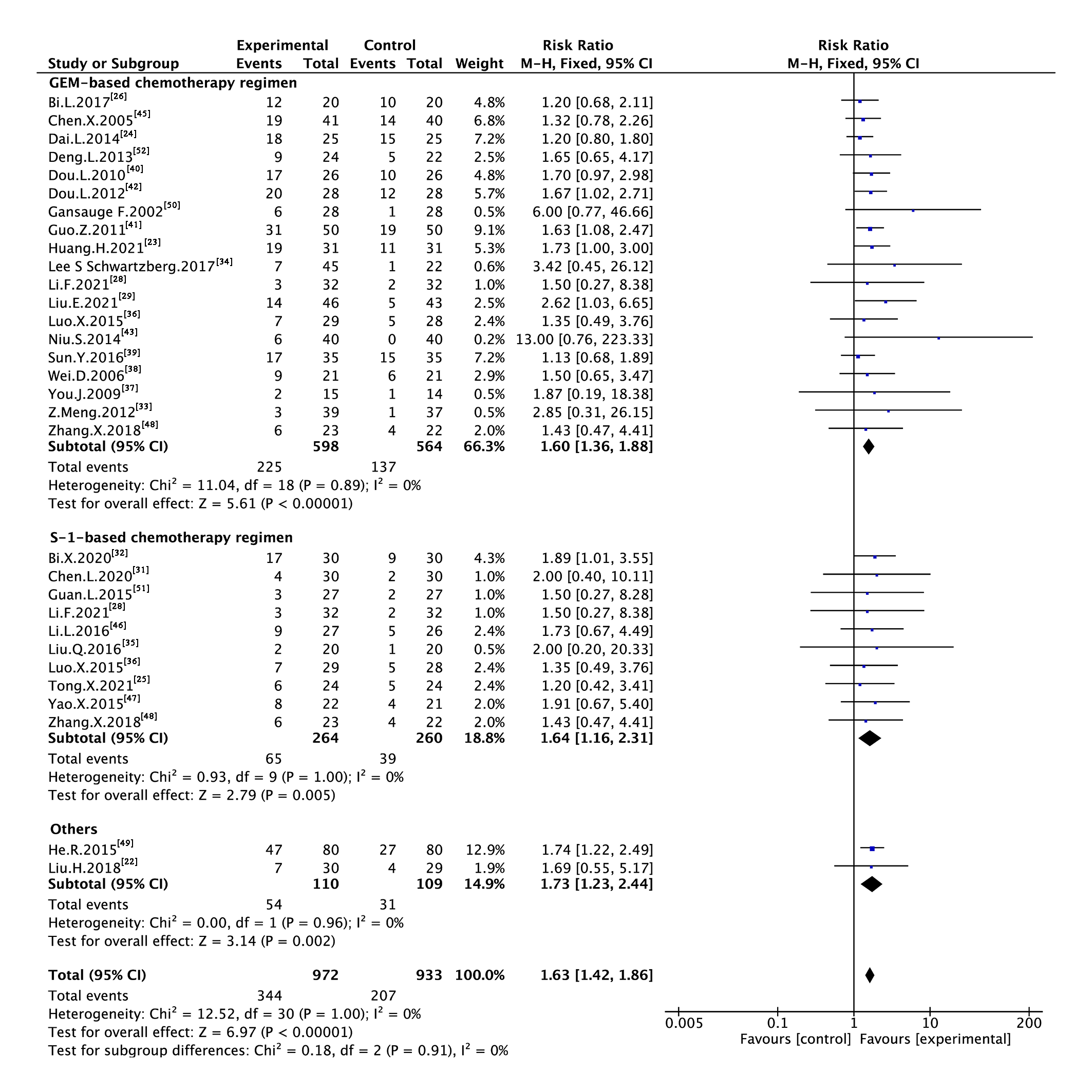

Supplement: Supplementary file 4 [file DataSheet_4.zip › Supplementary material 4/Figure S13.tiff]

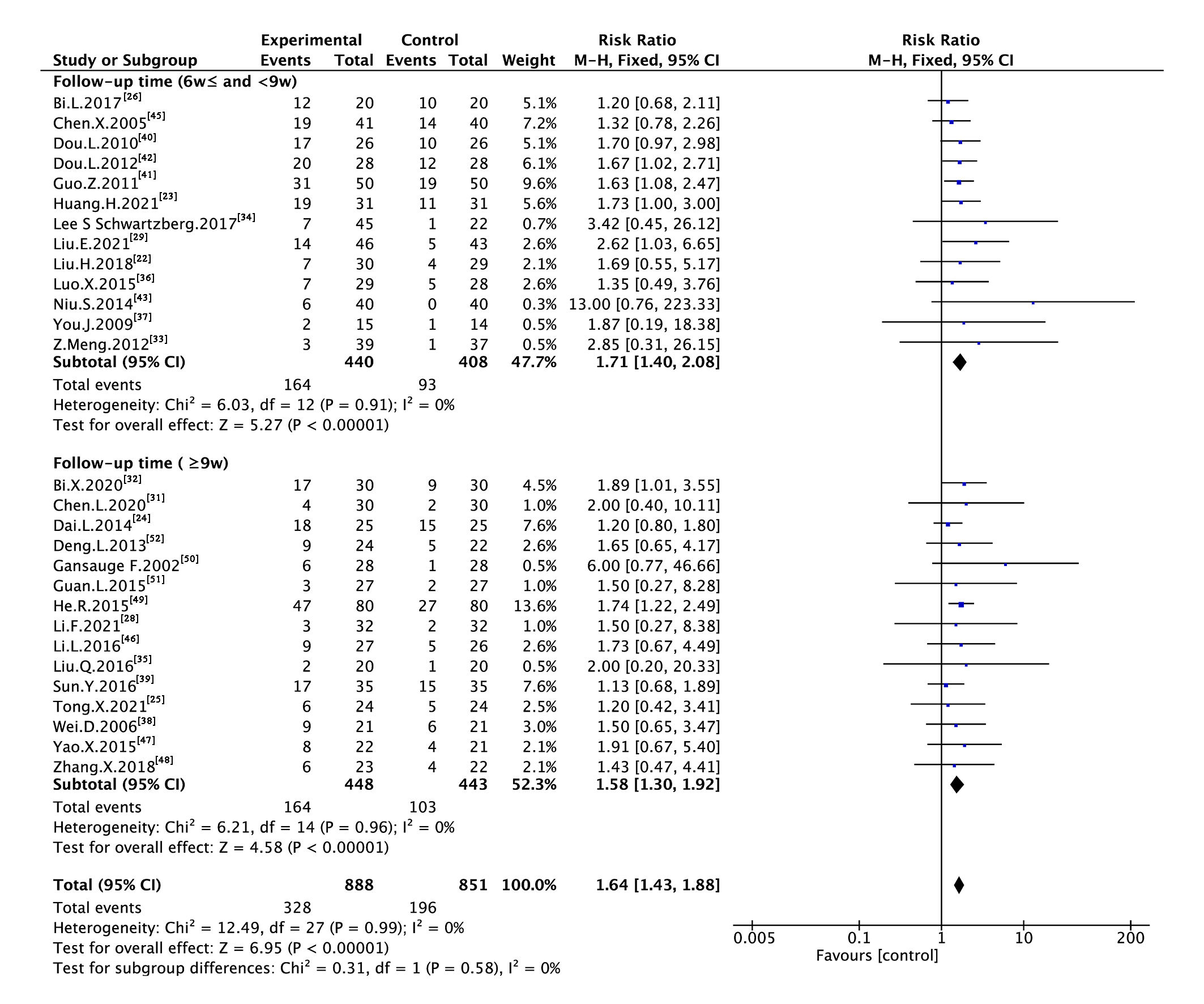

Supplement: Supplementary file 4 [file DataSheet_4.zip › Supplementary material 4/Figure S14.tiff]

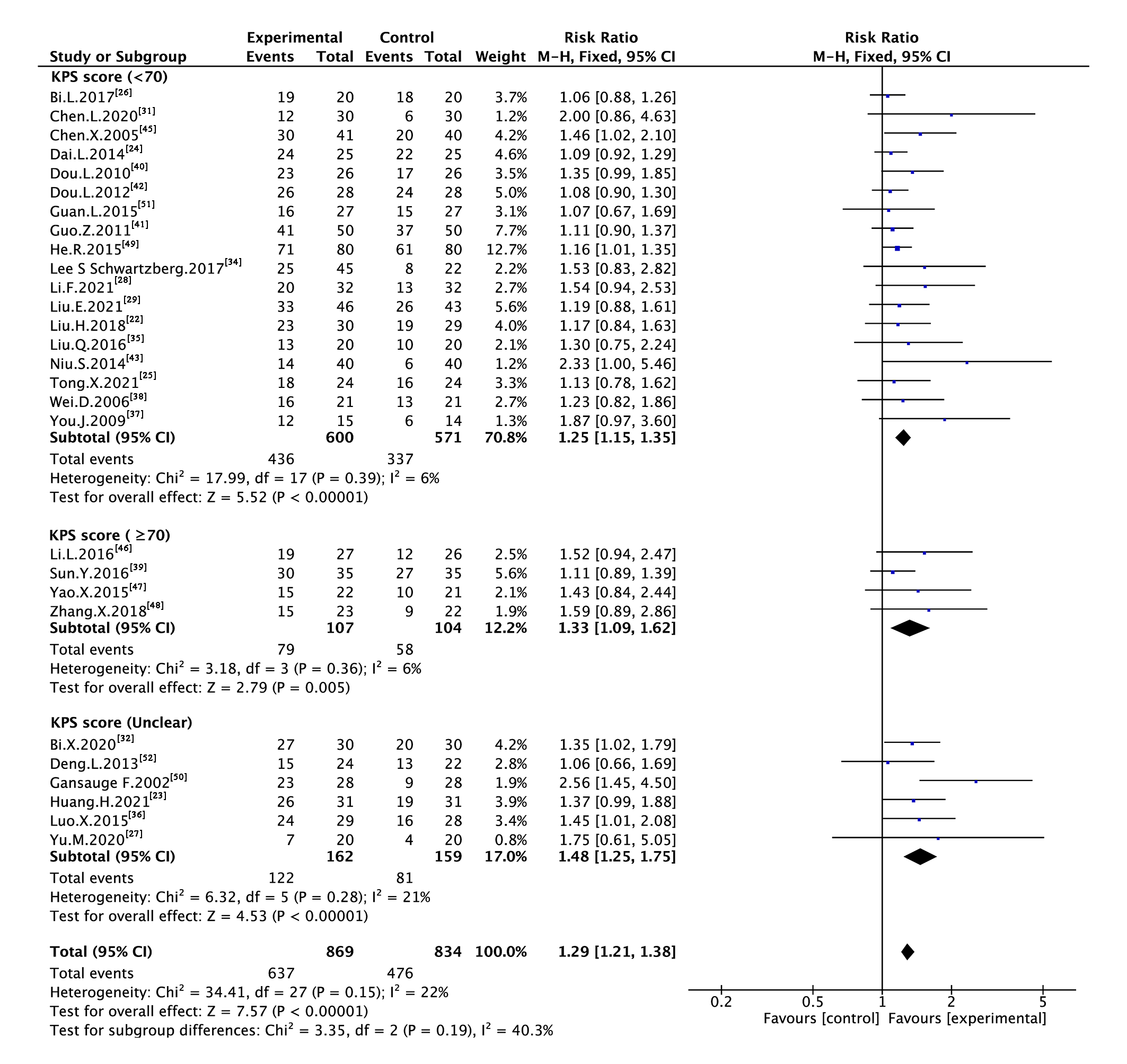

Supplement: Supplementary file 4 [file DataSheet_4.zip › Supplementary material 4/Figure S15.tiff]

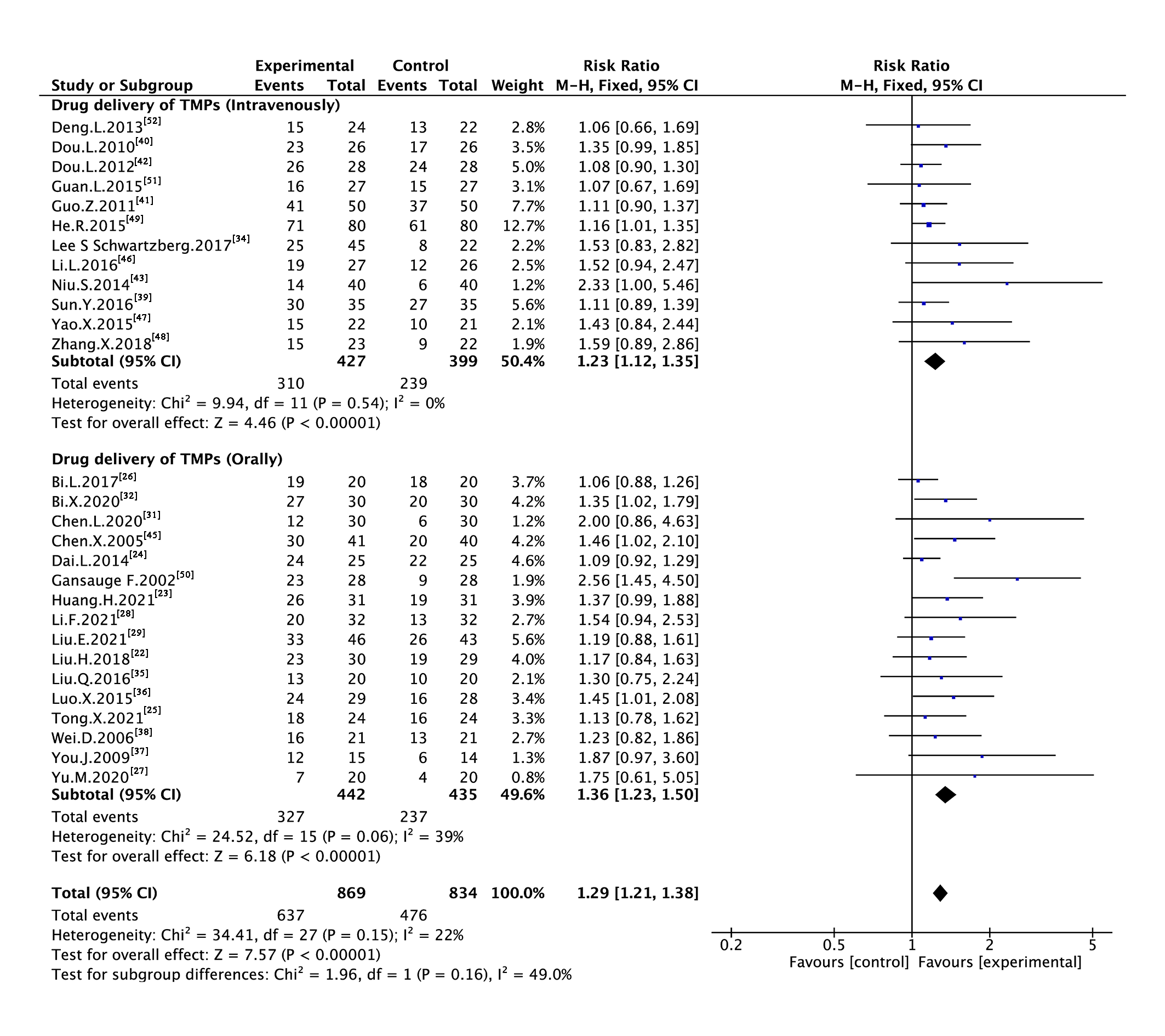

Supplement: Supplementary file 4 [file DataSheet_4.zip › Supplementary material 4/Figure S16.tiff]

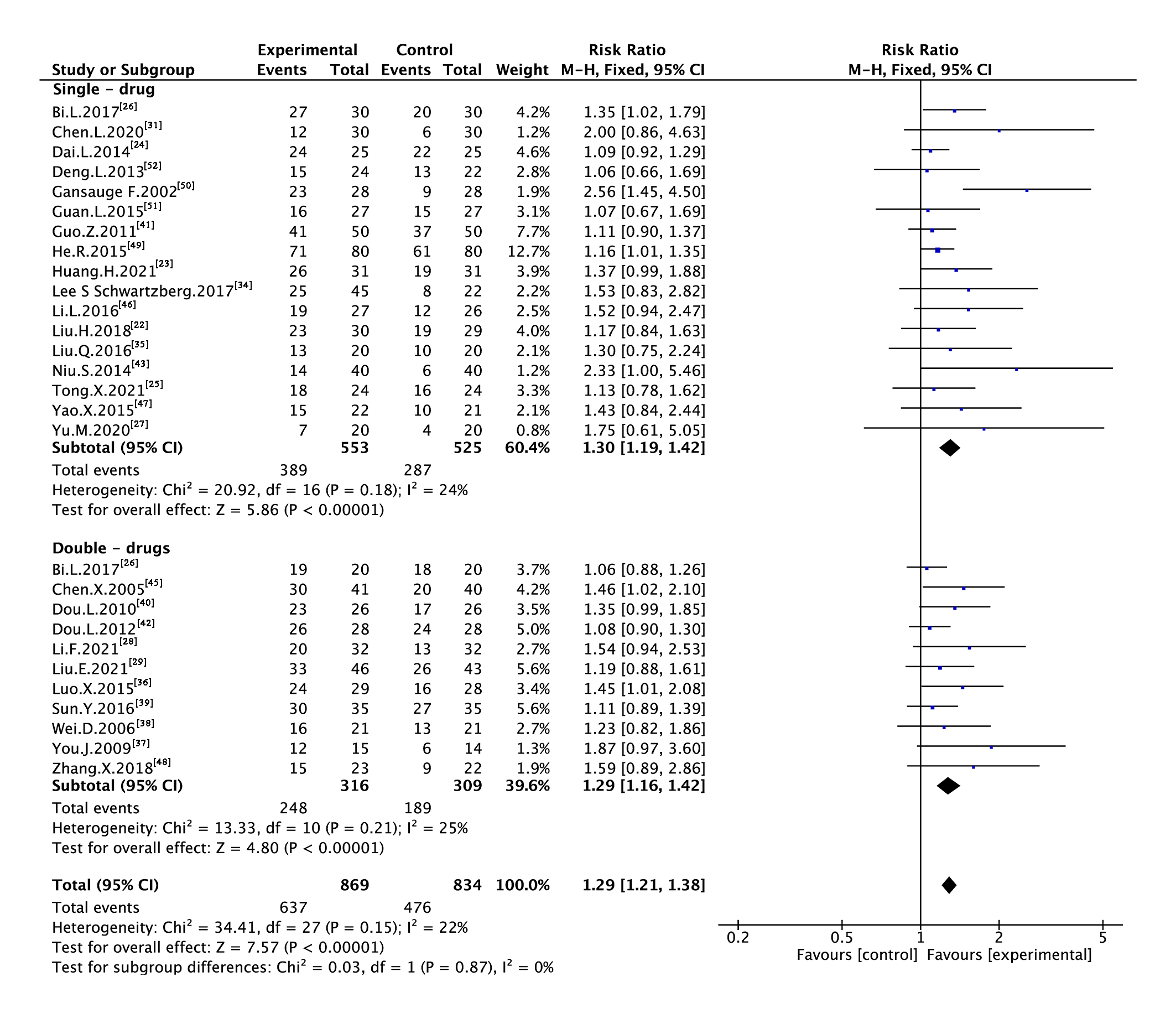

Supplement: Supplementary file 4 [file DataSheet_4.zip › Supplementary material 4/Figure S17.tiff]

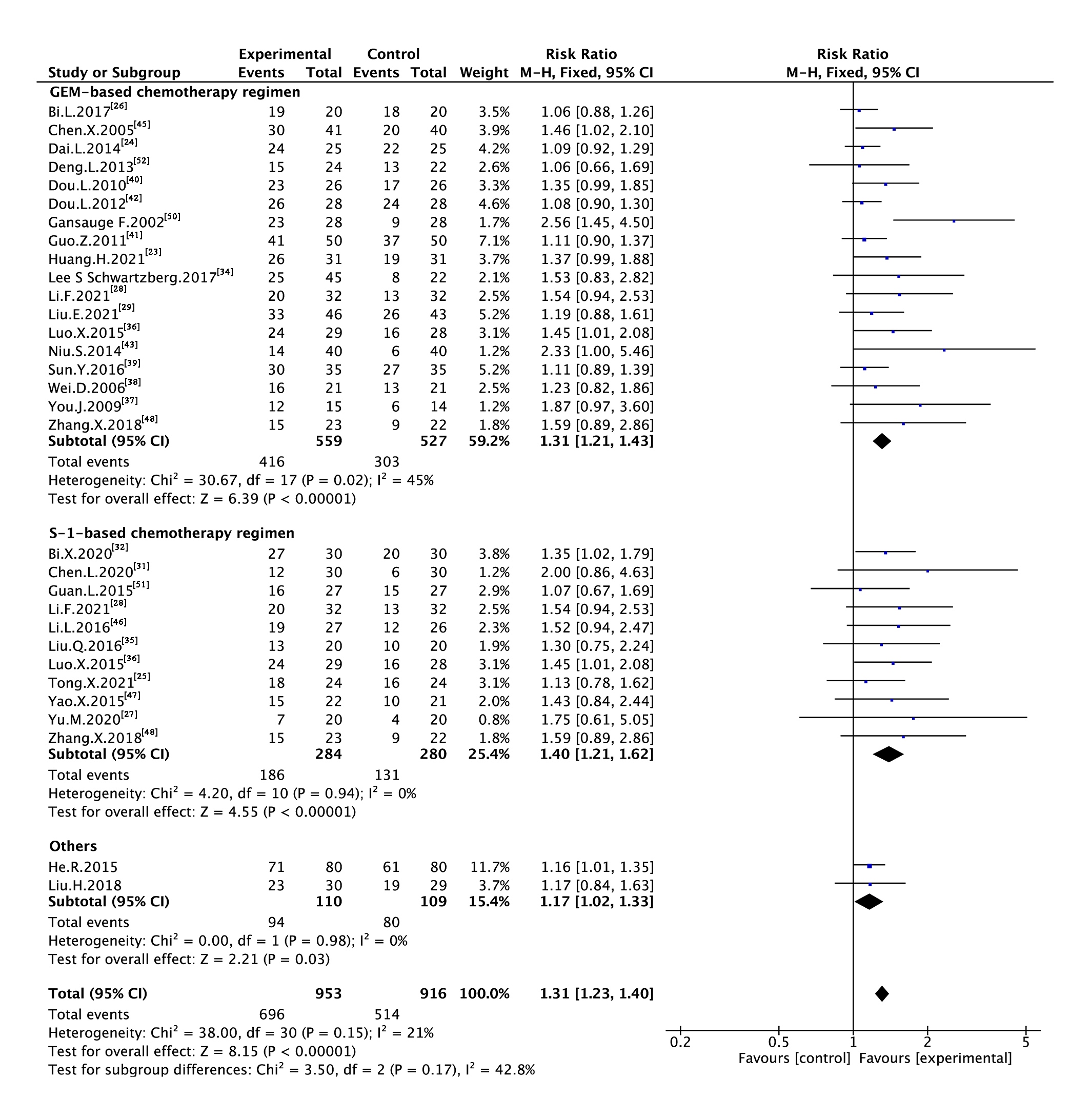

Supplement: Supplementary file 4 [file DataSheet_4.zip › Supplementary material 4/Figure S18.tiff]

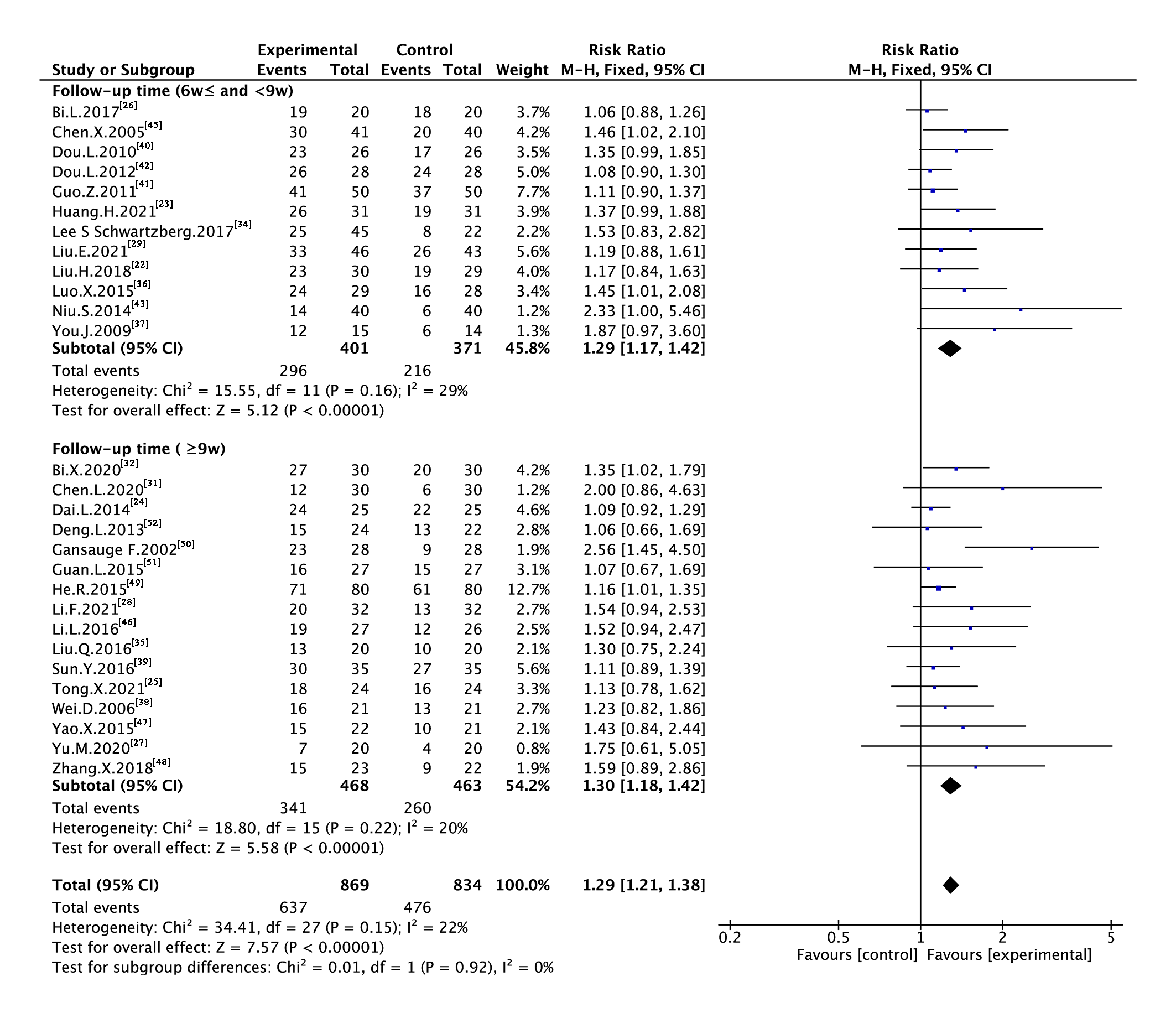

Supplement: Supplementary file 4 [file DataSheet_4.zip › Supplementary material 4/Figure S19.tiff]

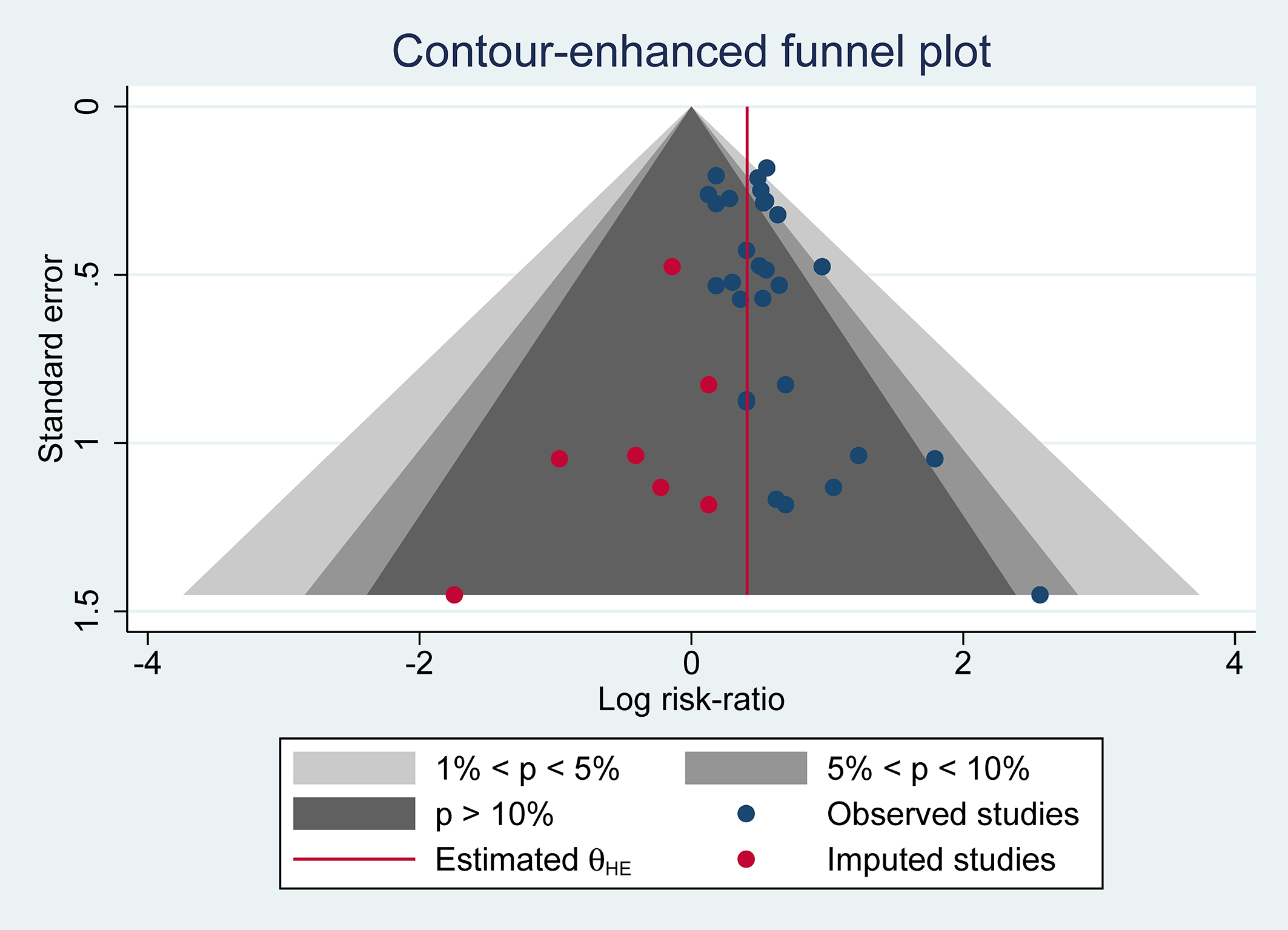

Supplement: Supplementary file 5 [file DataSheet_5.zip › Supplementary material 5/Figure S20.tiff]

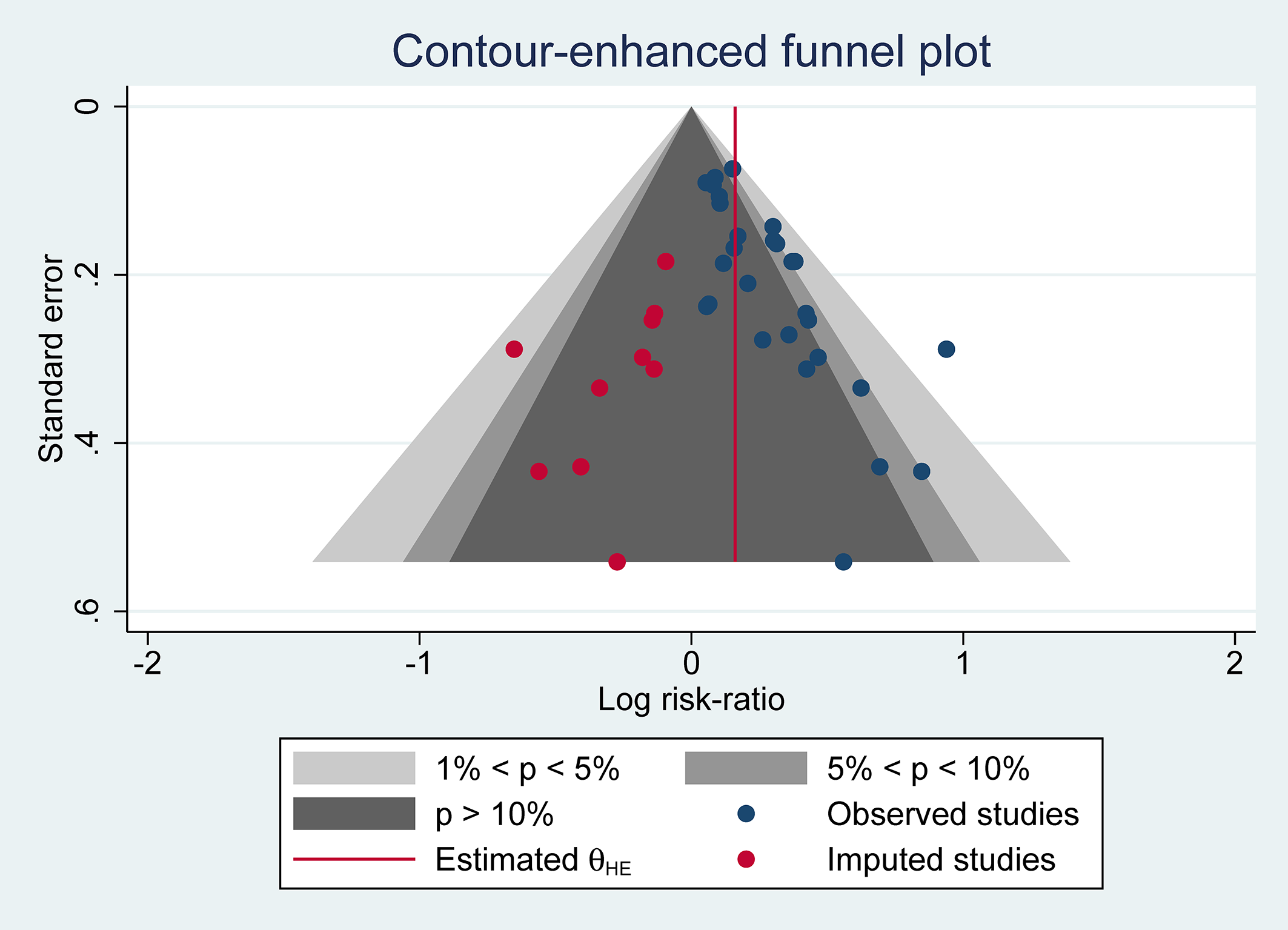

Supplement: Supplementary file 5 [file DataSheet_5.zip › Supplementary material 5/Figure S21.tiff]
