# Supplementary material for: Clinical Efficacy and Safety of Traditional Medicine Preparations Combined With Chemotherapy for Advanced Pancreatic Cancer: A Systematic Review and Meta-Analysis
Source: Front Oncol. 2022 Feb 23;12:828450. doi: 10.3389/fonc.2022.828450 (PMC8904728; doi:10.3389/fonc.2022.828450)
Supplement: Supplementary file 3 [file DataSheet_3.zip › Supplementary material 3/Supplementary material 3.docx]

**Supplementary material 3. Subgroup analysis results of cancer biomarkers (Table S2-3)**

**Table S2. Subgroup analysis of CA19-9**

| **Subgroups** | **Number of trials** | **SMD (95% CI)** | **Z** | ***p*** | **Heterogeneity** | | **TSD** |
| --- | --- | --- | --- | --- | --- | --- | --- |
|  |  |  |  |  | **I²** | ***P_h_*** |  |
| **Table S2a. Subgroups analysis according to the number of chemotherapy drug (Fig S6)** | | | | | | |  |
| Single - drug | 1 | -0.61 [-1.13, -0.10] | 2.36 | 0.02 | Not applicable | Not applicable | 0% |
| Double - drugs | 4 | -0.42 [-0.97, 0.14] | 1.47 | 0.14 | 82% | 0.0008 |  |
| **Table S2b. Subgroups analysis according to follow-up time (Fig S7)** | | | | | | |  |
| <6w | 1 | -1.15 [-1.54, -0.76] | 5.83 | <0.00001 | Not applicable | Not applicable | 92.8% |
| 6w≤ and <9w | 4 | -0.28 [-0.53, -0.03] | 2.17 | 0.03 | 0% | 0.40 |  |

**Note:** RR: risk ratio, CI: confidence interval, QoL: quality of life, PT: primary treatment, TSD: Test for subgroup differences.

**Table S3. Subgroup analysis of CEA**

| **Subgroups** | **Number of trials** | **SMD (95% CI)** | **Z** | ***p*** | **Heterogeneity** | | **TSD** |
| --- | --- | --- | --- | --- | --- | --- | --- |
|  |  |  |  |  | **I²** | ***P_h_*** |  |
| **Table S3a. Subgroups analysis according to the number of chemotherapy drug (Fig S8)** | | | | | | |  |
| Single - drug | 1 | -1.16 [-1.70, -0.62] | 4.19 | <0.0001 | Not applicable | Not applicable | 83.1% |
| Double - drugs | 3 | -0.42 [-0.67, -0.17] | 3.24 | 0.001 | 0% | 0.41 |  |
| **Table S3b. Subgroups analysis according to follow-up time (Fig S9)** | | | | | | |  |
| <6w | 1 | -0.54 [-0.90, -0.17] | 2.89 | 0.004 | Not applicable | Not applicable | 0% |
| 6w≤ and <9w | 3 | -0.55 [-1.14, 0.04] | 1.83 | 0.07 | 74% | 0.02 |  |

**Note:** RR: risk ratio, CI: confidence interval, QoL: quality of life, PT: primary treatment, TSD: Test for subgroup differences.
